# Supplementary material for: Defining and cataloging variants in pangenome graphs
Source: Cell Genom. 2026 Aug 17;6(9):101327. doi: 10.1016/j.xgen.2026.101327 (PMC13576716; doi:10.1016/j.xgen.2026.101327)
Supplement: Supplementary file 1 [file mmc1.pdf]

Cell Genomics, Volume *6*

Supplemental information

Defining and cataloging variants in pangenome graphs

Pouria Salehi Nowbandegani, Shenghan Zhang, Haoyang Hu, Heng Li, and Luke J. O'Connor

Supplementary Figures

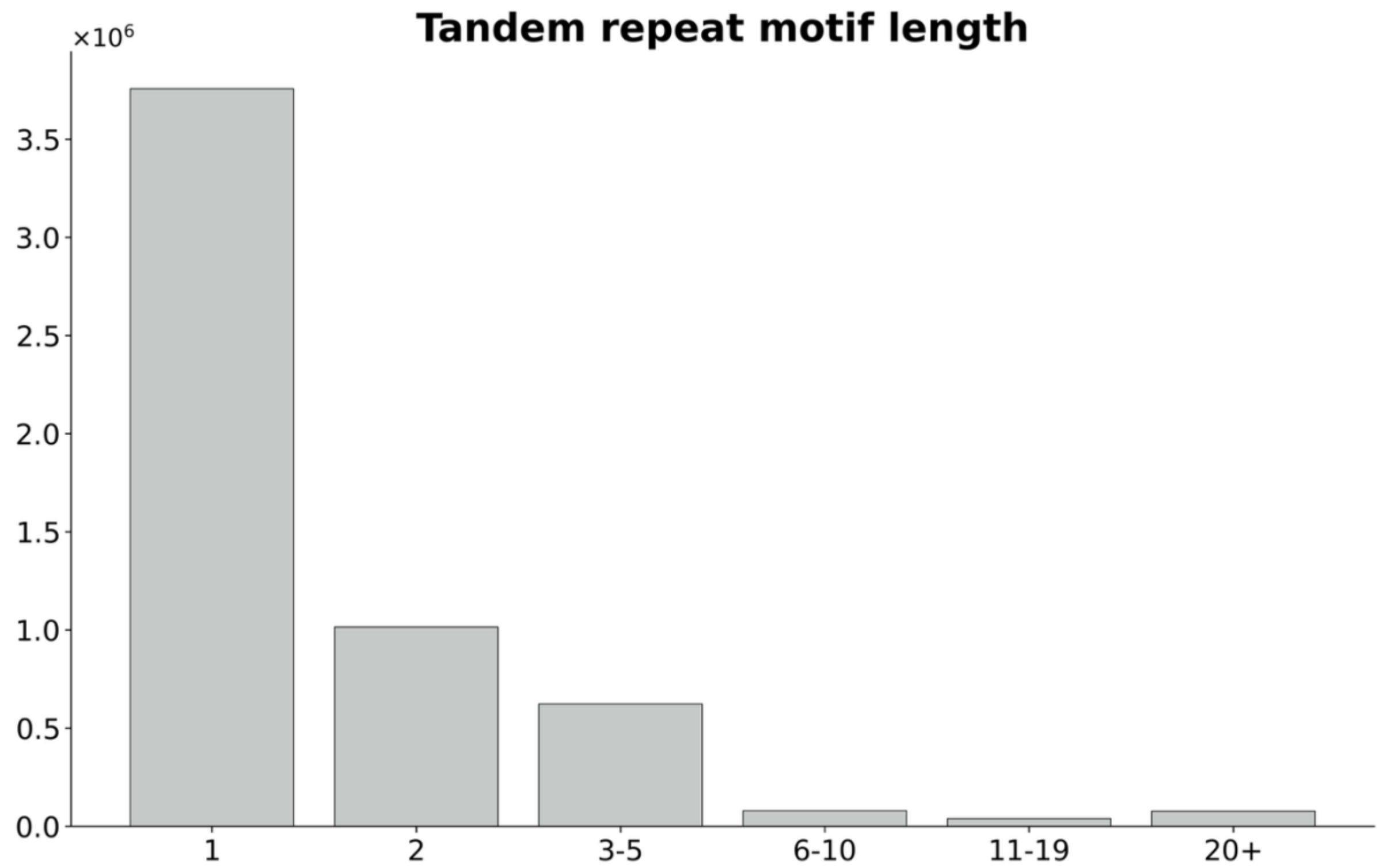

Figure S1. Distribution of tandem repeat motif lengths, related to Figure 2. Tandem repeat motif length was defined as the length of the ‘TR_motif’ field in the pantree VCF.

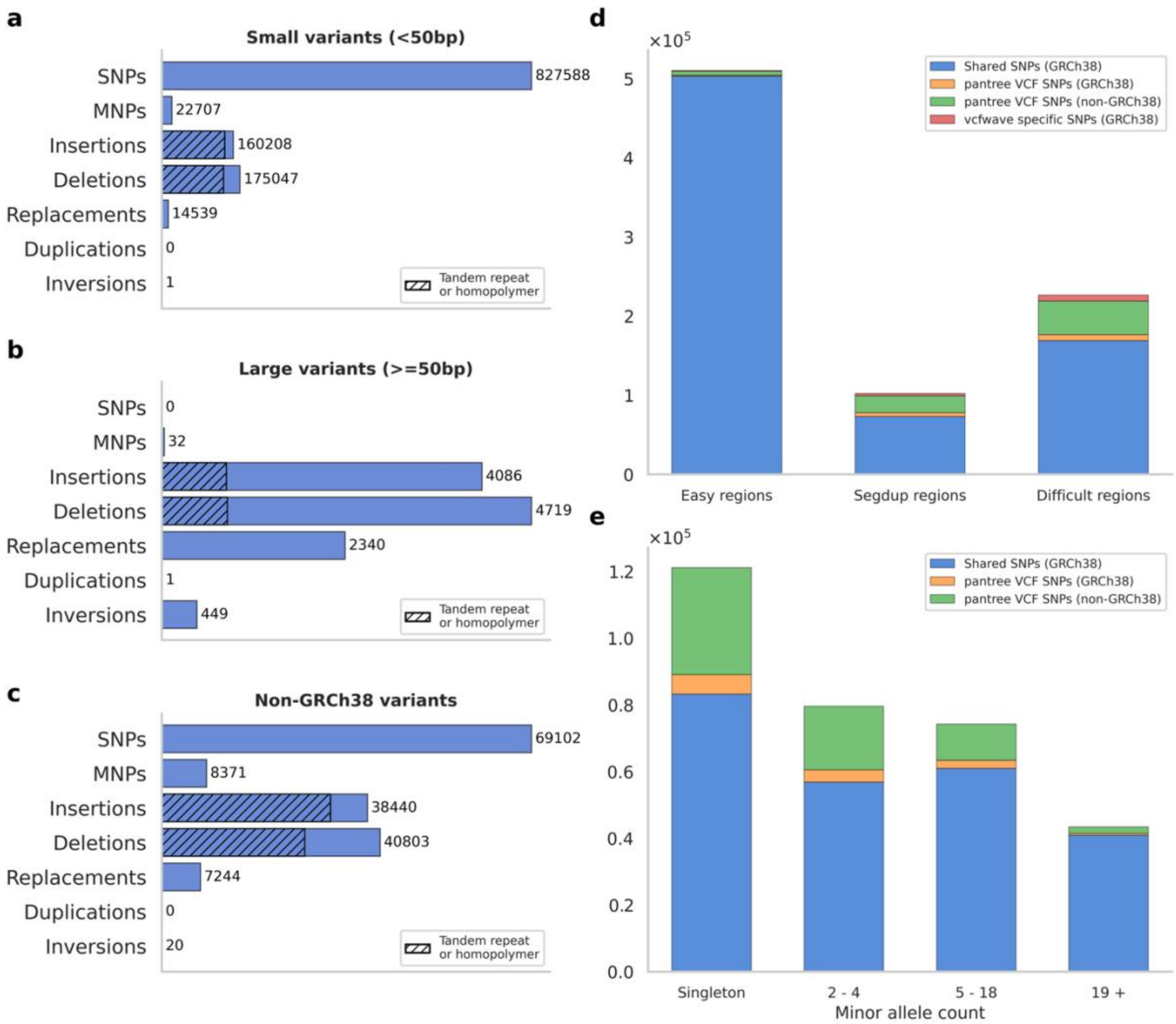

Figure S2. Variants on chromosome X, related to Figure 2 and Figure 3. (a) Number of small variants (<50 bp combined allele length) across seven non-overlapping variant types. Hatched area indicates the number of variants that cause a change in the copy number of a reference-tree basepair sequence. (b) Number of large variants. (c) Number of non-GRCh38 variants, defined as variant edges connecting two non-GRCh38 nodes. (d) Overlap of SNPs with those reported by *vcfwave*, across three non-overlapping genome annotations. (e) Minor allele count (the minimum of the reference allele count and alternative allele counts) out of 90, stratified by overlap with *vcfwave*.

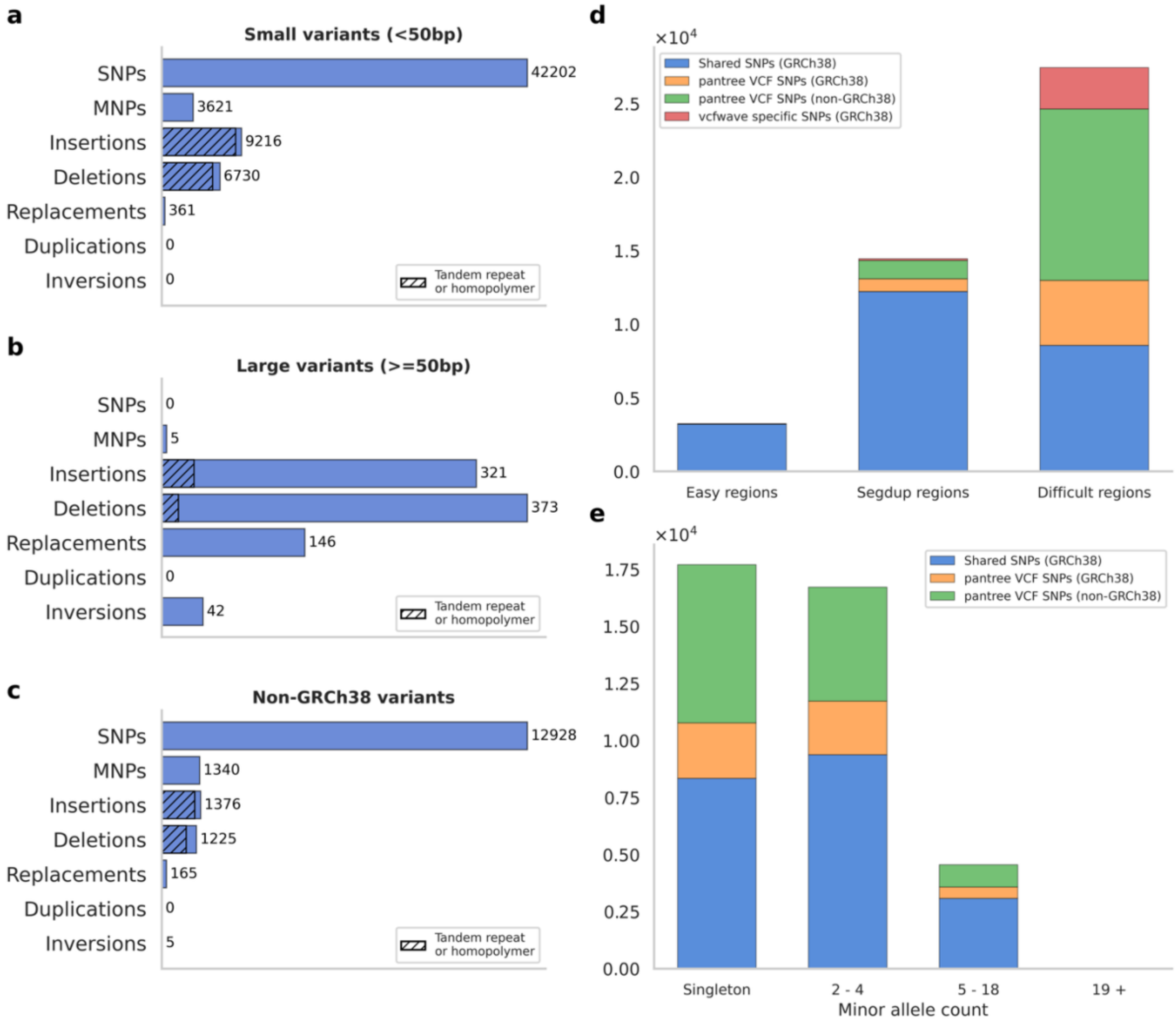

Figure S3. Variants on chromosome Y, related to Figure 2 and Figure 3. (a) Number of small variants (<50 bp combined allele length) across seven non-overlapping variant types. Hatched area indicates the number of variants that cause a change in the copy number of a reference-tree basepair sequence. (b) Number of large variants. (c) Number of non-GRCh38 variants, defined as variant edges connecting two non-GRCh38 nodes. (d) Overlap of SNPs with those reported by *vcfwave*, across three non-overlapping genome annotations. (e) Minor allele count (the minimum of the reference allele count and alternative allele counts) out of 21, stratified by overlap with *vcfwave*.

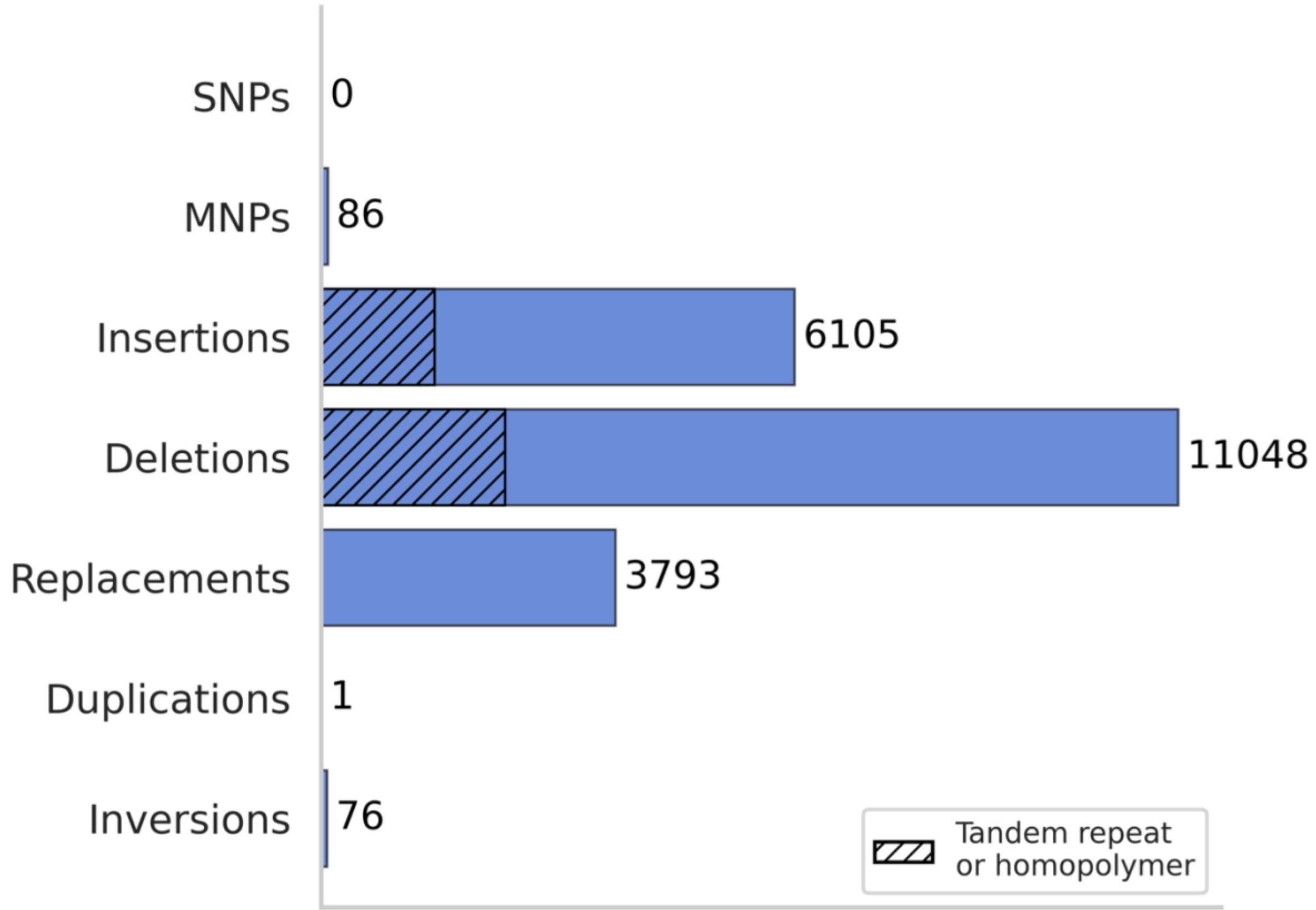

Figure S4. Large, common non-GRCh38 variants, related to Figure 2. Total counts per variant class (SNP, MNP, insertion, deletion, replacement, duplication, inversion) for non-GRCh38 variants with a minor allele count of at least 5 and a combined allele length of at least 50 bp. Hatched bars denote variants that cause a repeat expansion or contraction, including all duplications.

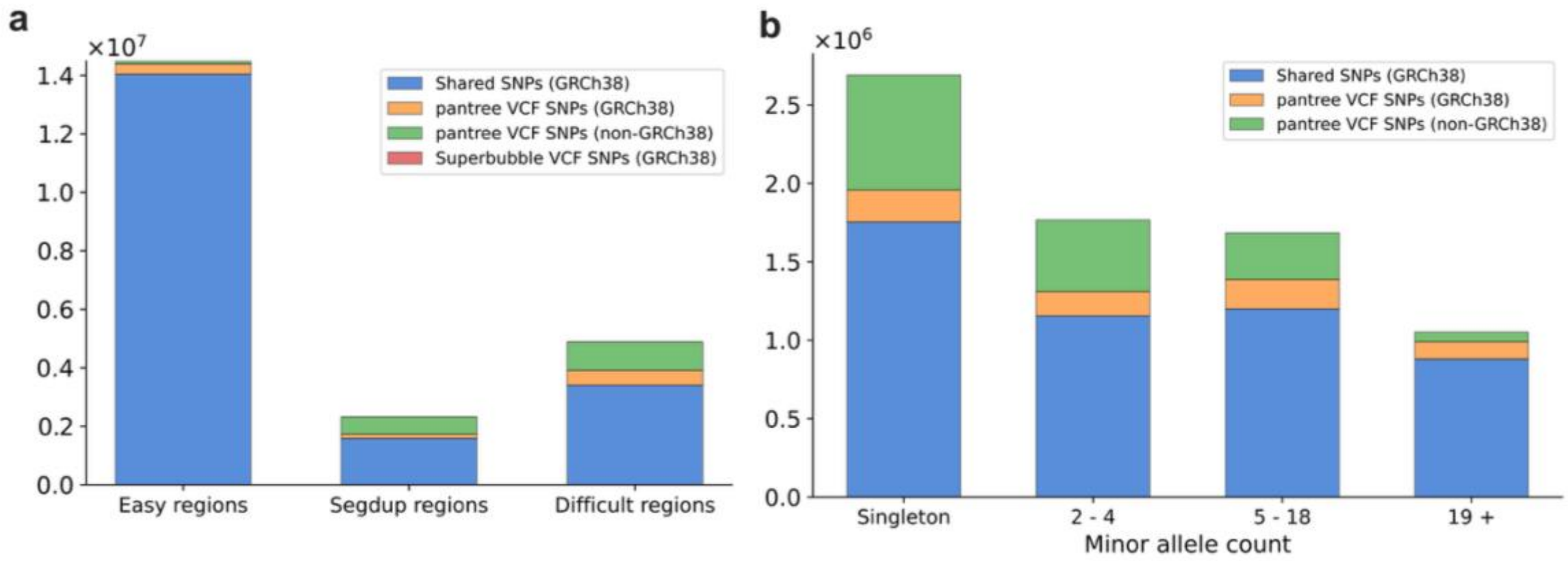

Figure S5. SNPs detected using pantree versus superbubbles, related to Figure 3. SNPs detected using pantree vs. superbubbles. (a) SNPs were partitioned by their VCF position into easy regions, segmental duplications, and other difficult regions. Bars indicate the number of SNPs detected using both methods, the number of GRCh38 and non-GRCh38 SNPs found by pantree only, and the number of SNPs found using superbubbles only (there were exactly 2 of these). (b) SNPs separated by their minor allele count, defined using pantree (the minimum value between reference allele counts and alternative allele count). Superbubble VCF specific SNPs were not included because they did not have allele counts reported.

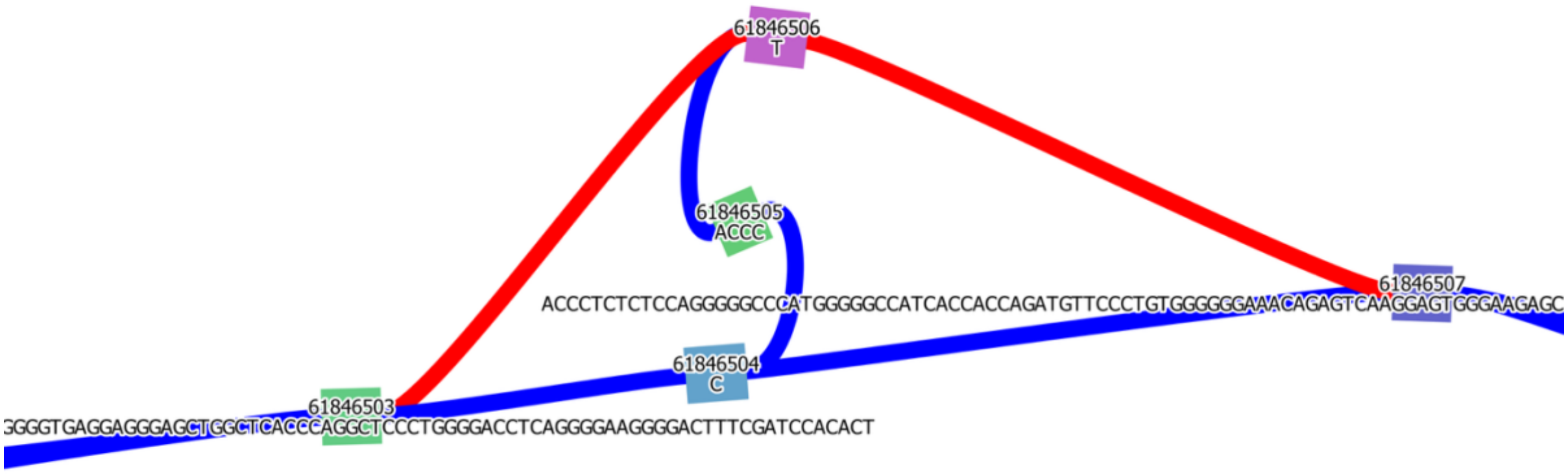

Figure S6. Superbubble-specific SNP on chromosome 6, related to Figure 3. Exactly two SNPs were detected as superbubbles without being called as SNPs by pantree. This superbubble, on chromosome 6, coincides with an assembly gap; there exists a terminal edge from the negative starting terminus to the node 61846506, which means that one walk starts in the middle of the superbubble from the node 61846506, then goes through the nodes 6184605 and 6184604, then to the pinch-point 6184603. Because superbubble alleles are required to pass through both ends of the superbubble, this walk, whose allele would be 'CACCCT', is not counted as an alternative allele, and the superbubble is classified as a SNP. pantree identifies an insertion ('ACCCT') and a deletion ('ACCC').

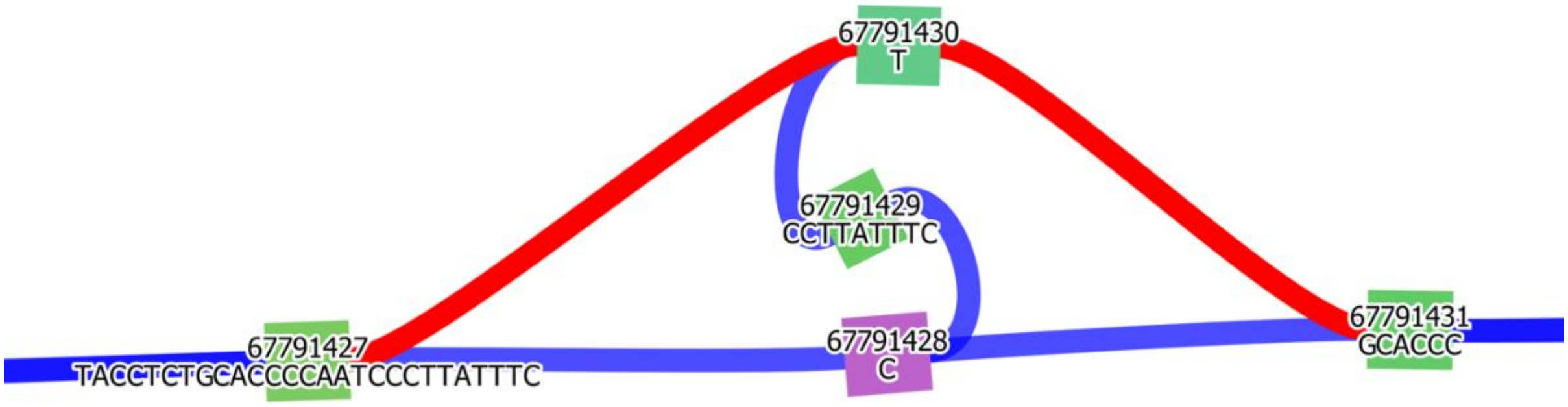

Figure S7. Superbubble-specific SNP on chromosome 7, related to Figure 3. Exactly two SNPs were detected as superbubbles without being called as SNPs by pantree. This superbubble, on chromosome 7, coincides with an assembly gap (similar to the SNP in Figure S6). There is a terminal edge from the node 67791428 to the negative ending terminus, and a walk which begins within node 67791431 following the negative orientation, then goes through the nodes 67791430 and 67791429, then ends at 67791428. Thus, the 'CCCTTATTTCT' allele did not show up as an alternative allele in this superbubble.

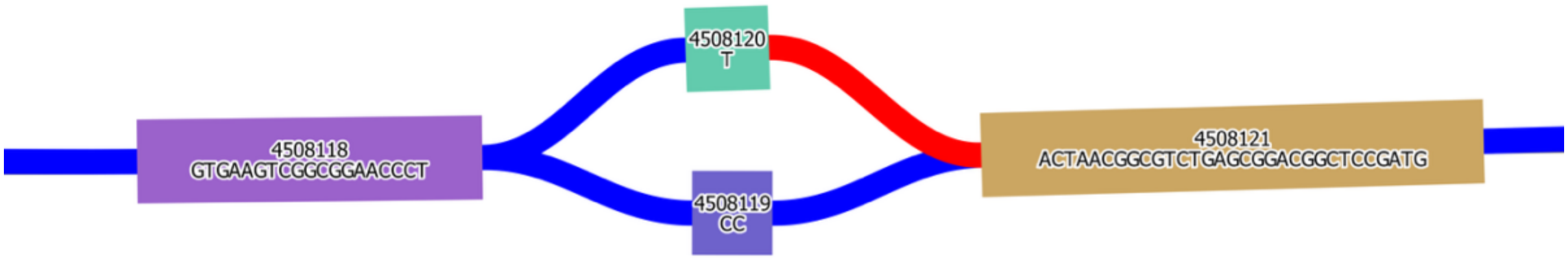


```
pantree VCF
CHROM POS       ID                  REF  ALT  INFO
chr1  248826452 <4508120>4508121    CC   T    NR=.
superbubble VCF
chr1  248826451 >4508118>4508121    TCC  TT
vcfwave
chr1  248826451 >4508118>4508121_1  TC   T
chr1  248826453 >4508118>4508121_2  C    T
```

Figure S8. vcfwave-specific SNP inconsistent with the graph, related to Figure 3. pantree identifies a replacement whose reference allele is CC and alternative allele is T, and the ‘raw’ superbubble VCF has a similar variant (except prepending the last letter of the branch point to both alleles). *vcfwave* produced a different alignment and identified two variants, including a C-to-T SNP which is inconsistent with the graph.

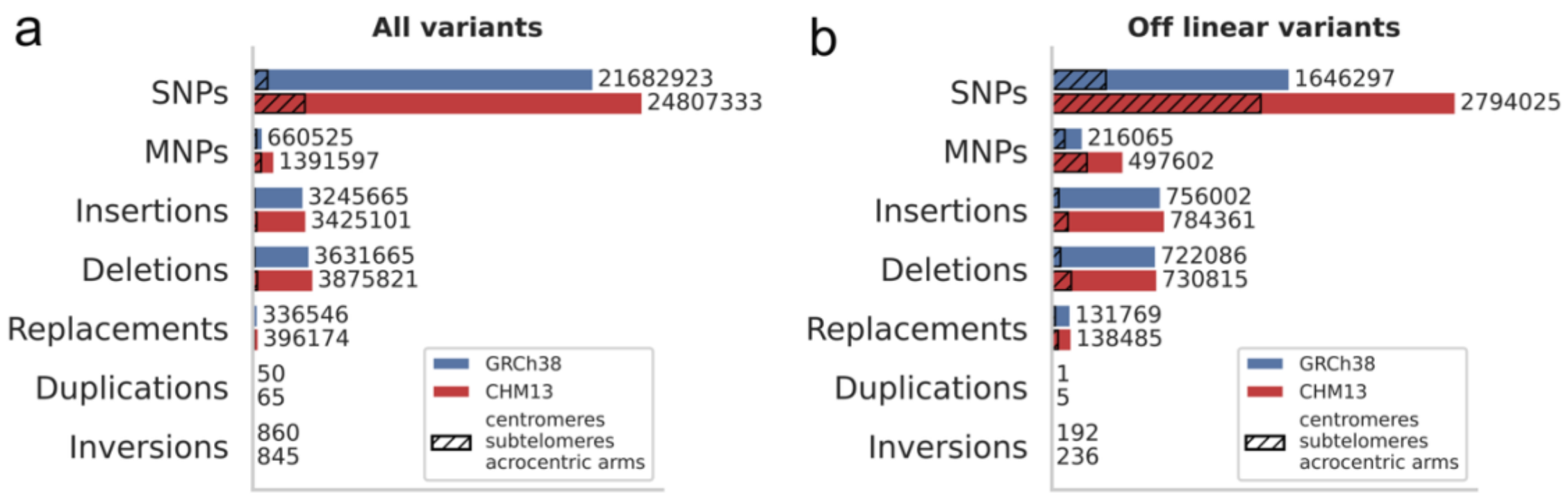

Figure S9. Variant comparison between the CHM13 and GRCh38 graphs, related to Figure 3. These graphs are based on the same data and the same algorithm, Minigraph-Cactus, but the algorithm is initialized at one or the other linear reference genome. The graphs differ in combined sequence length because regions missing from GRCh38 are excluded from the GRCh38 graph. These non-GRCh38 regions mostly include centromeres, subtelomeres, and acrocentric chromosome arms. (a) The number of variants detected by pantree in each graph. (b) The number of non-GRCh38 variants in the GRCh38 graph and the number of non-CHM13 variants in the CHM13 graph.

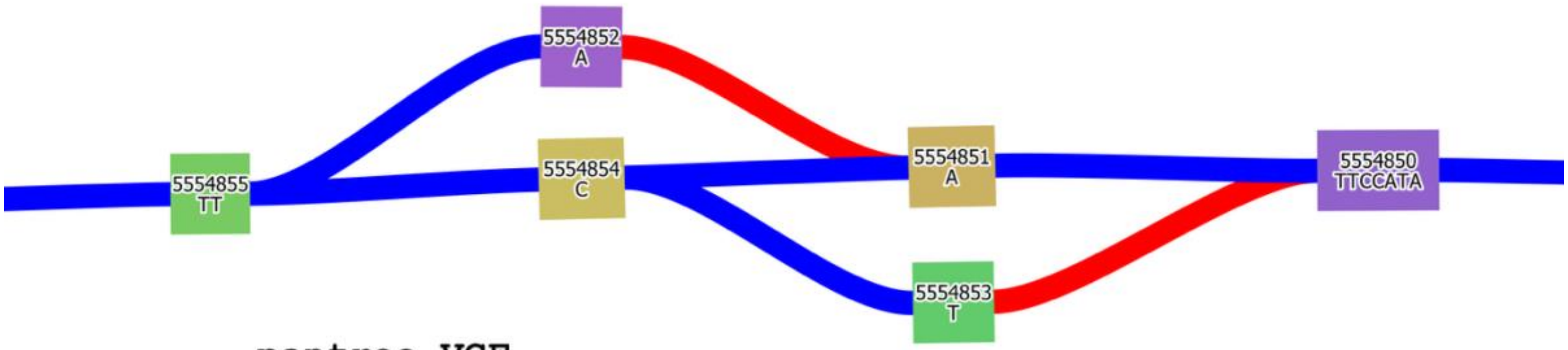

pantree VCF

```
CHROM POS       ID                   REF  ALT  INFO
chr1  125169613 >5554852>5554851     C    A    NR=.
chr1  125169614 >5554853>5554850     A    T    NR=.
```

superbubble VCF

```
chr1  125169613 >5554855>5554850     CA   CT
```

vcfwave

```
chr1  125169614 >5554855>5554850_1   A    T
```

Figure S10. SNP missed by vcfwave due to an assembly gap, related to Figure 4. pantree identifies two SNPs. One walk terminates at the node 5554851, and as a result, the ‘AA’ allele is not listed in the superbubble ‘raw’ VCF. As a result, the *vcfwave* VCF misses the ‘C’ to ‘A’ SNP.

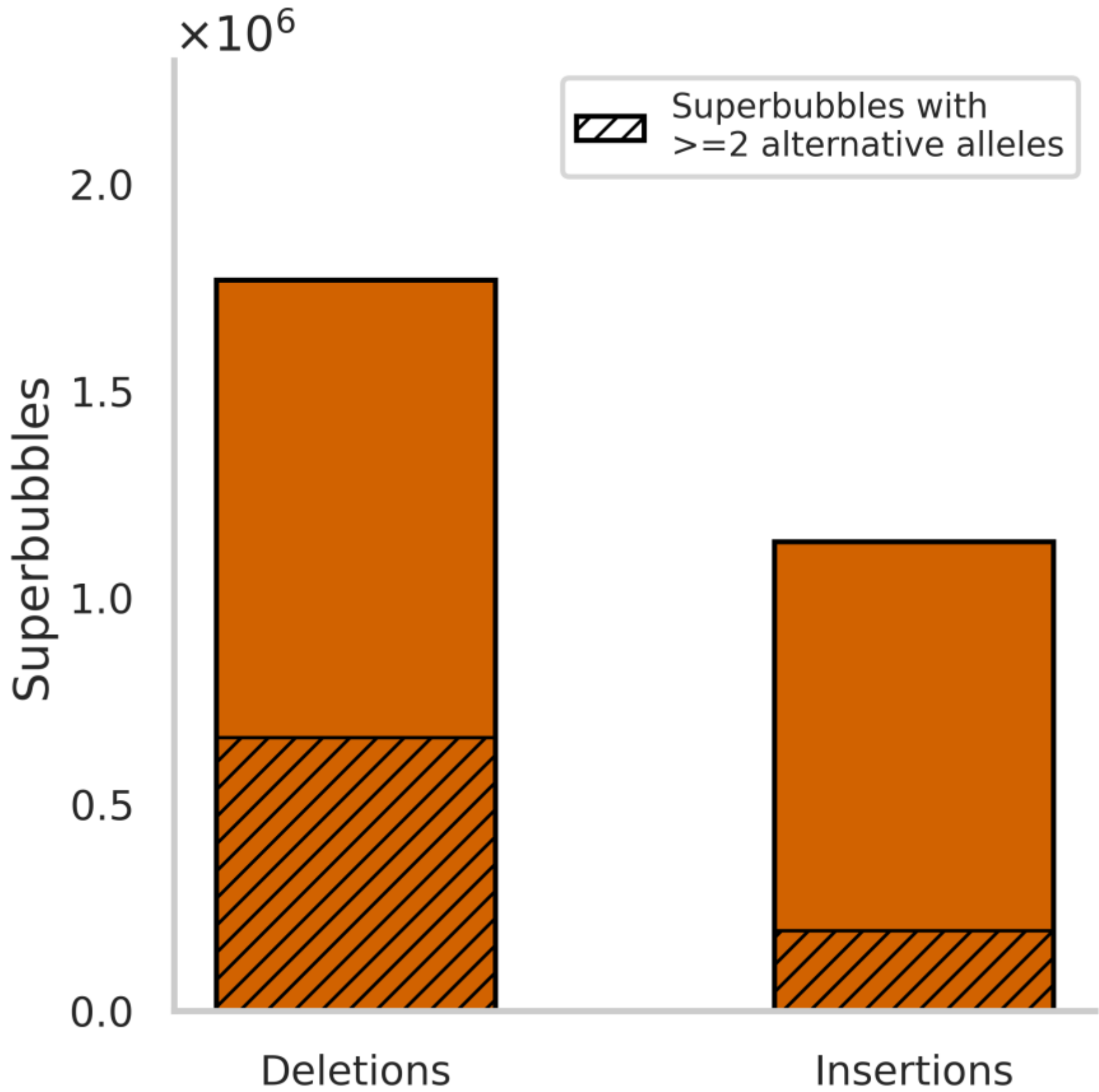

Figure S11. Proportion of insertion and deletion superbubbles, related to Figure 4. Among all superbubbles, there are 55.7% more deletion superbubbles (1,767,792) than insertion superbubbles (1,135,061). Among all multiallelic superbubbles, there are 241.6% more deletion superbubbles (661,196) than insertion superbubbles (193,553). Overall, 37.4% of deletion superbubbles are multiallelic, versus 17.1% of insertion superbubbles. This asymmetry results from the following ascertainment effect. Suppose that among all insertion or deletion superbubbles (biallelic or multiallelic), the average frequency of the empty deletion allele equals one half, such that GRCh38 has a 50% probability of having that allele. When restricting to multiallelic superbubbles, the average frequency should not equal one half, because a rare insertion is much less likely to be polymorphic compared with a common insertion (for example, a singleton insertion cannot be polymorphic). As a result, multiallelic superbubbles have a greater frequency of insertion alleles, and if GRCh38 carries a randomly sampled allele, then multiallelic indel superbubbles will usually be classified as deletions relative to GRCh38.

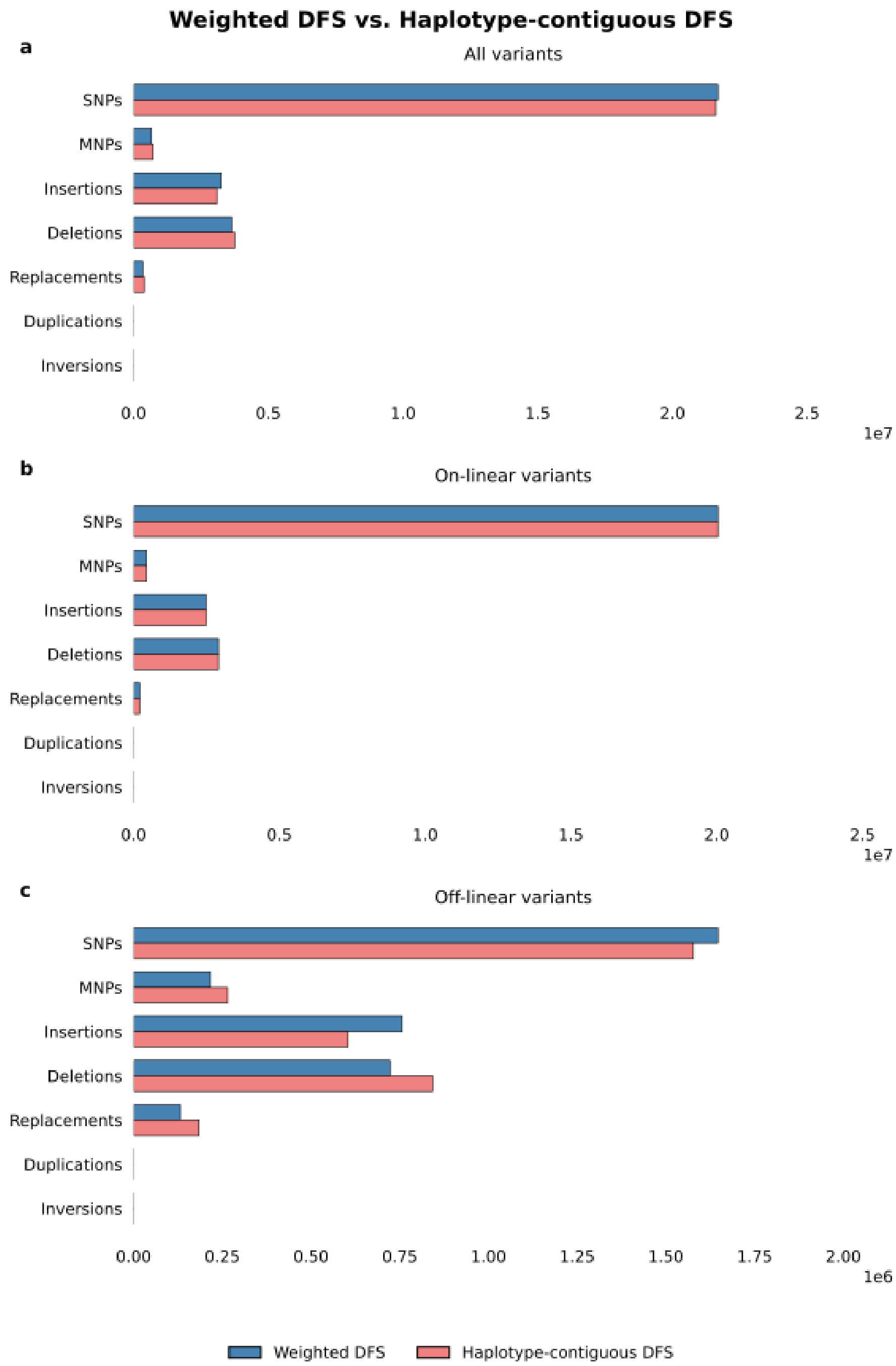

Figure S12. Comparison of variants defined by weighted DFS and haplotype-contiguous reference trees, related to Discussions. Variants were called on the same pangenome graph using two different reference-tree constructions: a weighted depth-first search (DFS), which prioritizes edges traversed by many haplotypes, and a haplotype-contiguous DFS, which prioritizes continuity along selected haplotypes. Panels a-c show counts for all variants, on-linear variants, and off-linear variants, respectively. Within each panel, bars for the two reference-tree choices are displayed side by side. Although individual variant edges differ between the two reference trees, the overall numbers of variants and the distribution of variant types are highly similar.

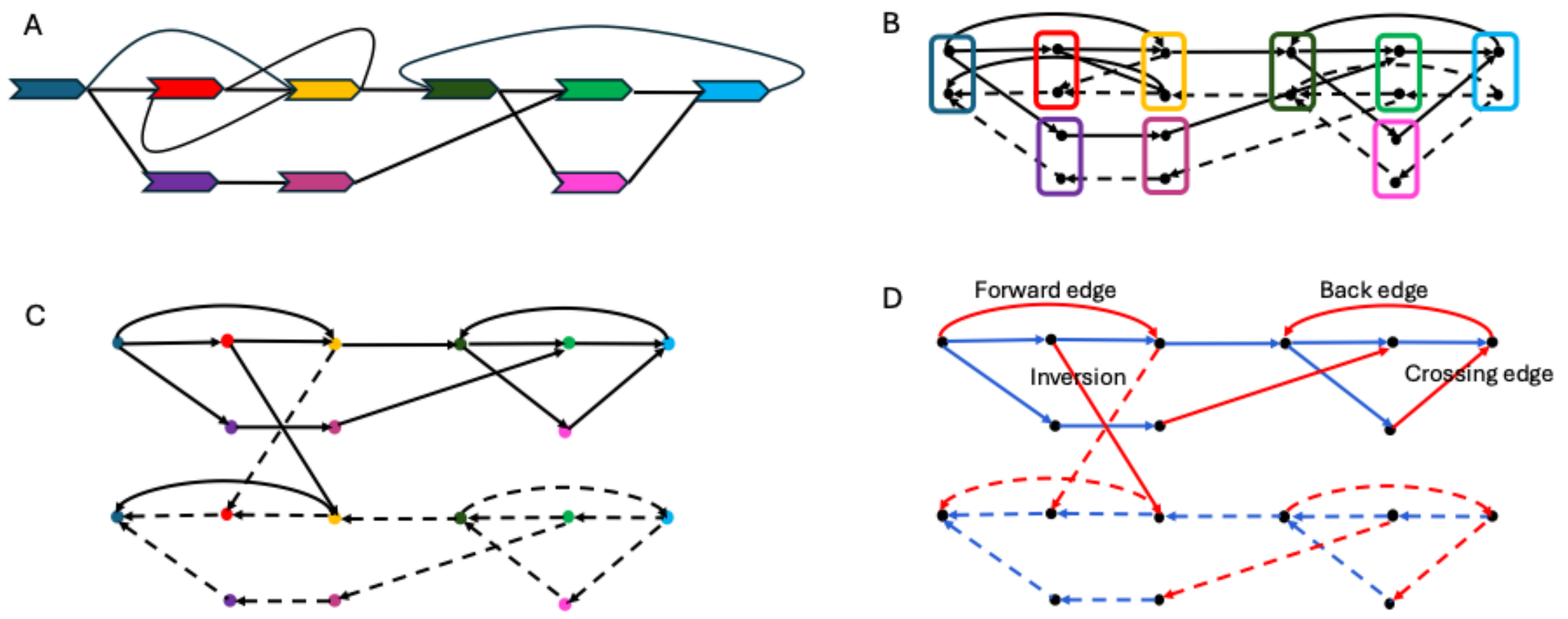

Figure S13. Bidirected and directed representations of a pangenome graph, related to STAR Methods. (A) Bidirected representation of a bidirected graph. Each binode is shown as a double-headed arrow. Biedges connect either heads or tails depending on traversal orientation. For example, the biedge between the purple and dark pink binodes connects the forward orientation of the purple binode to the forward orientation of the dark pink binode, and the reverse orientation of the dark pink binode to the reverse orientation of the purple binode. (B) Directed representation of the same graph, where each binode is unfolded into two complementary nodes, one representing forward orientation and one representing reverse orientation. The color scheme follows panel (A). Each biedge corresponds to a pair of directed edges—one shown solid, one dashed. (C) An expanded version of (B), where complementary nodes are spatially separated for clarity. (D) The reference tree highlighted in blue. The tree over forward oriented nodes forms a DFS tree. Variant edges (in red) are classified relative to this tree as forward, back, or crossing edges. Inversion edges connect a forward oriented node to a backward oriented node.

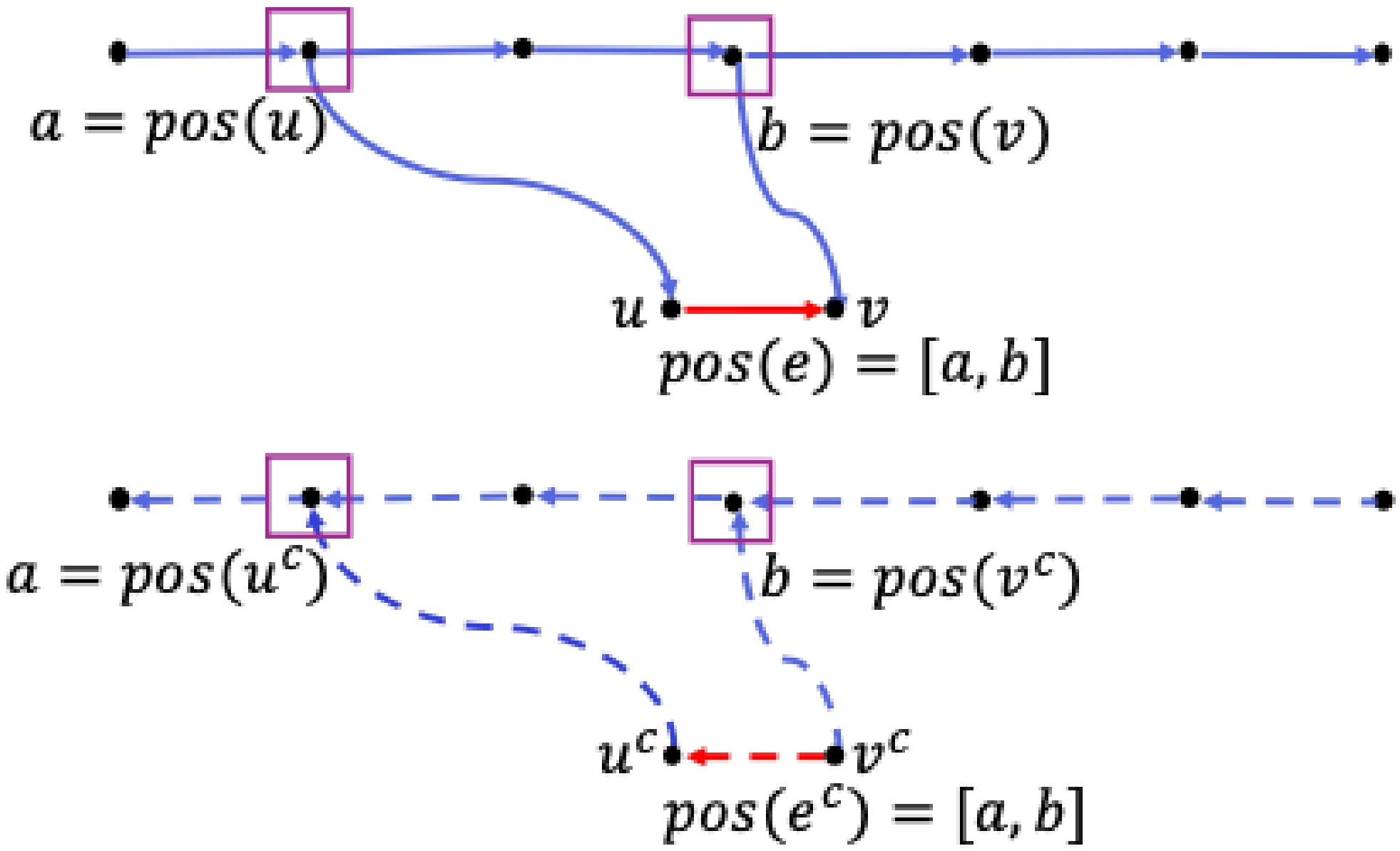

Figure S14. Position of a variant edge and its complement, related to STAR Methods. The blue paths represent the linear reference in forward (solid) and reverse (dashed) orientations. The position of a node v is defined as the position of its lowest ancestor in the forward-oriented linear reference (or its first descendant in the backward-oriented reference). For a variant edge $e = (u, v)$, the position is defined as the interval pos(e) = [pos(u),pos(v)]. The position of a node is the same as that of its complement, and similarly, the position of a variant edge is equal to the position of its complementary edge.

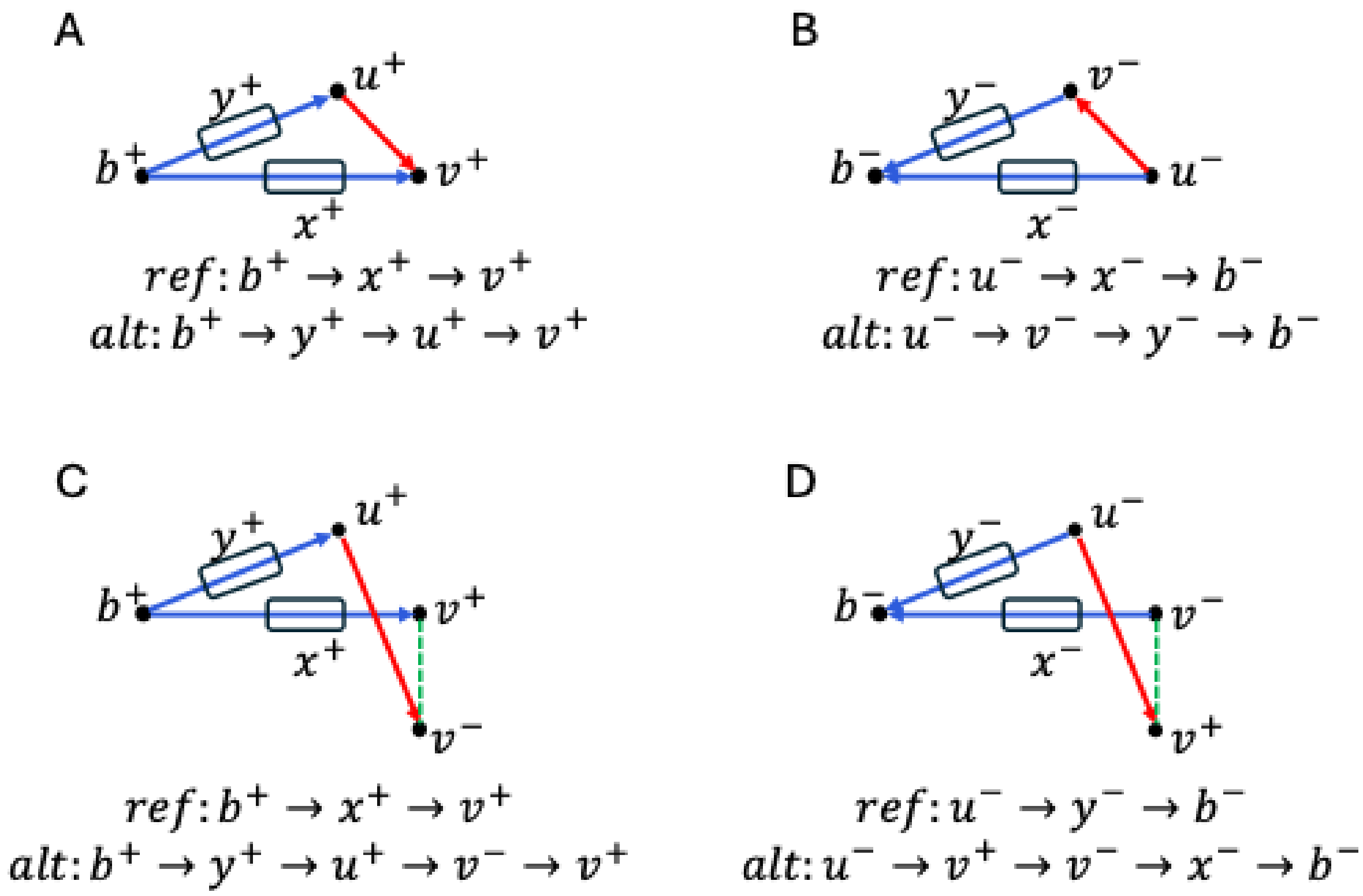

Figure S15. Reference and alternative paths for all orientations of a variant edge, related to STAR Methods. Each panel shows the reference path and the alternative path for a variant edge (red). Edges in the reference tree are shown in blue. Panels (A) and (B) illustrate cases where both endpoints of the variant edge lie in the same orientation: (A) forward ($u^+ \to v^+$) and (B) reverse ($u^- \to v^-$). In these cases, the branch point and both endpoints of the variant edge lie within the same half-tree. The two internally disjoint paths from the branch point to the variant endpoint form the reference and alternative paths, distinguished by whether or not they include the variant edge. Panels (C) and (D) illustrate inversion edges: (C) forward to reverse ($u^+ \to v^-$), and (D) reverse to forward ($u^- \to v^+$). In these cases, the second node of the variant edge lies in the opposite half-tree from the branch point and the first node. The green dashed undirected edge represents a switch within the binode that allows traversal between orientations. Reference and alternative paths remain a pair of internally disjoint parallel paths; the alternative path traverses both the variant edge and the green switch edge.

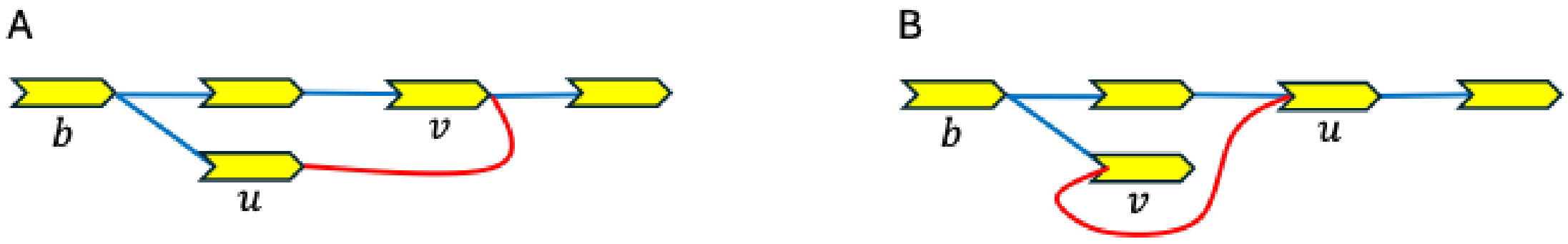

Figure S16. Illustration of allele simplification when a path visits both orientations of the same binode, related to STAR Methods. In both panels, the reference path (blue) follows the linear reference sequence. (A) The alternative path traverses nodes v and u^c but does not include both u and u^c or both v and v^c consecutively, so no cancellation occurs, and the alternative allele includes the sequence of u^c. This is visually clear from the figure since the alternative path enters and exits from different sides of binodes u and v when traversing the variant edge. (B) The alternative path contains both u, u^c or both v, v^c, and this results in a simplified allele with the corresponding segment omitted. This is visually clear since the alternative path enters and exits the same binode from the same side (either $u \rightarrow u^c$ or $v \rightarrow v^c$ depending on the orientation of the traversal).
