# Supplementary material for: Defining and cataloging variants in pangenome graphs
Source: Cell Genom. 2026 Aug 17;6(9):101327. doi: 10.1016/j.xgen.2026.101327 (PMC13576716; doi:10.1016/j.xgen.2026.101327)
Supplement: Supplementary file 2 [file mmc21.pdf]

Cell Genomics

Transparent Peer Review Record

Summary

Initial submission:	Received : 9/6/25
	Scientific editors: Sara Rohban and Laura Zahn
First round of review:	Number of reviewers: 3<br>Revision invited : 11/3/25<br>Revision received : 3/37/26
Second round of review:	Number of reviewers: 2
Third round of review:	Number of reviewers: 1<br>Accepted : 7/14/26
Data freely available:	Yes
Code freely available:	Yes

This transparent peer review record is not systematically proofread, type-set, or edited. Special characters, formatting, and equations may fail to render properly. Standard procedural text within the editor's letters has been deleted for the sake of brevity, but all official correspondence specific to the manuscript has been preserved.

Referees' reports, first round of review

Reviewer #1: The authors present a novel approach called pantree to define and call variants represented in a pangenome graph. Their method defines a reference tree structure and variant edges in order to identify variants in nested bubble structures of the graph. Compared to existing approaches, pantree can also call variant alleles relative to non-reference sequences. The authors apply their method to pangenome graphs generated by the HPRC.
Overall, I think the method is very well designed and theoretically sound. However, to fit into the scope of the journal, the paper misses some real world use cases that demonstrate how it can be helpful to gain new biological insights.

Major Comments:

There exist several other approaches to decompose multi-allelic bubbles in a pangenome graph. Besides the vcfwave approach mentioned in this paper, Liao et al. introduce two other traversal-based approaches in their paper (https://github.com/wwliao/hpp_freeze1_pangenome_graph_qc/tree/main/decomposition and https://github.com/eblerjana/genotyping-pipelines/tree/main/prepare-vcf-MC) which call nested variants from multi-allelic bubbles based on analyzing path traversals of haplotypes through superbubbles. None of them seems to call variants within non-reference sequence though. I think a comparison to these additional methods is not necessary, but they should be mentioned in the text.

The authors should also compare their non-SNP variant calls to calls reported by vcfwave. So far, it seems that the comparison is based on SNPs only. While comparing indels or SVs is more difficult, there are tools available like hap.py, vcfeval or truvari that might be helpful here. In addition to this, the authors should compare their variant calls to external ground truth callsets to demonstrate that their approach produces correct results for reference-based variants. Options might be GIAB small and SV callsets or assembly-based callsets generated by tools like PAV (i.e. such produced by the HGSVC).

For non-reference variants, did you do any kind of evaluation to confirm that they are real? Since multi-allelic bubbles can often be very large and complex, might some of these variants also be artifacts?

While I think that the method is well defined on the theoretical site, the paper does not provide any actual use case to show that the novelty of the method, which is to detect variation alleles nested inside of non-reference sequence, is useful in practice to gain biological insights. Currently, the authors only report statistics on the number of (non-reference) variants detected in HLA and RHD regions, but no further analysis is conducted on these variants to verify that they are real or to analyze their biological effects.

I agree that defining a coordinate system for non-reference variants is tricky, so I'm wondering how one would work with these variants in practice (also related to my previous comment). More specifically, how would one analyze such non-reference variants in genomes not contained in the graph if there is no common coordinate system? In other words, how could one check if a newly assembled genome carries a particular non-reference variant? Another question that arises is how to compare these non-reference variants across different pangenome graphs, i.e. would it be possible to check if certain non-reference variants found in one graph are represented in the other one? So I think the authors should propose some solutions to these questions.

I really like the graph examples shown in Figure 3 i+j as I think they nicely demonstrate the advantage of pantree over existing methods. However, I found the VCF records shown in Figure 3j for the superbubble VCF and the vcfwave VCF confusing: the number of alternative alleles in the superbubble VCF and in the vcfwave VCF actually depends on the haplotype paths represented in the graph, as there will be one allele for each distinct path covered by the haplotypes passing through the bubble. In the Figure, two alleles are listed for the superbubble, one which seems to follow the reference path (all blue edges) and the other one passing through

all variant edges. However, in reality the superbubble and vcfwave VCFs might contain many more alternative alleles. So I think it would be useful to show haplotype paths traversing this bubble in the figure, otherwise it is unclear why there are exactly two alternative alleles listed in the superbubble / vcfwave VCFs. Also two minor points regarding the same Figure: why is the reference allele (REF) not listed for the superbubble and vcfwave VCFs? And why is the second ALT sequence of the superbubble VCF one base shorter than the other one?

Minor Comments:

The github link: https://github.com/ShenghanZhang1123/graph_var_analysis is not accessible.

The version of all software tools used in the analyses described should be added.

Reviewer #2: The authors describe a novel way to define and catalog variants in pangenome graphs that avoids the problems in current bubble-based approaches. Data and code are both available. Most of my comments concern parts of the text where I would appreciate more detail/elaboration to make it easier to understand.

In Supplementary Note, Section 8, a toy example for each variant edge category would be helpful to the readers.

"The larger number of deletions is mostly due to an ascertainment effect, that deletions are more likely to be multiallelic." Can the authors explain why this occurs more often with deletions than with insertions?

"This approach relies on our variant definition because there is no similar way to simplify the graph without first discarding unwanted edges." Can the authors elaborate? Which approach? Pruning + tip deletion + contraction? Or perhaps the authors meant "This approach is necessary due to our variant definition…"? How do you define unwanted edges?

Can the authors release a standalone script/pipeline to run the techniques applied to simplify the graphs and obtain the simplified graphs in Figure 4? Or add an optimal parameter in pantree to emit the simplified graph in GFA format?

How to compare variants made with different pangenome trees of the same pangenome graph? Is there a way to 'normalize' the representation to represent the same variant in the same way?

Is there an optimal way to choose the reference tree (for example, to minimize the amount of variation output), or are all possible reference trees equivalent? I see authors defined a heuristic to maximize the allele frequency of reference alleles greedily. How does the heuristic impact the final output? What other heuristics would be worth exploring?

Authors prefer to define variant positions against an acyclic reference genome path, thus ruling out working with the type of graphs created with methods like PGGB. Is there a way to extend pantree to work with PGGB graphs, or edit PGGB graphs to allow users to use pantree on them?

Reviewer #3:
This paper proposes a novel method to identify and classify genomic variants in a pangenome graph. This a timely and well needed methodological contribution since pangenome graphs are increasingly built and used for various organisms. There are very few existing approaches for identifying variants in such graphs and they suffer for various drawbacks. In particular they are biased/limited by the use of a reference genome, some variants are missed or redundantly represented and they are not classified by types.

The authors introduce a novel data structure, the reference tree, that has the potential to remove these limitations and is an original and very promising way of addressing this problem. The method is implemented in an open source tool called pantree available on github. The authors

apply their method on the human draft pangenome graph built with Minigraph-Cactus and characterized the variant set they obtained, and compared it to another existing approach (superbubbles).

The main contribution of this paper lies on the methodological side. The novel method proposed is a very important and valuable contribution that will certainly influence future methodological work on pangenome graphs as well as how we study variants in such graphs. However, the presentation of the method lacks clarity and the experiments do not allow a proper validation of the implementation nor a fair comparison with existing approaches.

1. Major concerns with the presentation of the method:

- The description of the method in the Result section of the main manuscript is not enough formal and lacks some explanation to understand the concept and intuition behind the method. On the contrary, the description of the method in Supplementary Material is much more technical and formal, which is a good thing, but it may be difficult to read for non computer-science or graph theory readers. A description of intermediate precision is missing from the main text. For instance, how the reference tree is built is not explained in the main text, nor the important role of the reference genome in its construction. The full pipeline of the implemented method, showing the successive steps of computation, is not described anywhere and would probably help the reader. An algorithm of the heuristic implemented for chosing the edges of the spanning tree would also be helpful. Another suggestion would be to describe the method more formally in the main text but without the bi-directionality aspects...

- Figure 1 could be improved by defining and showing the variants represented in the toy graph and their types. I appreciated the examples of representation of variants as superbubbles and in the pantree VCF in Figure 3 and Suppl Figs 8 and 10, such examples could be shown earlier in the main text.

- Similarly, as a major feature of the approach is to catalogue variants in different types, a figure showing the different types of variants and their associated properties of variant edges would improve the clarity.

2. Major concerns with the empirical validation of the method and comparison to superbubbles:

- The comparison of obtained variants with pantree is restricted to superbubbles of level 0. Fist, this should be clearly stated in the Result section, not just in the Method section, since it explains why pantree output much more SNPs. Secondly, I find it too restrictive. The authors should compare their variants to higher levels of superbubbles. The authors justify this choice by declaring that 1) "higher-level bubbles do not yield variant definitions with well defined positions along the linear reference genome" and 2) "these bubbles introduce redundancy: the same variant may appear in multiple bubbles across different levels, each offering a different representation". I do not agree with these statements: if the authors restrict their comparison to SNPs (as it is the case in presented results) and to variants involving the reference genome, variant positions are well defined (as output by vg-deconstruct in a VCF format positioned on the reference genome) and without any redundancy. With such a comparison, we could better interpret the differences in number of SNPs: this will validate that pantree not only does not miss any simple SNPs but also no nested ones. This will better highlight which SNPs are missed by the superbubble approach (non-reference SNPs and those in interlocking and overlapping bubbles), compared to the vcfwave comparison which gives false negatives (SNPs not found by pantree) due to re-alignment artefacts (as stated by the authors).

- Similarly, the comparison is restricted to SNPs, and disregards other variant types such as small indels or larger SVs, which are harder to find and characterize in pangenome graphs and where the benefits of pantree are probably greater. Here again, by looking at all levels of superbubbles but restricting to variants involving only a pair of genomes (including the reference genome), variant positions and alleles should be well defined and comparable between the two approaches. This would consolidate the validation of the implementation of the method for all variant types, and probably show if the benefits are of the same order of magnitude for all variant types.

- The superbubble approach implemented in vg-deconstruct can output non-reference variants by changing the path of the reference given as a parameter. I agree this is not convenient, nor efficient to re-run vg-deconstruct for all possible reference paths. However, could the authors use this approach to validate or compare at least a subset of the non-reference variants obtained with pantree ?

- The authors claim that their approach can be applied to other graphs than those built with Minigraph-Cactus, such as those obtained with PGGB. They should show some empirical results on such graphs.

- As the main contribution of this paper is an implemented method, which is a meaningful alternative to the superbubble approach (such as implemented in vg-deconstruct), it would be interested to show some performance results: how does it compare to vg-deconstruct in terms of running times and memory consumption ?

3. Major concerns with the introduction:

- Some references to other methods to find variants in pangenome graphs are missing in the introduction: vg-deconstruct, gfatools bubble, Bubble Gun.

- I do not agree with the statement "a variant is a superbubble", the authors should talk about the hierarchy of bubbles, not all bubbles in the hierarchy require that source and sink nodes are traversed by all haplotypes, and this allows to represent more finely small nested variants.

- The limitations of bubbles and their hierarchy should be explained in a more rigorous way. Giving precise examples of interlocking and redundant variants with bubbles, together with their representation with pantree could highlight further the benefits of the proposed method. I appreciated the examples in fig. 3 and for instance supp figures 10, but they seem to be rather particular examples and arrive late in the reading.

- "This definition satisfied our intuition": which one ?

- "it has the right mathematical properties": no mathematical properties have been stated before

4. Major concerns with the software:

- I successfully installed the software, but it was not straightforward as the Readme lacks instructions for installion.

- I successfully run the software on the provide toy examples but it was more complicated on my own pangenome graphs as information about the desired gfa format of input file was missing in the Readme. For instance, the gfa should contain W lines and not P lines, L lines should be placed before W lines in the file (which was not the case for my graph obtained with Minigraph-Cactus)...

- the authors present in the paper a tool to simplify the graph, however there is no executable for it in the repository (nor documentation in the Readme). It could be useful to provide such a tool based on the reference tree with a gfa output.

- I could not access the scripts to reproduce the analyses, stated to be available à this github repository, which is not a valid link : https://github.com/ShenghanZhang1123/graph_var_analysis

5. Minor comments:
- Results on complex regions: it seems to be the same regions analyzed in details in the draft human pangenome paper (Liao et al 2023), could the authors compare the findings and biological interpretations between both analyses ?
- supp fig. 8: why was vcfwave applied on this superbubble, as it is not multi-allelic ?
- p.6 and 7 of suppl. material, L is not defined, there seems to be 2 proofs for Theorem 4.1 ;

- p.10 of suppl. material sup Fig 3, what are x and y ?
- p.11 of suppl. material, sup. fig. 4, it would help understand the figure if the paths on the pangenome graphs were shown. The legend contains typos: "traversin" "and This".

Authors' response to the first round of review

We thank the editor and reviewers for their thoughtful comments. We would like to clarify the main contributions of our manuscript, of which there are two. First, we introduce a new definition for what "genetic variant" means in a pangenome graph. This contribution is fundamental. It enables the study and association mapping of non-reference variants which are invisible to all genetic studies today. None of the reviewers have raised concerns about our definition. Second, we implement a method to identify variants under our definition, we provide a catalog of variants, and we perform a series of methods comparisons and validation analyses. This contribution strengthens the significance of our first contribution by demonstrating that no previous definition or method is similar to ours. However, because these comparisons occupy a large fraction of the main text, it is possible to lose sight of our main contribution. Our response to reviewers focuses on strengthening our second contribution, but we wish to emphasize the greater importance of our first contribution, which is the novel definition itself. Moreover, there exist many open questions and methodological gaps in pangenome analysis which are not addressed by our paper; some of these gaps have been pointed out by the reviewers, and in such cases we have tried to explain why filling these gaps is out of scope for our manuscript.

REVIEWER #1

1.0. Original comment: The authors present a novel approach called pantree to define and call variants represented in a pangenome graph. Their method defines a reference tree structure and variant edges in order to identify variants in nested bubble structures of the graph. Compared to existing approaches, pantree can also call variant alleles relative to non-reference sequences. The authors apply their method to pangenome graphs generated by the HPRC. Overall, I think the method is very well designed and theoretically sound.

Response: We thank the reviewer for their positive assessment and for their detailed review.

1.1. Original comment: However, to fit into the scope of the journal, the paper misses some real world use cases that demonstrate how it can be helpful to gain new biological insights.

Response: Thank you for this comment. As described in our response to comment 1.5, we revised the Introduction and Discussion to better contextualize the contribution of this work and to clarify that the present manuscript focuses on defining and systematically cataloguing variants in the HPRC draft pangenome graph, rather than on comprehensive functional interpretation of individual variants.

Major Comments

1.2. Original comment: There exist several other approaches to decompose multi-allelic bubbles in a pangenome graph. Besides the vcfwave approach mentioned in this paper, Liao et al. introduce two other traversal-based approaches in their paper (https://github.com/wwliao/hpp_freeze1_pangenome_graph_qc/tree/main/decomposition and https://github.com/eblerjana/genotyping-pipelines/tree/main/prepare-vcf-MC) which call nested variants from multi-allelic bubbles based on analyzing path traversals of haplotypes through superbubbles. None of them seems to call variants within non-reference sequence though. I think a comparison to these additional methods is not necessary, but they should be mentioned in the text.

Response: We thank the reviewer for pointing out these additional traversal-based decomposition approaches. We have now added explicit discussion of them in the Introduction. The revised text reads:

Currently, variants in pangenome graphs are defined as different kinds of "bubbles": loosely, regions where two walks split apart and then merge back together (Onodera et al., 2013; Paten

et al., 2018). In a directed graph, a superbubble is such a region with a single entry node and a single exit node, such that every directed path from the entry to the exit is contained within the

bubble. The bidirected generalization of a superbubble, allowing for inversions, is called a snarl (Paten et al., 2018). Snarls can be organized into a nested hierarchy. However, a challenge is that snarls can contain many long alleles, some of them differing by only a few nucleotides despite being kilobases in length. In such regions, hierarchical decomposition succeeds for properly nested events, such as a long insertion containing several SNPs, but it cannot decompose interlocking structures, and nested sites do not in general inherit unique coordinates on the chosen linear reference path (Liao et al., 2023; Paten et al., 2018).

Liao et al. used three methods to work around these limitations. First, vg deconstruct was used to identify snarls and emit a raw VCF for level-0 snarls as well as nested snarls traversed by the reference allele (for example, SNPs nested inside of deletions). Second, they introduced the vcfwave pipeline, which takes the allele sequences from the raw VCF records emitted by vg deconstruct, realigns them to the linear reference, and decomposes the resulting alignments into "primitive" variants. This approach recovers small variants nested within deletions and other replacement-like alleles, but not within insertions, because the reference allele is still required to lie on the linear reference genome. Third, Liao et al. also implemented a traversal-based decomposition procedure (distributed as the resolve-nested-genotypes tool) that uses the alleletraversal annotations in the raw VCF: for each raw VCF site, it compares the graph traversal of the reference allele to the traversals of the alternative alleles and extracts the implied nested SNPs and indels as separate biallelic variant records. Unlike vcfwave, which decomposes variants by realigning allele sequences back to the linear reference, this third approach decomposes them by analyzing how haplotype paths traverse the graph site itself.

Beyond the snarl/superbubble framework used in HPRC, several related bubble-like site definitions and detection methods have been proposed. In the bidirected framework of (Paten et al., 2018), ultrabubbles are a special class of snarls whose interior is acyclic and tip-free. More recently, Billi introduced panbubbles as a bidirected generalization of superbubbles and also uses bibubbles as a second site class for more complex configurations (Bhat et al., 2025). In a different setting, pangene uses bibubbles in gene graphs to represent gene-content differences rather than sequence-level allelic variation (Li et al., 2024). The corresponding tools relate to these definitions in different ways: vg detects snarls (Garrison et al., 2018; Paten et al., 2018), BubbleFinder computes several related bubble-like structures, gfatools bubble detects bubblelike structures in pangenome graphs (Li et al., 2020), Billi detects panbubbles and bibubbles, and pangene detects bibubbles in gene graphs (Li et al., 2024). BubbleGun similarly detects bubble-like structures and related bubble chains in sequence graphs (Dabbaghie et al., 2022). Although these methods differ in their formal site definitions, they share a common limitation for variant representation: they identify local bubble-like subgraphs but do not by themselves provide a coordinate system along a chosen reference path, and therefore cannot directly produce a non-redundant, genome-wide biallelic VCF representation of all variants.

These additions now explicitly acknowledge the traversal-based approaches noted by the reviewer and clarify how they differ from pantree. In particular, they remain tied to linearreference-based output and therefore do not define alleles inside non-reference sequence.

1.3. Original comment: The authors should also compare their non-SNP variant calls to calls reported by vcfwave. So far, it seems that the comparison is based on SNPs only. While comparing indels or SVs is more difficult, there are tools available like hap.py, vcfeval or truvari that might be helpful here. In addition to this, the authors should compare their variant calls to external ground truth callsets to demonstrate that their approach produces correct results for reference-based variants. Options might be GIAB small and SV callsets or assembly-based callsets generated by tools like PAV (i.e. such produced by the HGSVC).

Response: We thank the reviewer for this suggestion. In the revised manuscript, we added two

new validation analyses. First, we benchmarked small variants against an external truth set using the small variant calls of HG002 from the GIAB Q100 v1.1 dataset as ground truth and evaluated graph-based callsets with RTG vcfeval. We report false-negative and false-positive counts for SNPs and INDELs for four VCFs: raw graph-based callset, vcfwave, pantree, and a postprocessed “pantree-pairwise” callset. Pantree-pairwise performed best overall in this truthset evaluation, particularly for indels; this method postprocesses the pantree VCF to align its variant representation with what is expected by the benchmark, namely a pairwise comparison between two haplotypes instead of an all-versus-all comparison of several haplotypes. This analysis is detailed in the Results section with a new main figure panel (Figure 3c, reproduced below), and we also added a corresponding Methods subsection, “Assembly-based validation.”

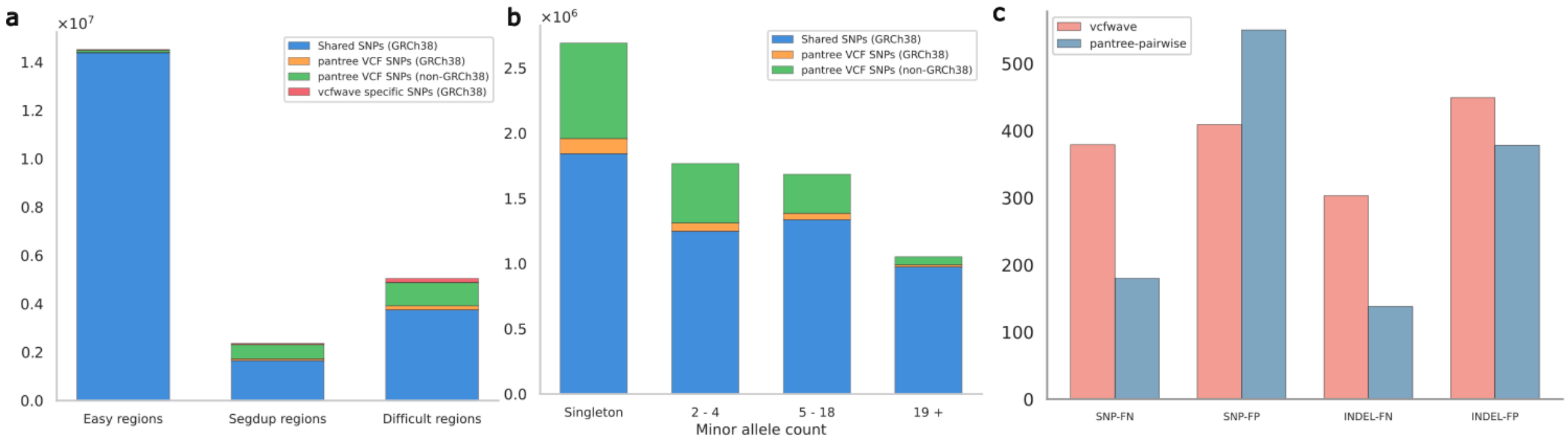

c) Small-variant benchmarking against an external truth set using RTG vcfeval, showing SNP and indel false negatives (FN) and false positives (FP) for vcfwave and the pantree-pairwise postprocessed callset.

Second, we extended the comparison beyond SNPs by benchmarking non-SNP variants between the raw HPRC chr20 VCF and the pantree VCF. We describe this analysis in the Results section: “For biallelic variants, we required an exact match of chromosome, position, REF, and ALT between the raw and pantree VCFs. Under this strict criterion, pantree recovered all biallelic insertions (1,600/1,600), all biallelic deletions (1,826/1,826), and 84.9% of biallelic ‘other’ variants (399/470).” We also explain why one-to-one matching is inherently ambiguous for multiallelic sites, where pantree and bubble-based approaches represent the same local structure differently. The benchmarking procedure is described in the Methods subsection “non-SNP benchmarking against the raw VCF.” Together, these additions address the reviewer’s request for both truth-set-based validation and a non-SNP comparison.

1.4. Original comment: For non-reference variants, did you do any kind of evaluation to confirm that they are real? Since multi-allelic bubbles can often be very large and complex, might some of these variants also be artifacts?

Alternative Response: Answering what variants are real depends on the definition of “real”. Pangenome graphs are constructed from stringent multiple sequence alignment (MSA). One possible definition is that a variant is “real” if it corresponds to a genuine difference between two assemblies in the MSA. By this definition, pantree’s variant edges are guaranteed mathematically to be real. However, the MSA encoded by the pangenome graph itself could in principle be “wrong”, such that a variant is “real” with respect to the graph but not with respect to some “true” MSA. The problem with this is that in complex regions, we do not know how to define the “true” MSA.

In particular, complex regions blur the distinction between orthology and paralogy. A variant in a simple unique region intuitively refers to a difference between orthologous sequences; such variants have the interpretation that they correspond to mutation events. This definition is not easily generalizable. Orthology is defined through evolution, but we do not have a method to

trace the evolution of complex multiallelic bubbles in the presence of frequent non-allelic gene conversions that mixed orthologous and paralogous sequence copies. For example, at an STR locus, recurrent mutation makes it impossible to reconstruct which repeat elements are homologous between two haplotypes. Sequences in the MSA are related in evolution; we just could not tell orthology from paralogy. Without a practical definition, we cannot test what variants are real based on orthology.

In our work, "variants" are effectively differences between human genomes as is encoded in the input graph. They may arise from orthlology, paralogy or a mixture of both, but regardless of the origin, such variants are real differences between assemblies and can inform association with phenotypes. With well-defined coordinates, pantree provides a unique opportunity to study the association of non-reference variants in the long run.

We added the following paragraph to the Discussion:

pantree suffers from two potential sources of error, both related to its input data. First, although the HPRC assemblies have undergone extensive validation, residual assembly errors would produce spurious variant edges. Second, Minigraph-Cactus (Hickey et al., 2024) can make alignment errors, especially in tandem repeats and segmental duplications. At short tandem repeat loci, recurrent mutation makes it impossible to reconstruct a unique homology structure between repeat copies, so some aligned sequences may be only approximately homologous. In such regions, pantree variants remain a valid and useful representation of the differences between haplotypes as encoded by the graph, but they should not be interpreted as a record of individual mutation events in the genealogical history of orthologous sequences.

1.5. Original comment: While I think that the method is well defined on the theoretical site, the paper does not provide any actual use case to show that the novelty of the method, which is to detect variation alleles nested inside of non-reference sequence, is useful in practice to gain biological insights. Currently, the authors only report statistics on the number of (non-reference) variants detected in HLA and RHD regions, but no further analysis is conducted on these variants to verify that they are real or to analyze their biological effects.

Response: Regarding the validity of our results in these regions, the HPRC paper [PMID: 37165242] uses both HLA and RHD as examples (Fig. 5 in the HPRC paper). Around HLA-A, the HLA-Y insertion and the long deletion involving four pseudogenes have long been documented. RHD involves novel alleles at the time which HPRC has compared to the literature and provided detailed analysis. The structural variants at these loci are real.

Regarding biological insights, this question is out of scope for our manuscript. Establishing biological effects is inherently difficult, especially in a pangenome setting where functional annotation and association frameworks differ from linear-reference analyses. For this reason, historically, expanding the variant catalog has preceded biological insight. It is the premise of defining a pangenome reference that non-linear reference sequences are biologically important yet undercharacterized; defining genetic variants inside these regions is a significant step toward addressing this challenge, but it does not solve the problem of functional annotation (for example, non-reference sequences cannot be inserted into tools like VEP) or association mapping (this would require a method to call non-GRCh38 variants in an external dataset with paired phenotype data). In our last Results subsection, we already show how pantree finds novel variants in biomedically important regions harboring complex structural variation. However, a full functional analysis is beyond the scope of this manuscript; our contribution is to define and catalog variants, enabling subsequent work to assess biological impact. To make this point explicit, we added the following paragraph to the Discussion:

The functional consequences of the genetic variants that we found are unknown. Discovering variants is a prerequisite for characterizing their effects, and historically, these efforts have been separate stages; for example, the International HapMap Project first built a public map of common human variation and then enabled downstream association studies using that resource. At a minimum, we do know that complex loci can be medically relevant, and pantree

identifies novel variation at such loci (HLA-A and RHD). Understanding the effects of such variants, at these and other loci, will require experimental evidence and possibly genetic association studies.

1.6. Original comment: I agree that defining a coordinate system for non-reference variants is tricky, so I'm wondering how one would work with these variants in practice (also related to my previous comment). More specifically, how would one analyze such non-reference variants in genomes not contained in the graph if there is no common coordinate system? In other words, how could one check if a newly assembled genome carries a particular non-reference variant? Another question that arises is how to compare these non-reference variants across different pangenome graphs, i.e. would it be possible to check if certain non-reference variants found in one graph are represented in the other one? So I think the authors should propose some solutions to these questions.

Response: The reviewer is describing a genotyping problem which is separated from variant representation. In principle, we could align a new assembly to the existing graph and report if a non-reference (or reference) variant edge is present on an alignment walk. This would address the reviewer's question. In practice, no tools can align an assembly to a minigraph-cactus graph, but this is not a limitation of our pantree method.

For different graphs built from the same haplotypes, it is possible to check whether a variant is present in both graphs using the following procedure: (1) pick a variant in the first graph; (2) choose a haplotype carrying the reference allele and record the position of that allele on that haplotype; (3) locate the same haplotype and position in the second graph; (4) check whether, in the second graph, that haplotype carries the reference allele at that position of a variant edge with the same alternative allele as well. This procedure checks whether the same variant is present in both graphs with identical representations but will miss cases that the same variant is represented differently. In our revised manuscript, we pose this question in the Discussion and propose this procedure together with its limitations in the Supplementary Note section 13.

1.7. Original comment: I really like the graph examples shown in Figure 3 i+j as I think they nicely demonstrate the advantage of pantree over existing methods. However, I found the VCF records shown in Figure 3j for the superbubble VCF and the vcfwave VCF confusing: the number of alternative alleles in the superbubble VCF and in the vcfwave VCF actually depends on the haplotype paths represented in the graph, as there will be one allele for each distinct path covered by the haplotypes passing through the bubble. In the Figure, two alleles are listed for the superbubble, one which seems to follow the reference path (all blue edges) and the other one passing through all variant edges. However, in reality the superbubble and vcfwave VCFs might contain many more alternative alleles. So I think it would be useful to show haplotype paths traversing this bubble in the figure, otherwise it is unclear why there are exactly two alternative alleles listed in the superbubble / vcfwave VCFs. Also two minor points regarding the same Figure: why is the reference allele (REF) not listed for the superbubble and vcfwave VCFs? And why is the second ALT sequence of the superbubble VCF one base shorter than the other one?

Response: Thank you for this suggestion. In the revised manuscript, we updated the corresponding figure panels and caption to make the relationship between haplotype paths and VCF records explicit. We now show the correct REF and ALT fields for the illustrated examples, corrected the off-by-one issue in the ALT sequence, and clarified in the caption that the number of ALT alleles in superbubble- and vcfwave-based VCFs depends on the number of distinct haplotype traversals through the bubble, with the displayed example intentionally simplified for exposition.

Minor Comments

1.8. Original comment: The github link:
https://github.com/ShenghanZhang1123/graph_var_analysis
is not accessible.

The version of all software tools used in the analyses described should be added.
Response: We have fixed the analysis-scripts link and ensured accessibility. We have also added software-version information where appropriate and improved the repository documentation.

REVIEWER #2

2.1. Original comment: The authors describe a novel way to define and catalog variants in pangenome graphs that avoids the problems in current bubble-based approaches. Data and code are both available. Most of my comments concern parts of the text where I would appreciate more detail/elaboration to make it easier to understand.
Response: We thank the reviewer for their positive review and excellent suggestions.

2.2. Original comment: In Supplementary Note, Section 8, a toy example for each variant edge category would be helpful to the readers.
Response: Agreed. We added a compact toy example for each category of variant edge to Section 8 of the Supplementary Note, so that readers can see each class in a minimal worked example rather than only through the formal definitions.

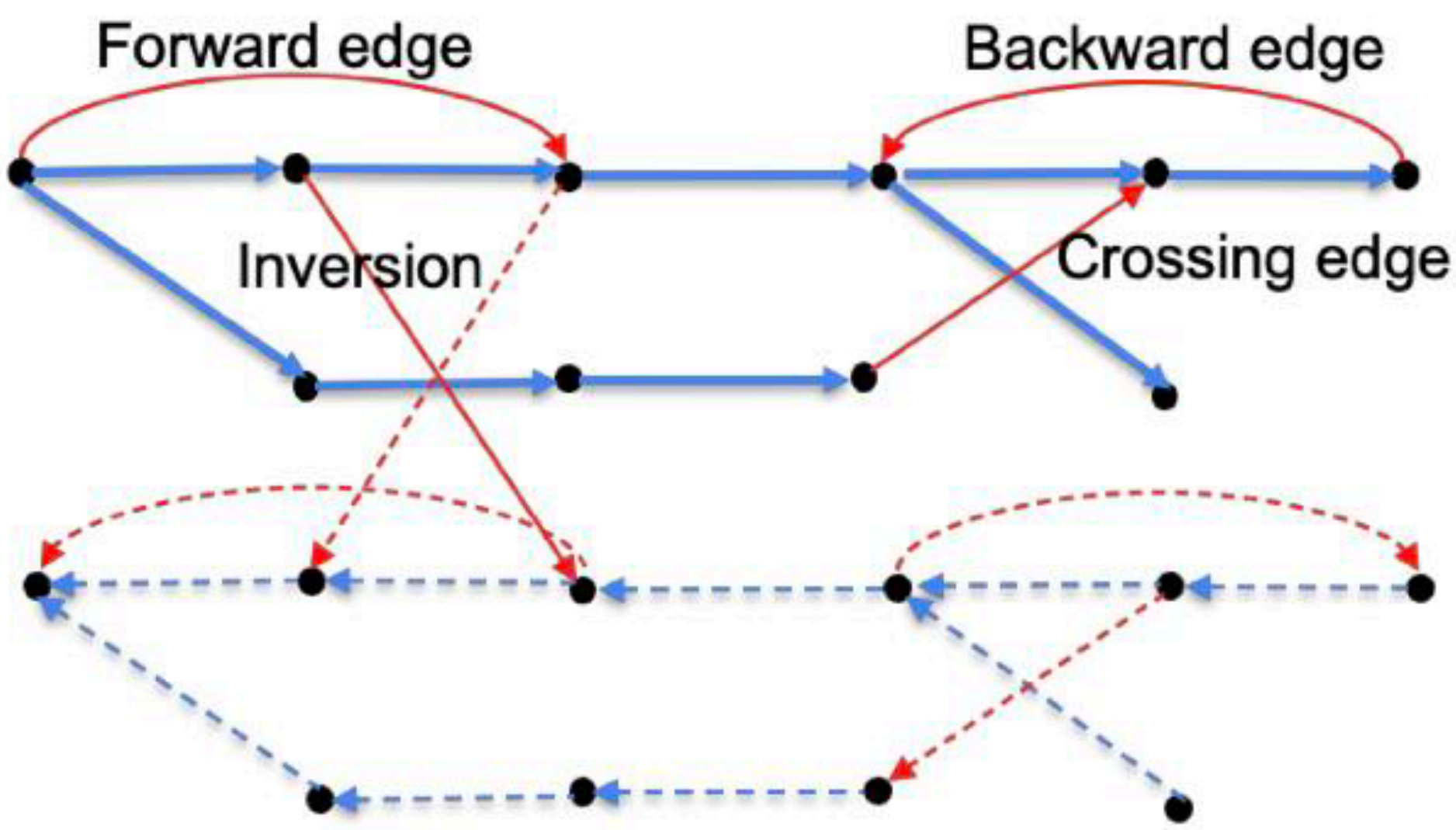

2.3. Original comment: "The larger number of deletions is mostly due to an ascertainment effect, that deletions are more likely to be multiallelic." Can the authors explain why this occurs more often with deletions than with insertions?
Response: We have clarified this point in the Results section "Multiallelic superbubbles" and expanded the explanation in the caption of Supplementary Figure 11. In the main text, we now explain that "The larger number of deletion superbubbles is mostly due to an ascertainment effect. If a superbubble contains several alleles, just one of which is empty, then the empty allele usually has a frequency less than 0.5, and the linear reference usually has the non-empty allele. If the linear reference has the non-empty allele, then the superbubble is classified as a deletion." We also made this interpretation explicit in Supplementary Figure 11, which quantifies the much higher proportion of deletion superbubbles that are multiallelic compared with insertion superbubbles.

2.4. Original comment: "This approach relies on our variant definition because there is no similar way to simplify the graph without first discarding unwanted edges." Can the authors elaborate? Which approach? Pruning + tip deletion + contraction? Or perhaps the authors meant "This approach is necessary due to our variant definition…"? How do you define unwanted edges?

Response: Thank you for pointing out this ambiguity. In the original text, "this approach" referred to the three-step graph simplification procedure we used in the Complex regions analysis (pruning, tip deletion, and contraction). In the revised manuscript, we have clarified this in two ways.

First, in the Methods section "Simplification of Complex Variant Structures", we now explicitly describe the pipeline as: (i) removing "small" variant edges whose combined reference and alternative allele length is <50 bp; (ii) pruning tip-like structures that do not contribute to connectivity; and (iii) contracting linear paths whose internal nodes have degree two and are traversed by all remaining haplotypes (obligatory edges). We also explicitly state that these edges cannot serve as reference or alternative alleles for any pantree variant, so this procedure does not change the set of variant edges.

Second, in the Results "Complex regions" subsection, we have rephrased the sentence that previously referred to "unwanted edges" to avoid this vague terminology and instead describe the same procedure in terms of small variant edges and obligatory linear segments.

2.5. Original comment:

Can the authors release a standalone script/pipeline to run the techniques applied to simplify the graphs and obtain the simplified graphs in Figure 4? Or add an optimal parameter in pantree to emit the simplified graph in GFA format?

Response: Yes. We added this functionality as a documented command-line option in pantree, allowing the simplification procedure used for Figure 4 to be run directly and the simplified graph to be emitted in GFA format. We also added a brief usage example to the documentation.

2.6. Original comment: How to compare variants made with different pangenome trees of the same pangenome graph? *Is there a way to 'normalize' the representation to represent the same variant in the same way?*

Response: For a fixed pangenome graph, the underlying haplotype walks and the pairwise symmetric differences among them are invariant, and the set of variant edges is one choice of basis for the resulting vector space (Supplementary Note, Section 2). There is no way to "normalize" variant representations such that they are non-reference tree dependent; doing this is not even possible with a linear reference, as many variants may be affected when the linear reference is changed. However, it is possible to switch between representations; this is a change of basis operation. Given the genotype of a sample, pantree is able to calculate the number of times that every edge in the pangenome graph is visited, and in particular, it can calculate the visit counts of variant edges with respect to a new reference tree. We also analyzed the number of variants that are discovered with different choices of reference tree (see response to comment 2.7).

2.7. Original comment: Is there an optimal way to choose the reference tree (for example, to minimize the amount of variation output), or are all possible reference trees equivalent? I see authors defined a heuristic to maximize the allele frequency of reference alleles greedily. How does the heuristic impact the final output? What other heuristics would be worth exploring?

Response: For a fixed pangenome graph, the haplotype walks, their pairwise symmetric differences, and the dimension of the variant-edge space are invariant, but different reference trees correspond to different choices of basis for that space and therefore lead to different labels and groupings of variant edges. There is no optimal reference tree as far as we can tell. In our revision, we added an alternative definition for the sake of comparison, with a different motivation compared with the main definition. This "haplotype-contiguous" tree chooses an individual haplotype follows it for as long as possible. When forced to switch haplotypes, it chooses a new haplotype according to a user-specified priority order (e.g., GRCh38, CHM13, HG002, …), falling back to the weighted heuristic when the priority list is exhausted. The motivation for the original choice is that reference alleles should usually be major alleles; the motivation for this choice is that it can be convenient when assigning coordinates when a

reference tree path follows just 1-2 unique haplotypes. In the revised manuscript, we describe these choices and compare the number of variants that are found using each reference tree; we find that the overall number of variants, and their distribution of variant types, is very similar between the two choices of reference tree (see the figure below).

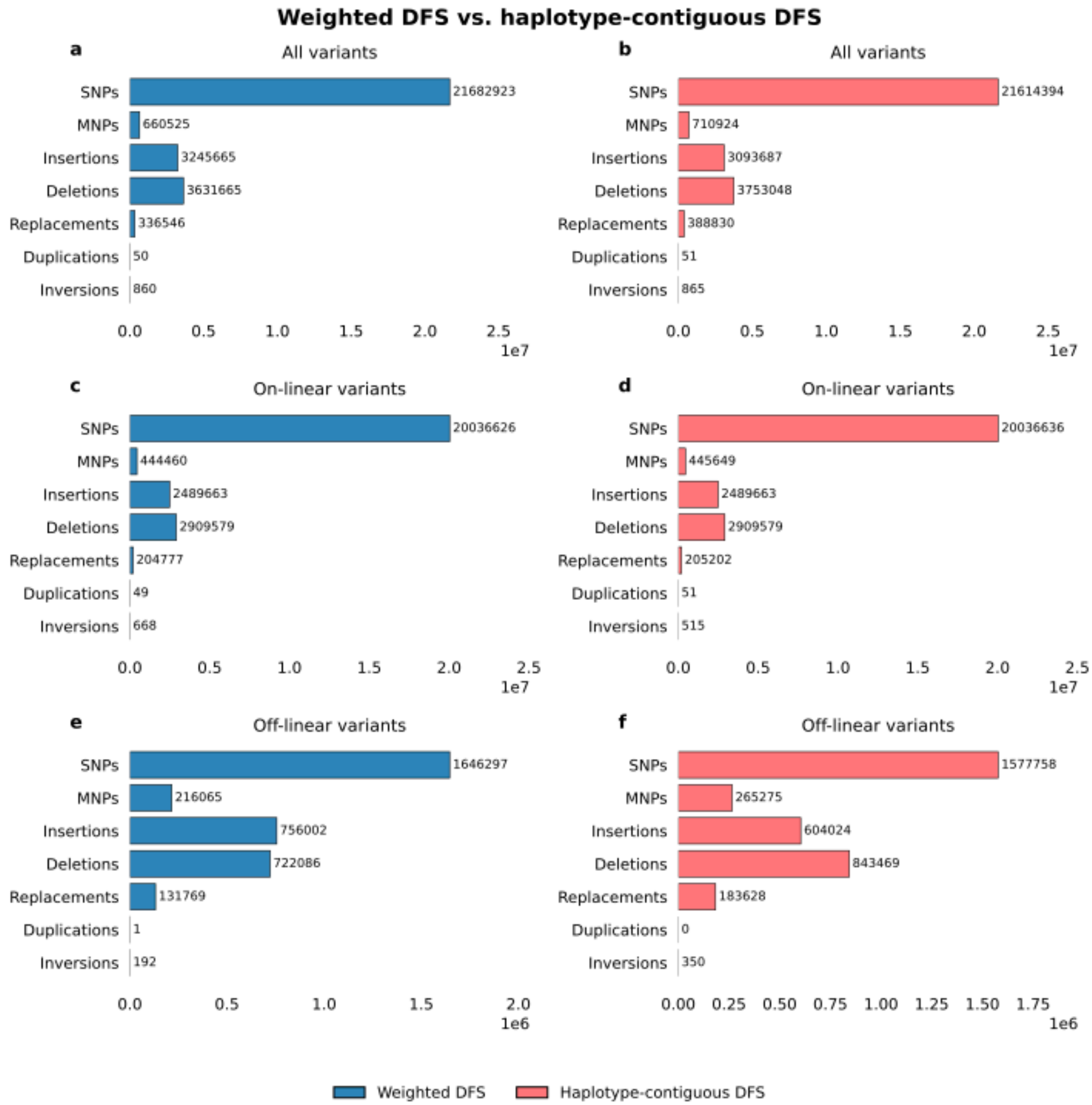

Supplementary Figure 12. Comparison of variants defined by weighted DFS and haplotype-contiguous reference trees. Variants were called on the same pangenome graph using two different reference-tree constructions: a weighted depth-first search (DFS), which prioritizes edges traversed by many haplotypes, and a haplotypecontiguous DFS, which prioritizes continuity along selected haplotypes. Panels a,b show counts for all variants, panels c,d show on-linear variants, and panels e,f show offlinear variants. The left column corresponds to the weighted DFS tree and the right column to the haplotype-contiguous DFS tree. Although individual variant edges differ

between the two reference trees, the overall numbers of variants and the distribution of variant types are highly similar.

2.8. Original comment: Authors prefer to define variant positions against an acyclic reference genome path, thus ruling out working with the type of graphs created with methods like PGGB. Is there a way to extend pantree to work with PGGB graphs, or edit PGGB graphs to allow users to use pantree on them?

Response: It is possible to do so, but we do not recommend this. When a reference genome revisits nodes, doing so introduces two related complications: nodes on reference-genome cycles no longer have a unique position, and repeated or merged nodes may represent sequence aligning to multiple distinct loci on GRCh38. In that setting, a pantree variant edge can capture differences among paralogous copies within one genome rather than homologous sequences across genomes, and there is no clear way to assign it a unique locus-specific coordinate or encode it cleanly in a single-locus VCF record. We added the following paragraph to the Discussion section:

Whereas the GRCh38 reference genome forms a simple path—never visiting any node twice—in the Minigraph-Cactus graph, it does not do so in the pangenome graph inferred using PGGB, and other reference genomes (e.g., CHM13) do not generally form a simple path in either graph. Our framework can in principle handle linear reference genomes with repeated nodes (Supplementary Note, Section 12), but doing so introduces two related complications. First, nodes that lie on reference-genome cycles no longer have a single position, so multiple reference positions become superimposed in the graph. Second, when repeated or merged nodes represent sequence aligning to multiple distinct loci on GRCh38—as can occur in PGGB graphs—a pantree variant edge may simultaneously capture differences among orthologous haplotypes and divergence between paralogous copies of the reference. The interpretation of the variant edge changes fundamentally, from an inter-genome difference to an intra-genome difference. For this reason, we restrict our analyses to Minigraph-Cactus graphs in which GRCh38 follows an acyclic path; our software can be applied to PGGB graphs, but we do not recommend doing so.

REVIEWER #3

3.1. Original comment: This paper proposes a novel method to identify and classify genomic variants in a pangenome graph. This a timely and well needed methodological contribution since pangenome graphs are increasingly built and used for various organisms. There are very few existing approaches for identifying variants in such graphs and they suffer for various drawbacks. In particular they are biased/limited by the use of a reference genome, some variants are missed or redundantly represented and they are not classified by types.

The authors introduce a novel data structure, the reference tree, that has the potential to remove these limitations and is an original and very promising way of addressing this problem. The method is implemented in an open source tool called pantree available on github. The authors apply their method on the human draft pangenome graph built with Minigraph-Cactus and characterized the variant set they obtained, and compared it to another existing approach (superbubbles).

The main contribution of this paper lies on the methodological side. The novel method proposed is a very important and valuable contribution that will certainly influence future methodological work on pangenome graphs as well as how we study variants in such graphs. However, the presentation of the method lacks clarity and the experiments do not allow a proper validation of the implementation nor a fair comparison with existing approaches.

Response: We thank the reviewer for their kind assessment and their very thoughtful review. In the revised manuscript, we strengthened the exposition in the main text, added a pipeline overview and early concrete examples, expanded the validation against external truth sets and raw-VCF comparisons, and improved the discussion of alternative bubble-based methods and

reference-tree dependence.

3.2. Original comment: Major concerns with the presentation of the method:
The description of the method in the Result section of the main manuscript is not enough formal and lacks some explanation to understand the concept and intuition behind the method. On the contrary, the description of the method in Supplementary Material is much more technical and formal, which is a good thing, but it may be difficult to read for non computer-science or graph theory readers. A description of intermediate precision is missing from the main text. For instance, how the reference tree is built is not explained in the main text, nor the important role of the reference genome in its construction. The full pipeline of the implemented method, showing the successive steps of computation, is not described anywhere and would probably help the reader. An algorithm of the heuristic implemented for chosing the edges of the spanning tree would also be helpful. Another suggestion would be to describe the method more formally in the main text but without the bi-directionality aspects...

Response: We thank the reviewer for this helpful suggestion. In the revised manuscript, we added a mid-level Methods subsection titled "Pantree pipeline and reference-tree construction" (Methods, immediately before "Analysis Details"). This subsection gives an overview of the full pantree pipeline, explains how we build the weighted depth-first reference tree (including the heuristic based on haplotype counts), and describes the specific role of the linear reference genome: GRCh38 is forced to form a branch of the tree and is used to define positions for all nodes and variant edges. We also summarize how variant positions, reference and alternative alleles, and variant types are derived from the reference tree (with pointers to the conceptual definitions in the Results section and the detailed VCF encoding in the "VCF Specification" subsection), and we briefly describe the alternative haplotype-contiguous reference tree and how pantree genotypes sample haplotypes with respect to either tree. We hope this intermediate exposition makes the method clearer and more accessible to readers who do not wish to follow the full formalism in the Supplement.

3.3. Original comment: Figure 1 could be improved by defining and showing the variants represented in the toy graph and their types. I appreciated the examples of representation of variants as superbubbles and in the pantree VCF in Figure 3 and Suppl Figs 8 and 10, such examples could be shown earlier in the main text.

Response: We revised Figure 1 so that it now introduces concrete variant examples and their representations much earlier in the manuscript. In particular, panels (a)–(c) now show simple variants, an interlocking superbubble, and a nested superbubble, and the caption explicitly explains how bubble-based approaches represent the latter two examples. The revised caption states, for example, that in panel (b) "bubble-based callers typically treat the whole structure as one multiallelic site, reporting a reference allele 'TTCATG' and two alternative alleles 'TTAATG' and 'TTCTTG', rather than two separate SNPs," and in panel (c) that "bubble-based approaches again represent this as a single site with reference allele 'T' and two alternative alleles 'TATGC' and 'TAGGC,' obscuring the distinction between the insertion and the SNP on the insertion." These changes move the key conceptual examples earlier in the manuscript and make the limitations of bubble-based representations more concrete.

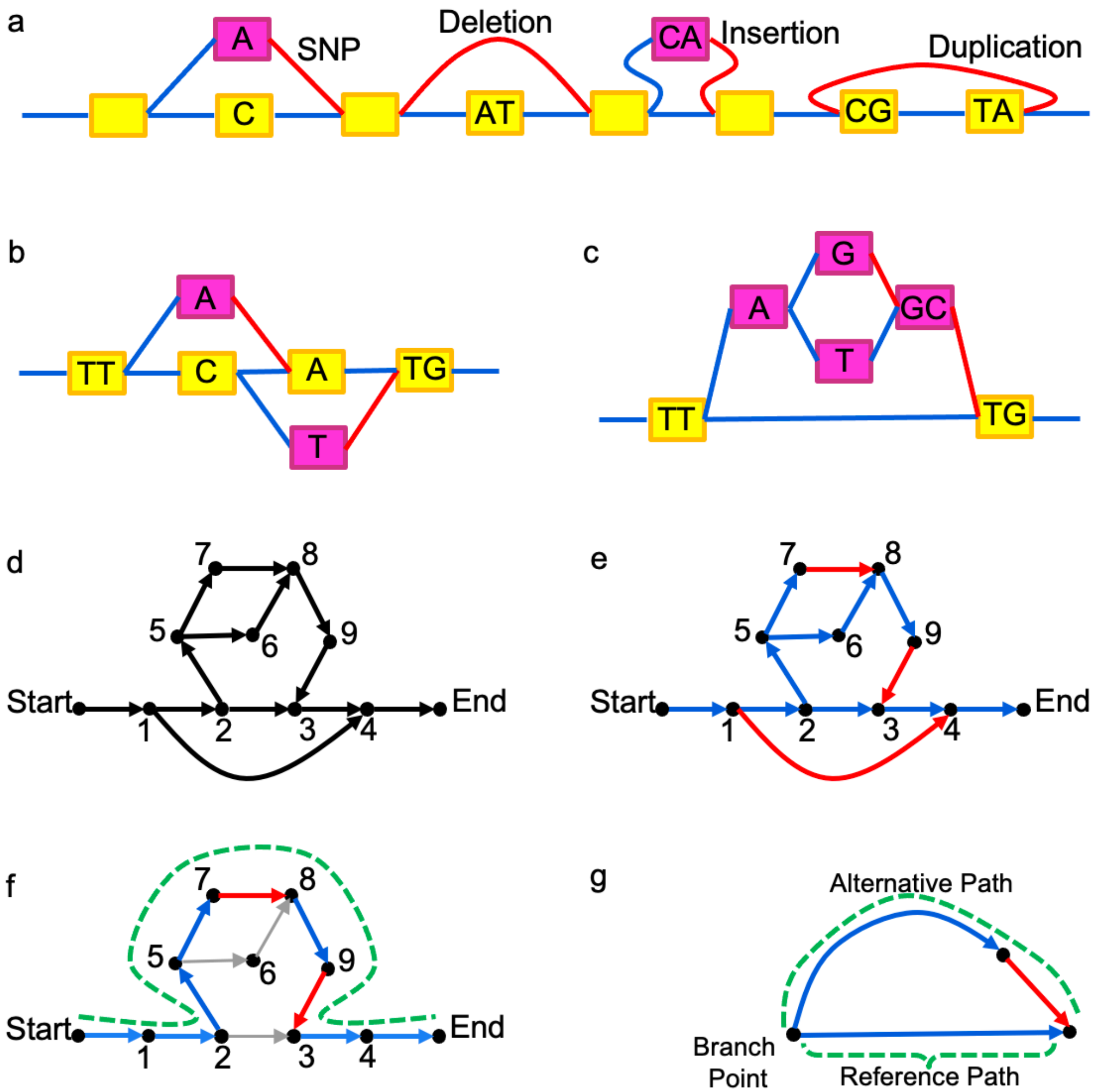

Figure 1: Definition of a pangenome variant. (a) Simple small and structural variants along a linear reference (yellow nodes): from left to right, a SNP changing C:A, a deletion removing “AT”, an insertion of “CA”, and a duplication of “CGTA”. Pink nodes mark sequence that differs from the linear reference. (b) A superbubble with two interlocking SNPs. The superbubble has reference allele “CA” and two alternative alleles “AA” and “CT”; pantree finds two separate SNPs. (c) A superbubble containing an insertion with a SNP on the inserted sequence. The superbubble has reference allele “T” and alternative alleles “TATGC” and “TAGGC”; pantree finds one insertion and one SNP. (d) A toy example graph with 11 nodes. (e) A reference tree (in blue) and corresponding variant edges (in red). (f) A walk representing one individual haplotype (in green), with its variant edges highlighted (red). (g) Paths corresponding to the reference and alternative alleles of a variant edge.

3.4. Original comment: Similarly, as a major feature of the approach is to catalogue variants in

different types, a figure showing the different types of variants and their associated properties of variant edges would improve the clarity.
Response: Agreed. We added a compact toy example showing each category of variant edges to section 8 of Supplementary note.

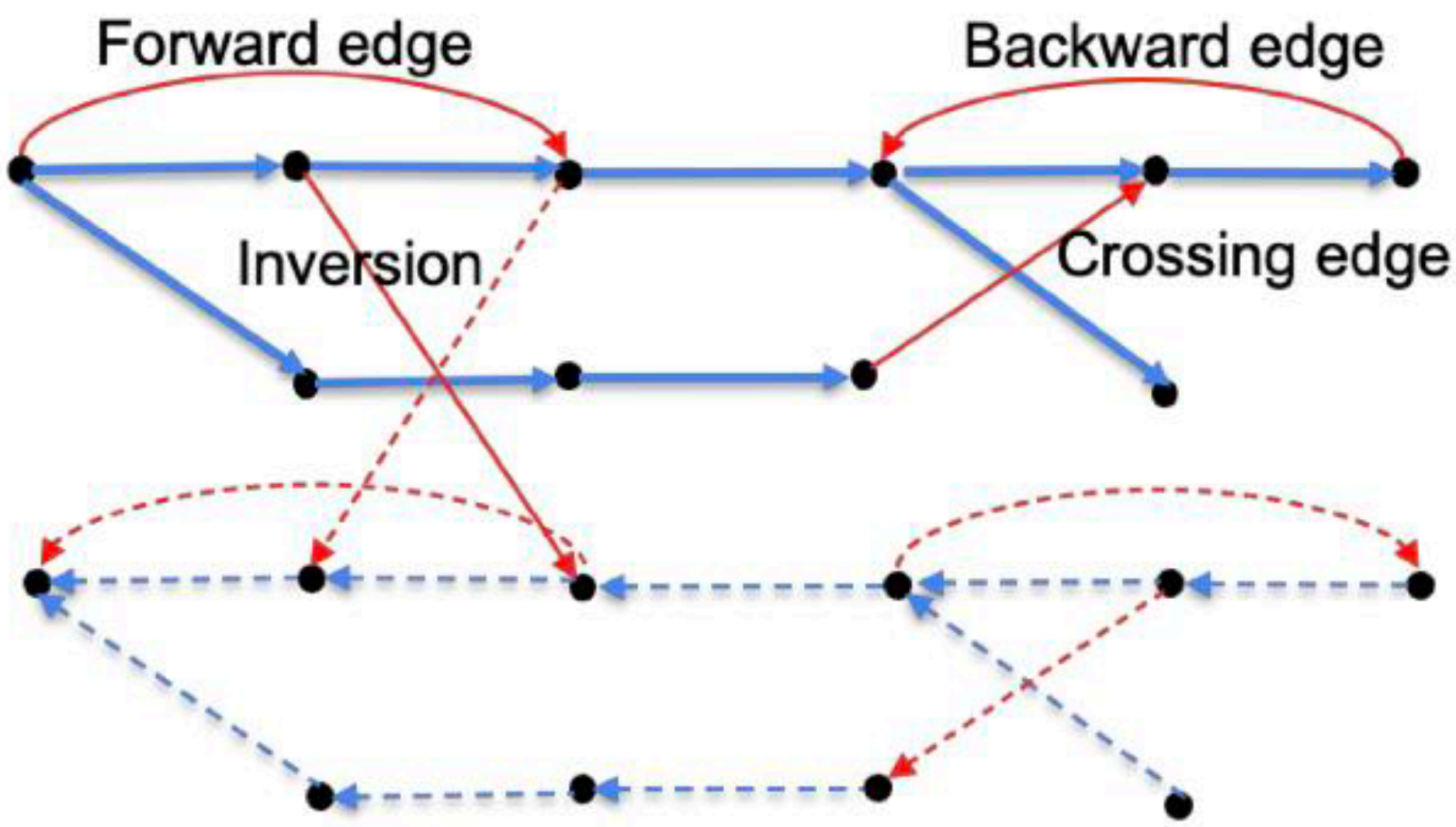

3.5. Original comment: 2. Major concerns with the empirical validation of the method and comparison to superbubbles:
The comparison of obtained variants with pantree is restricted to superbubbles of level 0. Fist, this should be clearly stated in the Result section, not just in the Method section, since it explains why pantree output much more SNPs. Secondly, I find it too restrictive. The authors should compare their variants to higher levels of superbubbles. The authors justify this choice by declaring that 1) "higher-level bubbles do not yield variant definitions with well defined positions along the linear reference genome" and 2) "these bubbles introduce redundancy: the same variant may appear in multiple bubbles across different levels, each offering a different representation". I do not agree with these statements: if the authors restrict their comparison to SNPs (as it is the case in presented results) and to variants involving the reference genome, variant positions are well defined (as output by vg-deconstruct in a VCF format positioned on the reference genome) and without any redundancy. With such a comparison, we could better interpret the differences in number of SNPs: this will validate that pantree not only does not miss any simple SNPs but also no nested ones. This will better highlight which SNPs are missed by the superbubble approach (non-reference SNPs and those in interlocking and overlapping bubbles), compared to the vcfwave comparison which gives false negatives (SNPs not found by pantree) due to re-alignment artefacts (as stated by the authors).
Response: We were previously mistaken: our manuscript did include higher-level superbubbles, specifically those that were able to be included in the raw VCF file. We apologize for our confusion and have fixed our error in the revised manuscript.
Specifically, in our SNP benchmarking analysis (Results, "Catalogue of variants"; Fig. 3a–b and Supplementary Fig. 5), we compare pantree SNPs with all SNPs in the vg-deconstruct raw VCF. The raw VCF is derived from the full superbubble hierarchy and includes SNPs from nested bubbles (levels > 0) if they have GRCh38 coordinates; in particular, this includes SNPs nested inside of deletions. We now state explicitly that this SNP-level comparison already assesses whether pantree recovers SNPs detected by vg-deconstruct across all superbubble levels, including nested SNPs, and show that pantree recovers essentially all such SNPs while

additionally reporting many non-vg-deconstruct SNPs.
On the other hand, in the multiallelic-superbubble analysis (Results, "Multiallelic superbubbles"; Fig. 4), we deliberately restrict to level-0 superbubbles, which produce a partition of variant edges and therefore provide a natural way to study which local graph structures contain various classes of variants. If higher-level bubbles were included in this analysis, it would lead to double-counting of variant edges. We have revised the Results text, Figure 4 caption, and the Methods section to state clearly that this analysis is level-0 only and to explain the motivation for this choice.
3.6. Original comment: Similarly, the comparison is restricted to SNPs, and disregards other variant types such as small indels or larger SVs, which are harder to find and characterize in pangenome graphs and where the benefits of pantree are probably greater. Here again, by looking at all levels of superbubbles but restricting to variants involving only a pair of genomes (including the reference genome), variant positions and alleles should be well defined and comparable between the two approaches. This would consolidate the validation of the implementation of the method for all variant types, and probably show if the benefits are of the same order of magnitude for all variant types.
Response: We thank the reviewer for this suggestion. As the raw VCF already contains GRCh38 variants arising from higher levels of the superbubble hierarchy (see our response to comment 3.5), we used it to benchmark non-SNP variants between the HPRC raw VCF and the pantree VCF on chromosome 20. In the revised manuscript, we added the following paragraph to the Results: "Comparing non-SNP variants between methods is more challenging because for non-SNPs, the same sequence change can sometimes be represented in multiple ways. … We selected superbubbles in the chromosome 20 raw VCF for which GRCh38 and CHM13 have different alleles, and we classified these as biallelic or multiallelic and as an insertion, deletion, SNP, or other complex event. For biallelic variants, we required an exact match of chromosome, position, REF, and ALT between the raw and pantree VCFs. Under this strict criterion, pantree recovered all biallelic insertions (1,600/1,600), all biallelic deletions (1,826/1,826), and 84.9% of biallelic 'other' variants (399/470)." We further explain there that multiallelic sites do not admit a natural one-to-one comparison because pantree often decomposes a superbubble into several variant edges, whereas a single superbubble may combine alleles that pantree represents as distinct loci. We also added the corresponding Methods subsection, "non-SNP benchmarking against the raw VCF," which explains how the raw variants were selected and classified. Together, these additions validate pantree's implementation beyond SNPs while clarifying that multiallelic discrepancies are largely representational rather than missing variants.
3.7. Original comment: The superbubble approach implemented in vg-deconstruct can output non-reference variants by changing the path of the reference given as a parameter. I agree this is not convenient, nor efficient to re-run vg-deconstruct for all possible reference paths. However, could the authors use this approach to validate or compare at least a subset of the non-reference variants obtained with pantree?
Response: This is a good suggestion. To validate a subset of non-reference variants using a superbubble-based method, we re-ran vg-deconstruct on the Year 1 chr20 graph (constructed with GRCh38 as the guiding reference) using a CHM13 chr20 contig as the reference path and obtained a raw SNP VCF on that contig. We then restricted to pantree SNPs whose reference alleles traverse the same CHM13 contig and compared them to SNPs from the vg-deconstruct raw VCF on that path In this region, pantree recovered 100% (24,570/24,570) of the vg deconstruct SNPs while calling an additional 2,996 SNPs. This analysis is now described in the Results section "Validation and methods comparison" and in the Methods subsection "SNP benchmarking on a CHM13 contig."
3.8. Original comment: The authors claim that their approach can be applied to other graphs

than those built with Minigraph-Cactus, such as those obtained with PGGB. They should show some empirical results on such graphs.

Response: This concern overlaps with Reviewer 2, comment 2.8, and we addressed it in the same revised Discussion paragraph. We now explain that when a reference genome does not follow an acyclic path, or when graph nodes represent sequence aligning to multiple distinct loci on GRCh38, a pantree variant edge can mix differences among homologous haplotypes with differences among paralogous copies within one genome. In that setting, neither the coordinate nor the biological interpretation of the variant edge is clear, and there is no clean single-locus VCF representation. For this reason, the revised manuscript explicitly states that we restrict our analyses to Minigraph-Cactus graphs in which GRCh38 follows an acyclic path and recommend caution when applying pantree to PGGB graphs.

3.9. Original comment: As the main contribution of this paper is an implemented method, which is a meaningful alternative to the superbubble approach (such as implemented in vgdeconstruct), it would be interested to show some performance results: how does it compare to vg-deconstruct in terms of running times and memory consumption?

Response: We agree that a concise summary of runtime and memory usage is useful. We added a short "Performance and Resource Usage" subsection to the Methods. The pipeline was parallelized by chromosome; on chromosome 1, it required 33.4 GB of memory and 6.7 hours of wall time, and 55 hours of CPU time (Supplementary Table 15). Because pantree is run once per graph to generate the reference tree and VCF, we view this as a one-time preprocessing cost rather than a per-sample burden.

3.10. Original comment: 3. Major concerns with the introduction:
Some references to other methods to find variants in pangenome graphs are missing in the introduction: vg-deconstruct, gfatools bubble, Bubble Gun.

Response: We thank the reviewer for pointing this out. In the revised Introduction, we now explicitly mention vg-deconstruct, vcfwave, gfatools bubble, and Bubble Gun as examples of bubble-based approaches for identifying variants in pangenome graphs. We briefly describe how these methods work (detecting superbubbles or related bubble structures and emitting variants along a linear reference path) and contrast them with pantree, which defines variants as edges with respect to a reference tree and can catalog alleles nested inside non-reference sequence. This clarifies how pantree fits into the landscape of existing graph-based variant discovery methods.

3.11. Original comment: I do not agree with the statement "a variant is a superbubble", the authors should talk about the hierarchy of bubbles, not all bubbles in the hierarchy require that source and sink nodes are traversed by all haplotypes, and this allows to represent more finely small nested variants.

Response: We agree and revised the Introduction accordingly. We removed the statement that "a variant is a superbubble" and now describe existing approaches more carefully in terms of bubbles, superbubbles, and snarls, including the fact that nested variants can arise within the hierarchy of these structures. The following paragraphs in Introduction explains this:
"Currently, variants in pangenome graphs are defined as different kinds of "bubbles": loosely, regions where two walks split apart and then merge back together (Onodera et al., 2013; Paten et al., 2018). In a directed graph, a superbubble is such a region with a single entry node and a single exit node, such that every directed path from the entry to the exit is contained within the bubble. The bidirected generalization of a superbubble, allowing for inversions, is called a snarl (Paten et al., 2018). Snarls can be organized into a nested hierarchy. However, a challenge is that snarls can contain many long alleles, some of them differing by only a few nucleotides despite being kilobases in length. In such regions, hierarchical decomposition succeeds for properly nested events, such as a long insertion containing several SNPs, but it cannot decompose interlocking structures, and nested sites do not in general inherit unique coordinates on the chosen linear reference path (Liao et al., 2023; Paten et al., 2018).

Liao et al. used three methods to work around these limitations. First, vg deconstruct was used to identify snarls and emit a raw VCF for level-0 snarls as well as nested snarls traversed by the reference allele (for example, SNPs nested inside of deletions). Second, they introduced the vcfwave pipeline, which takes the allele sequences from the raw VCF records emitted by vg deconstruct, realigns them to the linear reference, and decomposes the resulting alignments into "primitive" variants. This approach recovers small variants nested within deletions and other replacement-like alleles, but not within insertions, because the reference allele is still required to lie on the linear reference genome. Third, Liao et al. also implemented a traversal-based decomposition procedure (distributed as the resolve-nested-genotypes tool) that uses the alleletraversal annotations in the raw VCF: for each raw VCF site, it compares the graph traversal of the reference allele to the traversals of the alternative alleles and extracts the implied nested SNPs and indels as separate biallelic variant records. Unlike vcfwave, which decomposes variants by realigning allele sequences back to the linear reference, this third approach decomposes them by analyzing how haplotype paths traverse the graph site itself."

3.12. Original comment: The limitations of bubbles and their hierarchy should be explained in a more rigorous way. Giving precise examples of interlocking and redundant variants with bubbles, together with their representation with pantree could highlight further the benefits of the proposed method. I appreciated the examples in fig. 3 and for instance supp figures 10, but they seem to be rather particular examples and arrive late in the reading.

Response: Thank you for this suggestion. In the revised manuscript, we moved a clearer concrete example earlier in the presentation and tightened the schematic explanation of interlocking and redundant variants. In particular, the revised Figure 1 and its caption now introduce the contrast between bubble-based representations and pantree earlier in the manuscript, while the later multiallelic-superbubble analysis provides a more systematic classification of overlapping, nested, and interlocking cases.

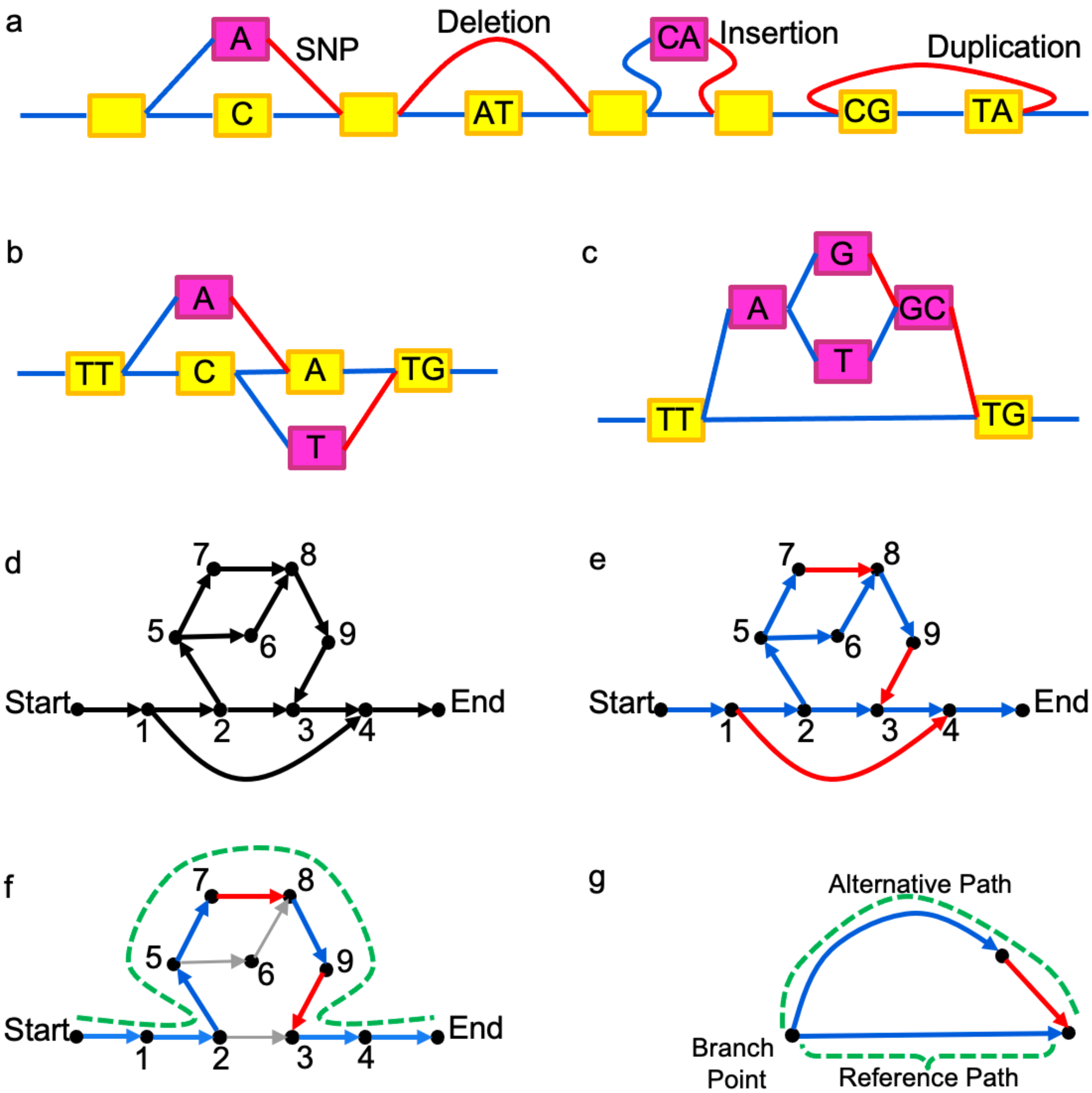

Figure 1: Definition of a pangenome variant. (a) Simple small and structural variants along a linear reference (yellow nodes): from left to right, a SNP changing C:A, a deletion removing "AT", an insertion of "CA", and a duplication of "CGTA". Pink nodes mark sequence that differs from the linear reference. (b) A superbubble with two interlocking SNPs. The superbubble has reference allele "CA" and two alternative alleles "AA" and "CT"; pantree finds two separate SNPs. (c) A superbubble containing an insertion with a SNP on the inserted sequence. The superbubble has reference allele "T" and alternative alleles "TATGC" and "TAGGC"; pantree finds one insertion and one SNP. (d) A toy example graph with 11 nodes. (e) A reference tree (in blue) and corresponding variant edges (in red). (f) A walk representing one individual haplotype (in green), with its variant edges highlighted (red). (g) Paths corresponding to the reference and alternative alleles of a variant edge.

3.13. Original comment: "This definition satisfied our intuition": which one?

"it has the right mathematical properties": no mathematical properties have been stated before
Response: We agree that this wording was vague. In the revised manuscript, we removed the phrases “this definition satisfies our intuition” and “it has the right mathematical properties” and replaced them with a more explicit description of what the definition guarantees.

3.14. Original comment: 4. Major concerns with the software:
I successfully installed the software, but it was not straightforward as the Readme lacks instructions for installion.
I successfully run the software on the provide toy examples but it was more complicated on my own pangenome graphs as information about the desired gfa format of input file was missing in the Readme. For instance, the gfa should contain W lines and not P lines, L lines should be placed before W lines in the file (which was not the case for my graph obtained with Minigraph-Cactus)...
the authors present in the paper a tool to simplify the graph, however there is no executable for it in the repository (nor documentation in the Readme). It could be useful to provide such a tool based on the reference tree with a gfa output.
I could not access the scripts to reproduce the analyses, stated to be available à this github repository, which is not a valid link: https://github.com/ShenghanZhang1123/graph_var_analysis
Response: Thank you for this feedback. We have:

- Added installation instructions to the README
- Added support for W lines to come before L lines
- Added support for P lines
- Made the scripts repository public (we apologize for this).
- Added the simplify_subgraph function to the CLI with a .gfa output, as suggested.

3.15. Original comment: 5. Minor comments:
Results on complex regions: it seems to be the same regions analyzed in details in the draft human pangenome paper (Liao et al 2023), could the authors compare the findings and biological interpretations between both analyses?
supp fig. 8: why was vcfwave applied on this superbubble, as it is not multi-allelic?
p.6 and 7 of suppl. material, L is not defined, there seems to be 2 proofs for Theorem 4.1 ;
p.10 of suppl. material sup Fig 3, what are x and y?
p.11 of suppl. material, sup. fig. 4, it would help understand the figure if the paths on the pangenome graphs were shown. The legend contains typos: "traversin" "and This".
Response: Thank you for these helpful comments. We addressed these points in the revised manuscript and supplementary materials.
For the comment on the complex regions, we did not add a full biological comparison with Liao et al. (2023), since the two analyses use different variant representations and such interpretation is beyond the scope of this manuscript. However, in the Methods, “Complex Regions” section, we now state that we compared the graph structures in these loci to those shown in Liao et al. (2023) in order to refine the node IDs and positions used in our analysis.
For Supplementary Figure 8, the caption already explains why vcfwave appears in that example: the figure shows a vcfwave-specific SNP that is inconsistent with the graph.
We also corrected the remaining supplementary-material issues noted by the reviewer, including the missing notation/variable definitions, the duplicated-proof issue, and the typographical and figure-clarity problems.

Referees’ reports, second round of review

Reviewer #1: 1.1. OK

1.2. OK

1.3. The authors extended their comparison of pantree and the raw HPRC VCF to include indels, however, report statistics only for biallelic non-SNP variants based on exact matches. The authors should add comparisons of all SNPs, indels and SVs between pantree and vcfwave based on state-of-the-art tools like RTG vcfeval (SNPs + indels) and truvari (SVs) that account for representation differences.

The authors also added an evaluation of pantree variant calls based on the GIAB HG002 small variant benchmark. However, the evaluations include only small variants. As I suggested in my original comment, the authors should additionally evaluate SVs.

1.4. What I was referring to in my original comment was the fact that assemblies as well as graph building methods are not perfect. As a result, some structures in the graph, especially in complex regions of the genome, might arise from such errors and not actually represent genetic variation. Hence my question is whether you have done any further evaluation on non-reference variants called by pantree to confirm that they represent actual genetic variation, which I think would be important to add.

1.5. I was referring to the set of novel, non-reference variants reported in these regions. The paper mainly emphasizes the method itself, which I really think is great and well designed. However, it would benefit from a stronger focus on biological implications. I agree that a full-depth association study is out of scope, but some application should be added that demonstrates pantree's ability to call non-reference variation (something the other tools cannot do) is valuable in practice.

1.6. What I referred to in my original comment was the fact that the set of non-reference variants is defined based on the specific pangenome graph they were called from. This makes it hard to compare sets of non-reference variants called from different pangenome graphs (constructed from different samples rather than the same set of samples), as well as analyze assemblies of new samples not represented in the graph. Given that there is no "fixed" pangenome graph and new pangenome graphs keep getting released containing better assemblies and additional samples, this issue is relevant in practice and therefore I think these aspects should be discussed in the text.

1.7. OK

1.8. OK

Reviewer #3: The authors have provided satisfactory responses to most of my previous comments. In particular, I appreciated the addition of paragraphs in the Methods section offering a mid-level description of the approach, the new examples in Figure 1, the additional experiments involving non-SNP variants and the comparison with vg deconstruct using an alternative reference genome, as well as the expanded details and references in the Introduction.

I consider that the manuscript is now acceptable for Cell Genomics, pending minor revisions.

Minor revisions:

- Time and memory performances: I appreciate that the authors have added information on runtime and memory usage. However, in its current form, this paragraph is not informative for the reader. It currently lacks both a comparison with the concurrent tool (vg deconstruct) and essential details about the computational environment (number of cores, number of threads, CPU frequency, CPU model, RAM, etc.), which should be added.

- Definition of superbubble (Introduction, p. 2): the authors define a superbubble as "a region with a single entry node and a single exit node, such that every directed path from the entry to the exit is contained within the bubble." I believe this definition is incomplete and should be revised. In particular, it omits the

property that the subgraph can be extracted without altering the topology of the remaining graph—i.e., there are no edges connecting internal nodes of the superbubble (excluding entry and exit nodes) to nodes outside the subgraph. As currently stated, the definition does not exclude the possibility of paths that traverse internal nodes without passing through the entry and exit nodes. This point is important, as it explains why vg deconstruct and vg snarls do not output interlocking bubbles, and this is one of the motivations of this work to answer this limitation.

- Figure 1: The description of subgraph 1c is incorrect, as it states that there is only one superbubble. In fact, there are two superbubbles in this graph, not just one. While it is true that vg deconstruct would report only the larger one, the smaller one is still a valid superbubble (and is detected by vg snarls). The caption of Figure 1 ("A superbubble containing an insertion with a SNP on the inserted sequence") and the text on p.4 ("but this is not the case in Figures 1b-c, each of which illustrates one superbubble containing two variant edges") refer only to superbubbles and not to vg deconstruct. These statements should clarify this distinction.

- Introduction p.3, the paragraph starting with "Beyond the snarl/superbubble framework" contains some repetitions: the method Billi is presented twice, as well as pangene. While I really appreciate the details about these other bubble-finder methods, a careful rewrite would improve readability.

- New Figure 3 still contains subfigures that are now in Figure 2.

- Supplementary Figure 12: It would be easier to compare the statistics between the two tree choices if the bars were displayed side by side within the same plots.

- p. 8 "A second is to post-process those raw VCF records with vcfwave": there was no "first" before.

Authors' response to the second round of review

Reviewer #1

1.3. Original comment: The authors should also compare their non-SNP variant calls to calls reported by vcfwave. So far, it seems that the comparison is based on SNPs only. While comparing indels or SVs is more difficult, there are tools available like hap.py, vcfeval or truvari that might be helpful here. In addition to this, the authors should compare their variant calls to external ground truth callsets to demonstrate that their approach produces correct results for reference-based variants. Options might be GIAB small and SV callsets or assembly-based callsets generated by tools like PAV (i.e. such produced by the HGSVC).

Comment 1.3. The authors extended their comparison of pantree and the raw HPRC VCF to include indels, however, report statistics only for biallelic non-SNP variants based on exact matches. The authors should add comparisons of all SNPs, indels and SVs between pantree and vcfwave based on state-of-the-art tools like RTG vcfeval (SNPs + indels) and truvari (SVs) that account for representation differences.

The authors also added an evaluation of pantree variant calls based on the GIAB HG002 small variant benchmark. However, the evaluations include only small variants. As I suggested in my original comment, the authors should additionally evaluate SVs.

Response: Both pantree VCF and vcfwave VCF are derived from the same assemblies and same alignments. In theory, they encode the same variation; they only differ in how this variation decomposes into variants. Comparing them with RTG vcfeval or Truvari really evaluate how well these tools can normalize variants, but not whether pantree is correct. More specifically, because pantree encodes off-GRCh38 variants that current tools ignore, these tools cannot properly compare pantree and vcfwave variants. Even for on-GRCh38 variants, vcfeval and Truvari are unable to normalize both small and large

variants, but without jointly normalizing all variants, it is not possible to perform strict haplotype-based comparison. These tools are fundamentally flawed.

We could compare HG002 small variants because GIAB provides confident regions for small variant evaluation where SVs detectable from assembly alignment are excluded. We do not have equivalent confident regions for all samples. A blind comparison between pantree and vcfwave with vcfeval would only underscore its limitations.

While vcfeval works well in small variant confident regions, Truvari does not even in SV confident regions. Having extensively studied long-read SV calling [PMID: 41384802 & DOI: 10.64898/2026.03.20.713111], we found that Truvari is unable to normalize SV representations in long VNTRs or segmental duplications for two reasons. First, all mainstream SV callers ignore SNPs and indels <30bp and thus cannot reconstruct the exact haplotypes. It is not possible for Truvari to perform strict haplotype matching. Second, in long VNTRs, the majority of SVs called by mainstream tools are wrong if we require strict haplotype matching. To avoid reporting an accuracy below 50% for most callers, Truvari is tuned to be highly tolerant with approximate SV calls. SV comparison often boils down to which SV representation Truvari likes better. Such a comparison can be misleading and may not reflect the true accuracy of SV callers.

Comment 1.4. What I was referring to in my original comment was the fact that assemblies as well as graph building methods are not perfect. As a result, some structures in the graph, especially in complex regions of the genome, might arise from such errors and not actually represent genetic variation. Hence my question is whether you have done any further evaluation on non-reference variants called by pantree to confirm that they represent actual genetic variation.

Response: Recently, HPRC has constructed a Year 2 graph featuring many new samples, new and improved assemblies for existing samples besides GRCh38 and CHM13, and a new alignment. Although neither this nor any pangenome graph can be viewed as a "ground truth" for variant detection, it should have fewer assembly errors due to improved methods, and its alignments should be of higher quality due to the larger number of samples. To evaluate whether non-reference variants called by pantree reflect stable sequence differences rather than artifacts of a particular graph construction, we performed a cross-graph reproducibility analysis. We added the following paragraph to the Results section:

"To assess whether pantree variants are reproducible across independently constructed pangenome graphs, we compared pantree variants between the Year 1 (46 samples) and Year 2 (233 samples) HPRC Minigraph-Cactus graphs for the longest CHM13 contig of chromosome 21. Both graphs contain the CHM13 T2T assembly, and we ran pantree with the haplotype-contiguous DFS approach using GRCh38 and CHM13 as priority samples, producing haplotype positions (HP) along both walks.] Since CHM13 uses the same assembly in both graphs, the position of a reference or alternative allele along that assembly can be used to compare the variants that are found in each graph. Some differences in the alleles themselves are expected, particularly because a graph with more samples may decompose a variant more finely, for example by representing a Year 1 deletion as a deletion plus a nested SNP in Year 2. Therefore, we used subsequence matching for Year 1 versus Year 2 alleles: a Year 1 variant was considered recovered if a Year 2 variant at the same CHM13 position had an allele that contained, or was contained within, the Year 1 allele. 99% of on-GRCh38 Year 1 variants were recovered

in Year 2 by subsequence matching, including 100% of SNPs, 97% of deletions, and 93% of insertions. We then considered non-GRCh38 variants with CHM13 haplotype positions. Of 7,754 such variants, 90% were recovered in the Year 2 graph, including 89% of SNPs, 92% of deletions, and 90% of insertions (Supplementary Table 16). Thus, non-GRCh38 variants are highly reproducible across graph versions, despite the Year 2 graph having ~5x more samples, different assemblies, and a different underlying sequence alignment. These results support the conclusion that most non-GRCh38 variants represent stable sequence differences rather than artifacts of a particular graph build, and they demonstrate a practical procedure for comparing variants across independently constructed graphs using haplotype positions as cross-graph coordinates."

We have added this analysis to the Results section and described the procedure in a new Methods subsection. We also revised the Discussion accordingly. In our view, this provides direct empirical support that most non-GRCh38 variants reported by pantree are stable across independently constructed graphs and are therefore unlikely to be artifacts of a particular graph build.

Comment 1.5. I was referring to the set of novel, non-reference variants reported in these regions. The paper mainly emphasizes the method itself, which I really think is great and well designed. However, it would benefit from a stronger focus on biological implications. I agree that a full-depth association study is out of scope, but some application should be added that demonstrates pantree's ability to call non-reference variation (something the other tools cannot do) is valuable in practice.

Response: While we maintain that in-depth functional characterization remains beyond the scope of this work, we have now added an analysis of coding versus noncoding regions as defined on the CHM13 assembly. This analysis is intended to address two questions: (1) what approach is needed for functional annotation of non-GRCh38 variants? (2) How often do these variants fall within genes or coding regions, understanding that this is just one dimension of genomic function?

We added the following paragraph to the Results section:

"We asked how often these non-GRCh38 variants fall in exons and coding sequences of annotated genes, focusing on the subset of non-GRCh38 variants with positions along the CHM13 haplotype positions in an updated pangenome graph, the Year 2 HPRC Minigraph-Cactus graph (see Methods). These CHM13 haplotype positions were intersected with gene annotations on the T2T-CHM13 assembly. Across all autosomes and chromosome X, 3,453,855 non-GRCh38 variants had available CHM13 positions; of these, 99,167 (2.9%) overlapped exons and 14,562 (0.4%) overlapped coding sequences, spanning 1,182 genes (Supplementary Table 17). These genes include many of major biomedical interest, including HLA genes, immunoglobulin heavy- and light-chain variable genes (IGHV, IGKV, and IGLV families), killer-cell immunoglobulin-like receptors (e.g., KIR2DL1 and KIR3DL1), and genes implicated in repeat-expansion and neurological disease, including HTT, ATXN1, ATXN2, and ATXN3 (Supplementary Table 19). For comparison, among on-GRCh38 variants with available CHM13 haplotype positions in the same VCF, 1,899,837 (4.3%) overlapped exons and 358,973 (0.8%) overlapped coding sequences (Supplementary Table 17). Thus, pantree identifies thousands of coding non-GRCh38 variants, including in medically relevant genes."

We also added the following sentence to the Discussion:

"At the genome-wide level, we identified 14,562 non-GRCh38 variants that overlap coding

sequence in 1,182 genes, including HLA, immunoglobulin, KIR, and repeat-expansion disease genes."
We believe this provides a concrete practical use case for pantree: it identifies coding non-GRCh38 variants in medically relevant genes that are not recoverable by methods such as vcfwave, which require the reference allele to lie on the linear reference genome. Our intent is not to claim functional interpretation of these variants, but to show that pantree reveals potentially important coding variation in non-reference sequence that would otherwise remain invisible."
Comment 1.6. What I referred to in my original comment was the fact that the set of nonreference
variants is defined based on the specific pangenome graph they were called
from. This makes it hard to compare sets of non-reference variants called from different pangenome graphs (constructed from different samples rather than the same set of samples), as well as analyze assemblies of new samples not represented in the graph.
Response: We thank the reviewer for highlighting this important practical issue. The new cross-graph reproducibility analysis that we added in response to Comment 1.4 also addresses this concern. In that analysis, we compare pantree variants between the Year 1 and Year 2 HPRC Minigraph-Cactus graphs on chromosome 21 using a shared coordinate system defined by the pair (GRCh38 position, CHM13 haplotype position). Because both graphs contain the same CHM13 T2T assembly, the CHM13 haplotype position provides a stable coordinate along a shared walk, which allows non-reference variants to be matched across the two graphs.
Thus, the same analysis serves not only as a validation that most non-GRCh38 variants are stable across independently constructed graphs, but also as a practical demonstration that non-reference variants can in fact be compared across graph versions when a shared haplotype is available as a coordinate system. We added the following paragraph to the Discussion:
"A related but different question is how to compare two pangenome graphs, as opposed to two reference trees for the same graph. In our chromosome 21 analysis, we show that this can be done in practice when the same haplotype is present in both graphs: the pair consisting of the GRCh38 position and the haplotype position along a shared CHM13 walk provides a practical cross-graph coordinate. Using this approach, most variants, including most non-GRCh38 variants with available CHM13 haplotype positions on the largest CHM13 contig, were recovered between the Year 1 and Year 2 HPRC graphs. This does not eliminate representation dependence, but it shows that cross-graph comparison is feasible in practice.
Thus, rather than only proposing a conceptual solution, we now provide an explicit procedure and an empirical example showing that cross-graph comparison of nonreference variants is feasible in practice when a shared haplotype can be used as a coordinate system."

Reviewer #3

Comment: Time and memory performances: I appreciate that the authors have added information on runtime and memory usage. However, in its current form, this paragraph is not informative for the reader. It currently lacks both a comparison with the concurrent tool (vg deconstruct) and essential details about the computational environment (number of cores, number of threads, CPU frequency, CPU model, RAM, etc.), which should be

added.
Response: We thank the reviewer for this suggestion. We revised the Performance and Resource Usage paragraph to include a direct comparison with vg deconstruct on chromosome 1 together with the computational environment for both runs. The revised text reads:
“Performance and Resource Usage. Our pipeline was parallelized by chromosome. On chromosome 1, pantree was run using 1 CPU core and 128 GB requested memory on an Intel Xeon Gold 5320 CPU (2.20 GHz; 257 GB RAM); it completed in 6 h 48 min and reached a peak memory usage of 34.48 GB. For comparison, vg deconstruct was run on chromosome 1 using 1 CPU core on an Intel Xeon Platinum 8468 CPU (1 TB RAM); it completed in 27 min and reached a peak memory usage of 13.74 GB. Across all chromosomes, pantree required 447 GB of peak memory and 55 hours of wall time in total (Supplementary Table 15).[[[dksdksdkd”
Comment: Definition of superbubble (Introduction, p. 2): the authors define a superbubble as "a region with a single entry node and a single exit node, such that every directed path from the entry to the exit is contained within the bubble." I believe this definition is incomplete and should be revised. In particular, it omits the property that the subgraph can be extracted without altering the topology of the remaining graph—i.e., there are no edges connecting internal nodes of the superbubble (excluding entry and exit nodes) to nodes outside the subgraph. As currently stated, the definition does not exclude the possibility of paths that traverse internal nodes without passing through the entry and exit nodes. This point is important, as it explains why vg deconstruct and vg snarls do not output interlocking bubbles, and this is one of the motivations of this work to answer this limitation.
Response: We thank the reviewer for this comment. Our intention was to give an informal summary rather than a formal definition, but we agree that the wording could be read as incomplete. We therefore clarified the sentence in the Introduction to make the topological separability property explicit. The revised text reads:
“In a directed graph, a superbubble is such a region with a single entry node and a single exit node, such that every directed path from the entry to the exit is contained within the

Referees’ reports, third round of review

Reviewer #1: The authors have addressed all my comments. I think that the manuscript is in good shape now. I have no further remarks.
