# Supplementary material for: Defining and cataloging variants in pangenome graphs
Source: Cell Genom. 2026 Aug 17;6(9):101327. doi: 10.1016/j.xgen.2026.101327 (PMC13576716; doi:10.1016/j.xgen.2026.101327)
Supplement: Supplementary file 3 [file mmc22.pdf]

Cell Genomics

Article

Defining and cataloging variants in pangenome graphs

Graphical abstract

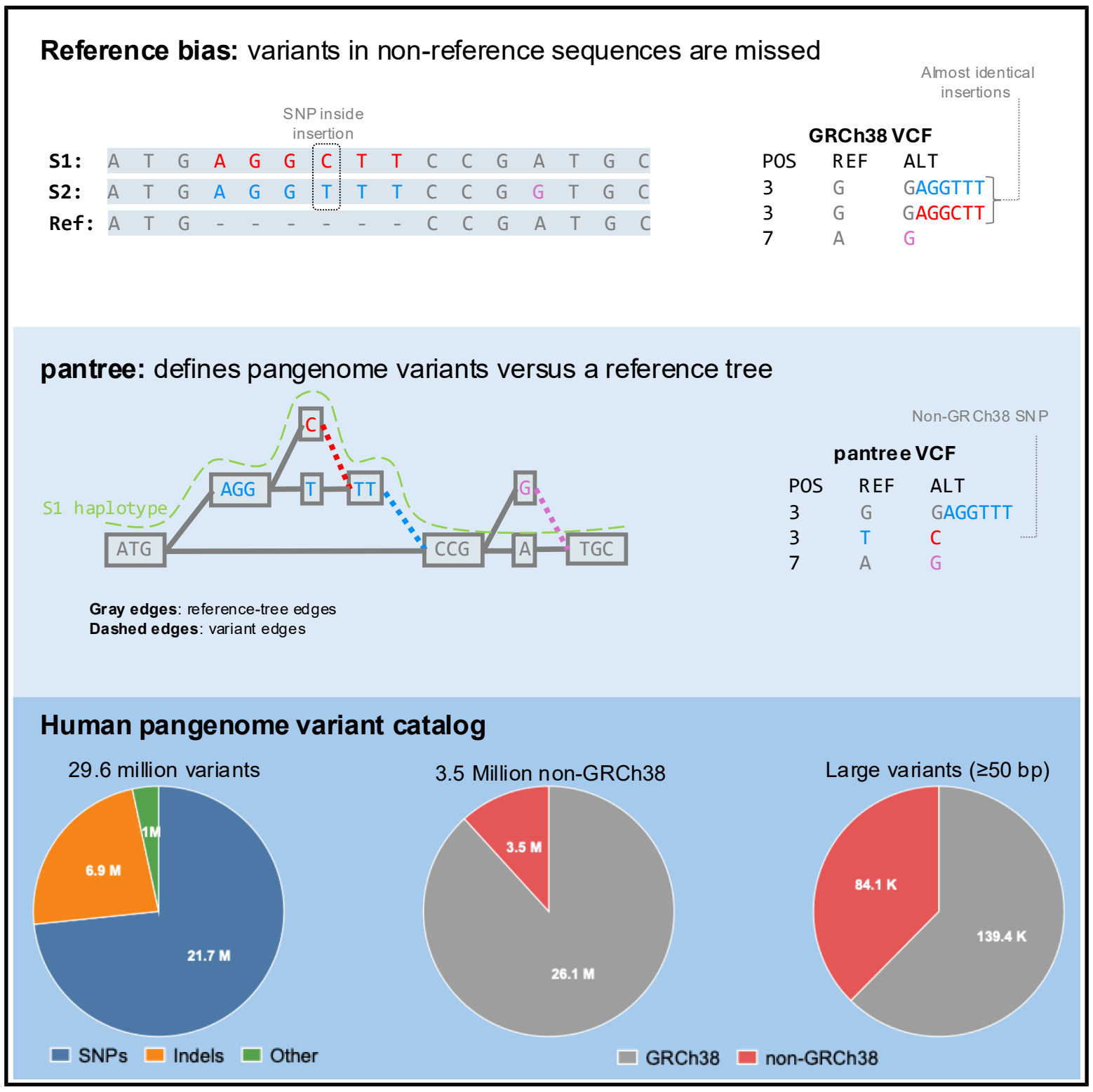

Highlights

- pantree defines pangenome variants relative to a reference tree
- We release pantree software and a genome-wide VCF catalog
- Genome-wide catalog includes 29.6 million variants and 3.5 million non-GRCh38 variants
- Non-GRCh38 variants are concentrated in complex multiallelic regions

Authors

Pouria Salehi Nowbandegani, Shenghan Zhang, Haoyang Hu, Heng Li, Luke J. O'Connor

Correspondence

pouria_salehinowbandegani@hms.harvard.edu (P.S.N.),
hli@ds.dfci.harvard.edu (H.L.),
loconnor@hms.harvard.edu (L.J.O.)

In brief

Salehi Nowbandegani et al. introduce pantree, a framework for defining variants in pangenome graphs. By choosing a reference tree and cataloging non-tree edges, pantree resolves variants embedded in non-GRCh38 sequences and produces a genome-wide VCF catalog.

Salehi Nowbandegani et al., 2026, Cell Genomics 6, 101327
September 9, 2026 © 2026 The Authors. Published by Elsevier Inc.
https://doi.org/10.1016/j.xgen.2026.101327

CellPress

Cell Genomics

CellPress
OPEN ACCESS

Article

Defining and cataloging variants in pangenome graphs

Pouria Salehi Nowbandegani,[1,2,3,4,6,*] Shenghan Zhang,[1,4] Haoyang Hu,[1,3,4] Heng Li,[1,3,5,*] and Luke J. O'Connor[1,2,5,*]
[1]Department of Biomedical Informatics, Harvard Medical School, Boston, MA, USA
[2]Program in Medical and Population Genetics, Broad Institute of MIT and Harvard, Cambridge, MA, USA
[3]Department of Data Science, Dana-Farber Cancer Institute, Boston, MA, USA
[4]These authors contributed equally
[5]These authors contributed equally
[6]Lead contact
*Correspondence: pouria_salehinowbandegani@hms.harvard.edu (P.S.N.), hli@ds.dfci.harvard.edu (H.L.), loconnor@hms.harvard.edu (L.J.O.)
https://doi.org/10.1016/j.xgen.2026.101327

SUMMARY

Structural variation causes some human haplotypes to align poorly with the linear reference genome, and this leads to "reference bias." A pangenome reference graph could ameliorate this bias by relating a sample to multiple reference assemblies. However, this approach requires a new definition of a "genetic variant." We define pangenome variants against a reference tree that includes all nodes (sequences) of the pangenome graph but only a subset of its edges; non-reference edges are variant edges. Analyzing the Minigraph-Cactus draft human pangenome reference graph, we identified 29.6 million genetic variants. 3.5 million variants (11.7%) have a reference allele that is not on GRCh38; these variants are difficult to detect without a pangenome reference and are found within tangled, multiallelic regions. We analyze the HLA-A and RHD gene regions and identify thousands of small variants entangled with several structural variants. We release the open-source pantree and a variant call format (VCF) variant catalog.

INTRODUCTION

Human genomes vary in myriad ways, from short polymorphic repeats to large-scale rearrangements. In regions with structural variation, many haplotypes align poorly with any single reference genome; two haplotypes may be similar to each other but dissimilar to the reference; for example, when they carry different alleles of a long insertion. A natural solution is to collate multiple reference assemblies, comprising a pangenome, and in 2023, the Human Pangenome Reference Consortium (HPRC) published a draft pangenome reference including 47 diploid assemblies.[1–5] The pangenome reference requires a change in perspective. A single genome is represented not as a linear sequence of alleles but rather as a walk through the pangenome graph, in which each node represents a sequence of base pairs. The notion of genomic "position" must be revisited, as many sequences are missing from the linear reference. Most fundamentally, it becomes a challenge to identify or even to define a genetic "variant."

Currently, variants in pangenome graphs are defined as different kinds of "bubbles"; loosely, these are regions where two walks split apart and then merge back together.[6,7] In a directed graph, a superbubble is such a region with a single entry node and a single exit node so that every directed path from the entry to the exit is contained within the bubble; i.e., no internal node is connected by an edge to a node outside the bubble. The bidirected generalization of a superbubble, allowing for inversions, is called a snarl.[7] Snarls can be organized into a nested hierarchy. However, a challenge is that snarls can contain many long alleles, some of them differing by only a few nucleotides despite being kilobases in length. In such regions, hierarchical decomposition succeeds for properly nested events, such as a long insertion containing several SNPs, but it cannot decompose interlocking structures, and nested sites do not in general inherit unique coordinates on the chosen linear reference path.[4,7]

Liao et al.[4] used three methods to work around these limitations. First, vg deconstruct was used to identify snarls and emit a raw variant call format (VCF) for level 0 snarls as well as nested snarls traversed by the reference allele (for example, SNPs nested inside of deletions). Second, they introduced the vcfwave pipeline, which takes the allele sequences from the raw VCF records emitted by vg deconstruct, realigns them to the linear reference, and decomposes the resulting alignments into "primitive" variants. This approach recovers small variants nested within deletions and other replacement-like alleles, but not within insertions, because the reference allele is still required to lie on the linear reference genome. Third, Liao et al.[4] also implemented a traversal-based decomposition procedure (distributed as the resolve-nested-genotypes tool) that uses the allele-traversal annotations in the raw VCF: for each raw VCF site, it compares the graph traversal of the reference allele to the traversals of the alternative alleles and extracts the implied nested

This is an open access article under the CC BY license (http://creativecommons.org/licenses/by/4.0/).

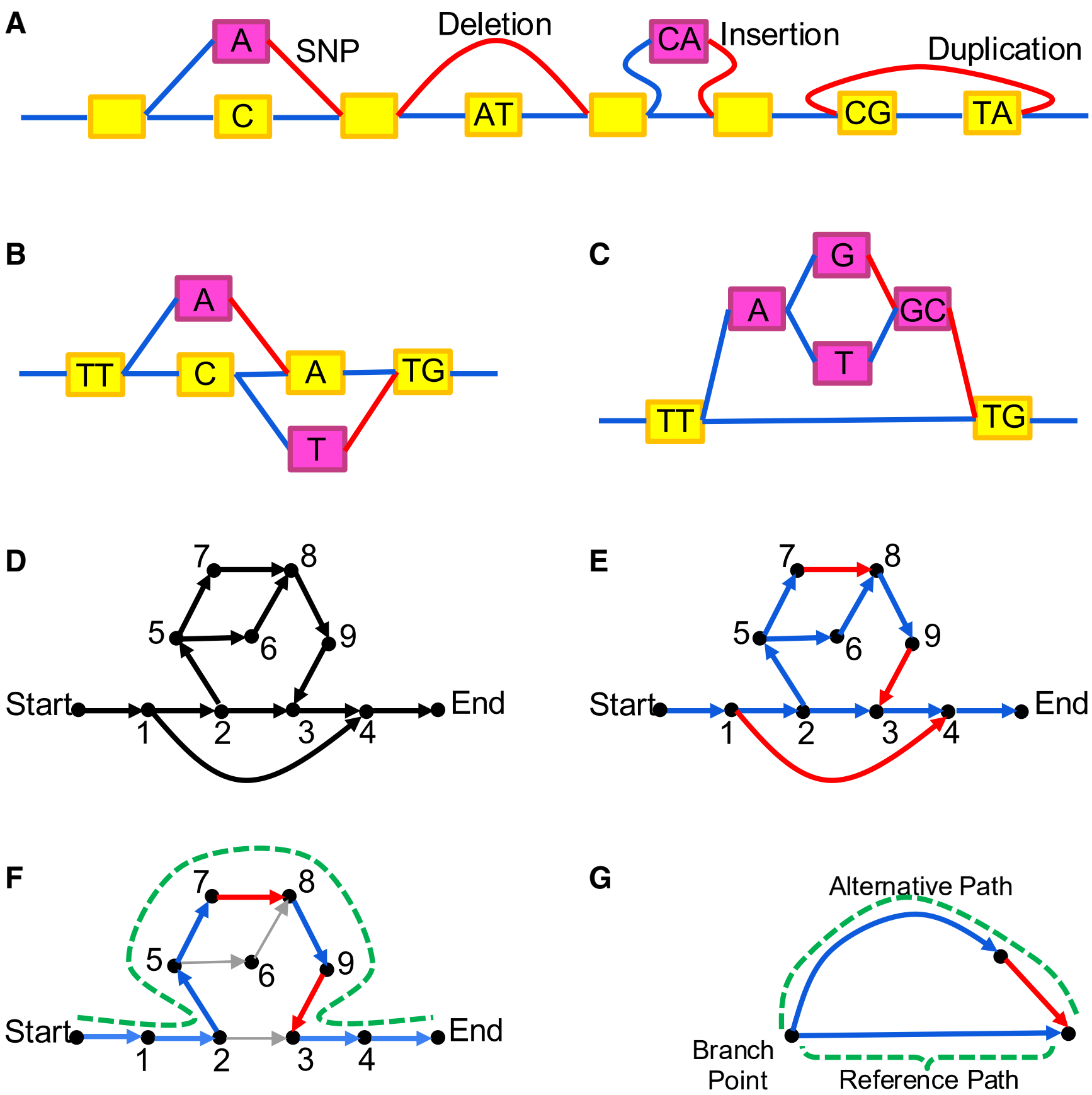

Figure 1. Definition of a pangenome variant

(A) Simple small and structural variants along a linear reference (yellow nodes): from left to right, a SNP changing C:A, a deletion removing "AT", an insertion of "CA", and a duplication of "CGTA". Pink nodes mark sequence that differs from the linear reference.

(B) A superbubble with two interlocking SNPs. The superbubble has reference allele "CA" and two alternative alleles, "AA" and "CT"; pantree finds two separate SNPs.

(C) An outer superbubble containing an insertion with a SNP on the inserted sequence together with a smaller nested superbubble. Bubble-based callers such as vg deconstruct report the outer site with reference allele "T" and alternative alleles "TATGC" and "TAGGC," whereas pantree identifies one insertion and one SNP.

(D) A toy example graph with 11 nodes.

(E) A reference tree (in blue) and corresponding variant edges (in red).

(F) A walk representing one individual haplotype (in green), with its variant edges highlighted (red).

(G) Paths corresponding to the reference and alternative alleles of a variant edge.

SNPs and insertions or deletions (indels) as separate biallelic variant records. Unlike vcfwave, which decomposes variants by realigning allele sequences back to the linear reference, this third approach decomposes them by analyzing how haplotype paths traverse the graph site itself.

Beyond the snarl/superbubble framework used in HPRC, several related bubble-like site definitions and detection methods have been proposed. In the bidirected framework introduced by Paten et al.,[7] ultrabubbles are a special class of snarls whose interior is acyclic and tip free. More recently, Billi introduced panbubbles as a bidirected generalization of superbubbles and bibubbles as a second site class for more complex configurations,[8] while pangene uses bibubbles in gene graphs to represent gene content differences rather than sequence-level allelic variation.[9] The corresponding tools implement or detect these structures in different ways: vg detects snarls,[2,7] gfatools bubble detects bubble-like structures in pangenome graphs,[10] and Billi and pangene implement the panbubble/bibubble frameworks described above. BubbleGun similarly detects bubble-like structures and related bubble chains in sequence graphs.[11] Although these methods differ in their formal site definitions, they share a common limitation for variant representation; they identify local bubble-like subgraphs but do not by themselves provide a coordinate system along a chosen reference path and therefore cannot directly produce a non-redundant, genome-wide biallelic VCF representation of all variants.

We propose to define pangenome variants against a reference tree. This tree contains all nodes of the pangenome graph but only a subset of its edges. The remaining edges, called variant edges, represent genetic variants: when some genome passes through a variant edge, it carries an alternative allele; when it reaches the same node via a reference tree edge, it carries a reference allele. With this definition, alleles are paths on the reference tree, and variants are pairs of alleles with a shared "branchpoint." Mathematically, the set of variant edges is akin to a basis for a vector space.

We developed pantree to find a reference tree for the HPRC draft pangenome and produce a variant catalog (variant edges) including many thousands of non-GRCh38 variants. We compare our variants with previously identified bubble-based approaches by HPRC, characterizing the differences between different approaches. We analyze two complex, medically relevant loci, visualizing and cataloging the variation that is present in these regions. We make our variant catalog available as a VCF file, which is compatible with widely used tools.

RESULTS

Definition of a variant

In a pangenome graph, each node represents a DNA sequence. A sample is represented as a walk, the nodes of which are concatenated. Many parts of the graph have a simple linear structure, forming a sequence of separate superbubbles (Figure 1A). Other parts contain a nested or interlocking structure (Figures 1B and 1C).

We define variants in a pangenome graph by constructing a reference tree containing a subset of the edges of the graph

(blue edges in Figure 1). Edges not in the reference tree are variant edges. Each red edge in Figure 1A represents a different type of variant; a haplotype that follows the first red edge has the "A" allele of a SNP, a haplotype that follows the second red edge has a deletion, and so on. In this example, variant edges have a one-to-one correspondence with (super)bubbles, but this is not the case in Figure 1B, where two variant edges lie within a single superbubble, or in Figure 1C, which contains one outer superbubble together with a smaller nested superbubble, whereas pantree identifies one insertion and one SNP.

Some regions can be particularly complex, especially in tandem repeats and segmental duplications. Figures 1D and 1E illustrate a complex superbubble containing three variant edges (an insertion, a deletion, and a SNP). The genotype of a walk is defined as the number of times it visits each variant edge; the green walk (Figure 1F) has genotype [0,1,1] for variant edges (1,4), (7,8), (9,3), respectively. Conversely, the genotype of a walk determines the walk because, in a tree, there exists a unique path between any two nodes. A walk that traverses exactly one variant edge (u,v) must follow the unique reference tree path from its starting node to u and then the unique reference path from v to its ending node (STAR Methods).

Variant edges have a reference allele and an alternative allele, which are defined with respect to the branchpoint of a variant edge. The branchpoint of non-inversion variant edge (u,v) is the lowest common ancestor of u and v in the reference tree (Figure 1F). The reference allele corresponds to the reference tree path from the branchpoint to v, excluding v; the alternative allele corresponds to the reference tree path from the branchpoint to u, including u (Figure 1G) (STAR Methods).

We classify variant edges into five non-overlapping categories: insertions, deletions, replacements, duplications, and inversions (STAR Methods, variant types). An edge that skips forward from an ancestor to a descendant in the reference tree is a deletion. An edge that returns backward from a descendant to an ancestor is a duplication. An edge that crosses between two different branches is either an insertion if its reference allele is empty or a replacement; SNPs are replacements whose alleles both have length one, and multiple-nucleotide polymorphisms (MNPs) are replacements whose alleles have the same non-unit length. An inversion is an edge connecting two nodes with different orientations (see below). Figure 1A illustrates a SNP, a deletion, an insertion, and a duplication.

A set of variant edges gives a basis for a vector space, where the elements of that space are differences between walks (STAR Methods). Different choices of reference trees yield different variant edges and different reference vs. alternative alleles, but any choice gives a complete description of the ways in which individual genomes differ. Moreover, some features are invariant. Node 3 in Figure 1A has two incoming edges, one of which will be chosen as the reference tree edge; the other will be a variant edge.

Because DNA is a double-stranded molecule, pangenome graphs are bidirected, respecting the symmetry between a sequence and its reverse complement (e.g., ATCC and GGAT).[12] In the STAR Methods, we provide precise definitions for a bidirected graph, a bidirected spanning tree, and a bidirected variant edge. A choice of spanning tree naturally orients each node of a bidirected graph; variant edges between nodes of different orientation are inversion edges. A simple inversion event corresponds to two different inversion edges: one deletes a DNA sequence, and the other inserts its reverse complement.

Many bioinformatics pipelines—in particular, those based on the VCF file format—require variants to have well-defined positions with respect to a linear reference genome. We include the linear reference (here, GRCh38) as a branch of the reference tree and define the position of a non-GRCh38 node u as the highest position along the linear reference so that the reference tree contains a directed path from that position to u. This definition motivates a choice of reference tree that contains GRCh38 as its main "branch" and that is constructed using a depth-first search (STAR Methods). A variant edge (u,v) has position ($pos(u)$,$pos(v)$), although this definition is modified for the VCF (discussion). This definition allows us to work locally with respect to the linear reference; within a region of interest, it suffices to consider just those variant edges whose position overlaps with the region (STAR Methods). The same is not true if variant position is defined as any single point. We also use variant positions to define haplotype missingness (STAR Methods).

Catalog of variants

We analyzed the draft human pangenome reference compiled by HPRC, in particular the genome graph inferred using Minigraph-Cactus (MC).[3] This graph contains 80,069,733 nodes, 110,938,345 edges, and 90 haplotypes, including the GRCh38 reference assembly. Each haplotype is represented by 113–566 contiguous walks, with an average of 286 walks per haplotype. Most samples in the reference panel are from the 1000 Genomes project and have African or American ancestry. We applied our software package and produced a single VCF file containing the variants we found together with the genotype of each sample (STAR Methods).

We identified 29.6 million genetic variants and annotated them as either a SNP, an insertion, a deletion, an MNP, a replacement (non-SNP and non-MNP), a duplication, or an inversion. Variants with a combined allele length of <50 bp were called "small," others "large." As expected, most variants (99.2%) were small, and most small variants (73.9%) were SNPs, with a substantial number of small insertions (10.8%) and deletions (12.1%) (Figure 2A). Most large variants were insertions (38.0%) or deletions (41.3%) (Figure 2B). Most small indels were repeat expansions or contractions (Figure 2A), most often short tandem repeats and homopolymers (Figure S1). Because of the way MC constructs the graph, most repeat expansions are annotated as insertions and not duplications; there were just 53 duplications. The alternative PGGB (Pan-Genome Graph Builder) algorithm[13] is more likely to produce a duplication. There were 860 inversion edges, almost all of them large, and these inversion edges correspond to about half as many inversion events (discussion). These numbers include chromosomes 1–22; chromosomes X and Y had similar distributions of variant types (Figures S2 and S3).

3.5 million variants (11.7%) had a reference allele that is not on the linear reference genome, and we refer to these as non-GRCh38 variants. About half were SNPs, others mostly indels (Figure 2C). These variants are difficult to detect without a

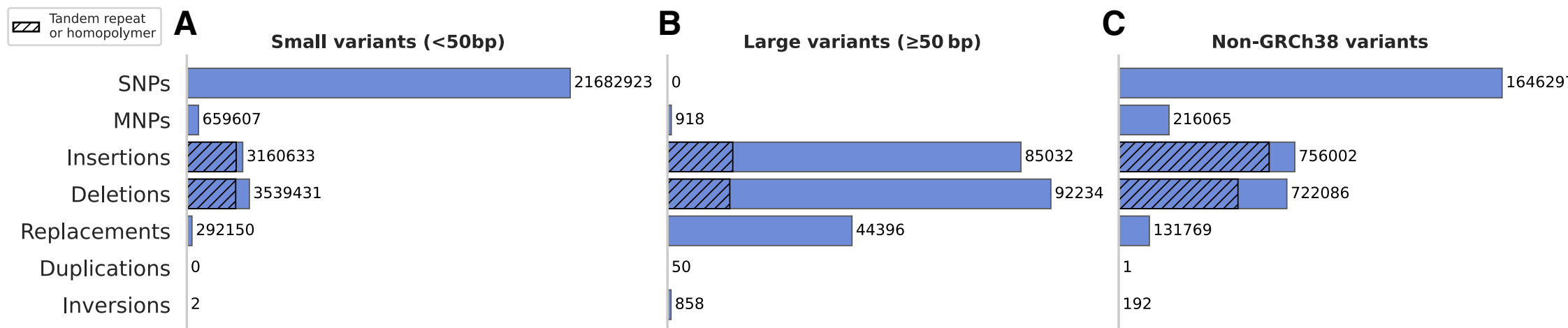

Figure 2. Summary of variants in the HPRC draft pangenome

(A) Number of small variants (<50 bp combined allele length) across seven non-overlapping variant types. The hatched area indicates the number of variants that cause a repeat expansion or contraction.

(B) Number of large variants (≥50 bp).

(C) Number of non-GRCh38 variants whose reference allele is not on the GRCh38 genome.

pangenome reference. They include 21,109 variants, mostly indels, that are both common and large (Figure S4).

To provide a concrete example of how pantree's ability to call variation inside non-reference sequence is useful in practice, we asked whether non-GRCh38 variants identified by pantree fall in exons and coding sequences of annotated genes. We analyzed the subset of non-GRCh38 variants with available CHM13 haplotype positions in the VCF generated from the haplotype-contiguous depth-first search (DFS) tree on the year 2 HPRC MC graph. These haplotype positions provide coordinates along the CHM13 walk and can therefore be intersected with gene annotations on the T2T-CHM13 assembly. Across all autosomes and chromosome X, 3,453,855 non-GRCh38 variants had available CHM13 positions; of these, 99,167 (2.9%) overlapped exons, and 14,562 (0.4%) overlapped coding sequences, spanning 1,182 genes (Table S1; chromosome-level summary in Table S2). These genes include many of major biomedical interest, including HLA class I and II genes (e.g., HLA-A, HLA-B, HLA-C, HLA-DRB1, and HLA-DQB1), immunoglobulin heavy- and light-chain variable genes (IGHV, IGKV, and IGLV families), killer cell immunoglobulin-like receptors (e.g., KIR2DL1 and KIR3DL1), and genes implicated in neurological disease, including HTT, ATXN1, ATXN2, and ATXN3 (Table S3). Many of these genes lie in structurally complex, highly polymorphic loci, such as the major histocompatibility complex (MHC), immunoglobulin loci, and mucin gene clusters, where a non-reference sequence is expected to harbor functionally important variation. For comparison, among on-GRCh38 variants with available CHM13 haplotype positions in the same VCF, 1,899,837 (4.3%) overlapped exons, and 358,973 (0.8%) overlapped coding sequences, spanning 19,893 genes (Table S1). Thus, pantree identifies thousands of coding non-GRCh38 variants in medically relevant genes that are not recoverable by methods such as vcfwave, which require the reference allele to lie on the linear reference genome.

Validation and methods comparison

We compared the SNPs detected by pantree with the approaches previously used by HPRC (STAR Methods). Recall that, in the HPRC workflow, vg deconstruct identifies level 0 snarls and emits a "raw VCF" that also includes nested snarls traversed by the reference allele. Another approach is to post-process those raw VCF records with vcfwave, which realigns each alternative allele sequence to the linear reference and decomposes the resulting alignments into primitive variants.

In "easy" regions of the genome, where every haplotype closely tracks the linear reference genome, pantree and the raw VCF found approximately the same set of SNPs. In segmental duplications, our approach detected 46.1% more SNPs (2,313,929 vs. 1,584,132), and in other difficult regions, it detected 43.4% more SNPs (Figure S5). The SNPs that we find are a superset of those in the raw VCF except for exactly two raw-VCF-specific SNPs (these arise due to assembly gaps; Figures S6 and S7). All SNPs absent from the raw VCF are nested within larger snarl sites, and 62.0% of these SNPs are non-GRCh38 SNPs.

Some SNPs absent from the raw VCF, but only those that are on the reference genome, can be recovered using vcfwave. This approach cannot recover non-GRCh38 SNPs because it relies on pairwise alignment with the linear reference genome (STAR Methods). We find 1,896,587 SNPs that are missed by vcfwave, most of which are non-GRCh38 (Figure 3A). Non-vcfwave SNPs are modestly enriched at lower allele frequencies, but many of them (23.8%) are common (minor allele count $\geq$ 5 of 90) (Figure 3B). vcfwave variants can be inconsistent with the pangenome graph, and indeed, 239,375 SNPs are vcfwave specific (Figure 3A); these arise because vcfwave may produce a different alignment compared with the graph construction algorithm (Figure S8). Chromosome X had a similar proportion of non-GRCh38 SNPs and non-vcfwave SNPs as compared with the autosomes (Figure S2); chromosome Y was enriched for non-GRCh38 and non-vcfwave SNPs (Figure S3).

Comparing non-SNP variants between methods is more challenging because for non-SNPs, the same sequence change can sometimes be represented in multiple ways. For example, if the sequence ATAT mutates to AT, then this deletion has three equivalent encodings (delete first AT, delete TA, delete second AT). This is particularly a challenge when comparing against vcfwave, which uses a different alignment compared with the graph, so we restricted our comparison to the HPRC raw VCF. We selected raw-VCF sites in the chromosome 20 raw VCF for which GRCh38 and CHM13 have different alleles, and we classified these as biallelic or multiallelic and as an insertion, deletion, SNP, or other complex event. For biallelic variants, we required an exact match of chromosome, position, REF, and ALT between the raw and pantree VCFs. Under this strict criterion,

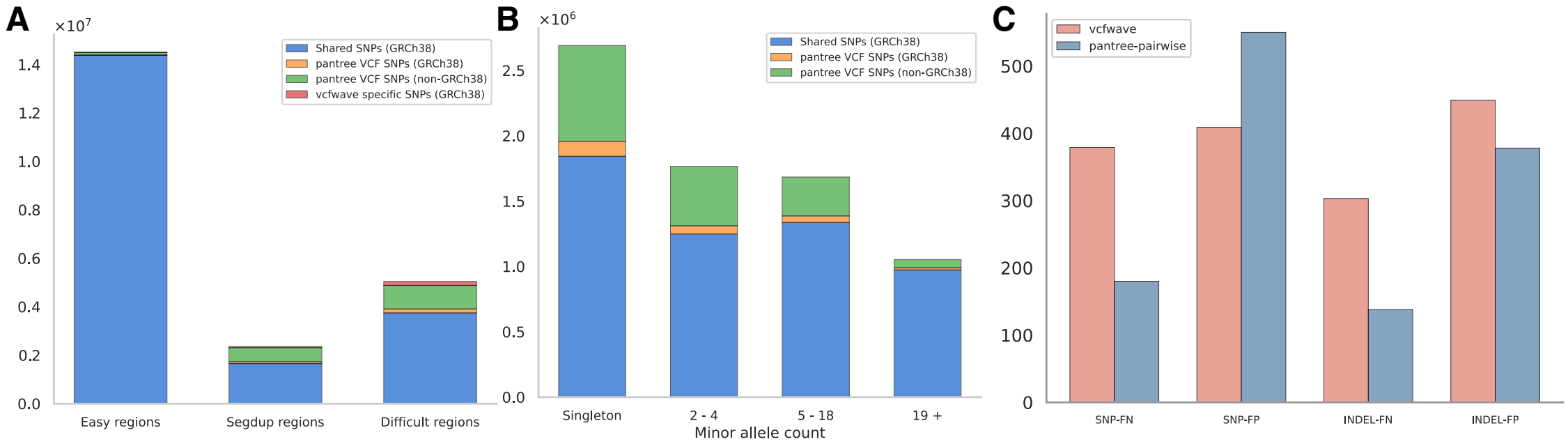

Figure 3. Comparison of pantree with vcfwave

(A) Overlap of SNPs with those reported by vcfwave across three non-overlapping genome annotations.

(B) Minor allele count (the minimum of the reference and alternative allele counts) of 90, stratified by overlap with vcfwave.

(C) Small-variant benchmarking against an external truth set using RTG vcfeval, showing SNP and indel false negatives (FNs) and false positives (FPs) for vcfwave and the pantree-pairwise post-processed call set.

For numerical results, see Tables S4, S7, S8, S9, S10, and S11.

pantree recovered all biallelic insertions (1,600/1,600), all biallelic deletions (1,826/1,826), and 84.9% of biallelic "other" variants (399/470). The biallelic "other" mismatches reflect representational differences rather than missing events: some superbubbles contain several internal walks between the same endpoints but only two sample haplotype paths, so they appear as single biallelic records in the raw VCF, whereas pantree represents the same region as multiple, finer-grained variant edges—especially in interlocking configurations, as discussed below.

We benchmarked pantree using RTG vcfeval,[14] which compares variants called in a VCF file against a manually curated "ground truth" VCF for the heavily sequenced HG002 sample. This sample is absent from the year 1 HPRC graph but present in the year 2 graph, so we ran pantree on chromosome 21 of that graph. RTG vcfeval expects the input VCF to enumerate pairwise difference between HG002 and GRCh38, which is not exactly what pantree does; instead, pantree enumerates difference against the reference tree, only one branch of which is GRCh38. For this reason, we developed a post-processing tool (STAR Methods) that converts a pantree VCF into a VCF that enumerates pairwise difference against the reference genome. This tool, similar to vcfwave, operates on the VCF alone. It could be useful when interrogating one particular haplotype. We compared pantree, pantree-pairwise, the raw VCF, and vcfwave; of these four methods, pantree-pairwise and vcfwave are the two whose objective approximates that of the benchmark. pantree-pairwise performed best overall, particularly for indels (Figure 3C); as expected, pantree and raw performed poorly due to mismatch with the benchmark (Table S4).

To validate that pantree calls non-GRCh38 variants reliably, we re-ran vg deconstruct with a different linear reference genome. We selected the CHM13#0#chr20 contig (~26.3 Mb, spanning ~40% of the chromosome) and ran vg deconstruct with this contig as the reference path to obtain a raw VCF. We then identified pantree SNPs where CHM13 has an ALT allele and matched them to SNPs from the vg deconstruct raw VCF by comparing the end node of each bubble with swapped REF/ALT alleles (to account for the different reference orientations). In this region, pantree recovered 100% (24,570/24,570) of vg deconstruct SNPs while calling an additional 2,996 SNPs, consistent with what we found when using GRCh38 as the reference. Notably, we did not re-run pantree with a different reference genome; this shows that pantree is equally able to identify variants that are not on the linear reference (Table S5).

To assess whether pantree variants are reproducible across independently constructed pangenome graphs, we compared pantree variants between the year 1 (46 samples) and year 2 (233 samples) HPRC MC graphs for chromosome 21. Both graphs contain the CHM13 T2T assembly, and we ran pantree with the haplotype-contiguous DFS method (STAR Methods) using GRCh38 and CHM13 as priority samples, producing haplotype positions (HPs) along both walks. Since CHM13 uses the same assembly in both graphs, its HP provides a stable physical coordinate across graphs; after correcting for a contig-start offset between the two GFA files, CHM13 positions match exactly for on-GRCh38 SNPs. We matched variants between graphs using a two-coordinate key, the GRCh38 position (POS) and the CHM13 HP, requiring both to match exactly with no position window. Because a graph with more samples may decompose a variant more finely (for example, representing a year 1 deletion as a deletion plus a nested SNP in year 2), we used subsequence matching; a year 1 variant was considered recovered if a year 2 variant at the same (POS, HP) had an allele that contained, or was contained within, the year 1 allele. Restricting to variants on the dominant CHM13 contig, 99% of on-GRCh38 year 1 variants were recovered in year 2 by subsequence matching, including 100% of SNPs, 97% of deletions, and 93% of insertions. We then considered the subset of non-GRCh38 variants with an available CHM13 HP that places them on this same dominant contig. Of 7,754 such non-GRCh38 variants, 90% were recovered in the year 2 graph, including 89% of SNPs, 92% of deletions, and 90% of insertions (Table S6). Thus, non-GRCh38 variants are nearly as reproducible across graph versions as on-GRCh38 variants, with the modest gap plausibly explained by finer decomposition in the larger year 2 graph. These results support the conclusion that most non-GRCh38 variants represent stable sequence differences rather than artifacts of a particular graph build, and they

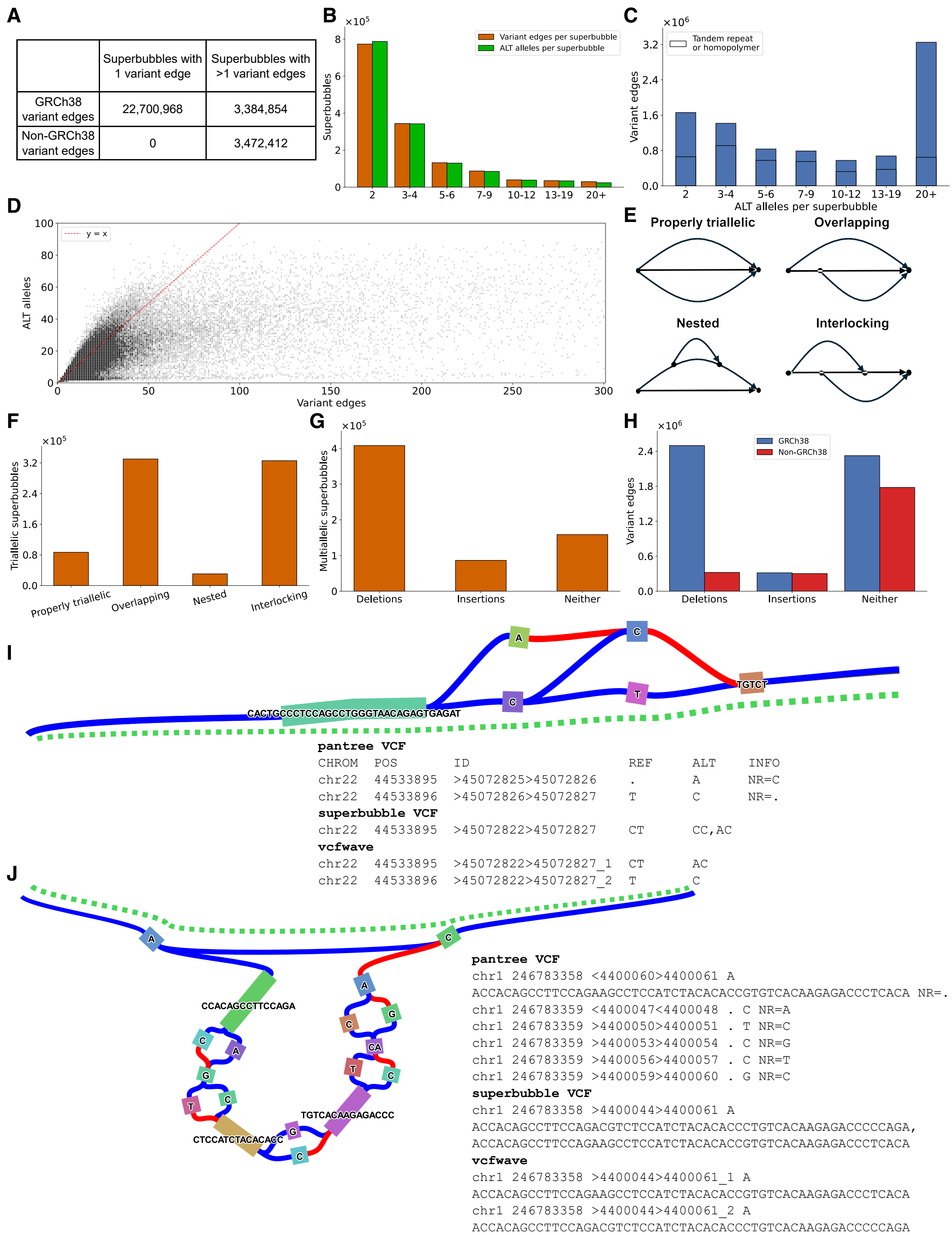

Figure 4. Variant content of multiallelic superbubbles

All analyses in this figure are restricted to level 0 superbubbles.

(A) Number of GRCh38 and non-GRCh38 variant edges contained in biallelic (1 variant edge) versus multiallelic (>1 variant edge) superbubbles. All non-GRCh38 variant edges occur in multiallelic superbubbles.

(B) The number of multiallelic superbubbles containing different numbers of variant edges (orange) and alternative alleles (green).

(C) The number of variant edges contained in superbubbles with different numbers of alternative alleles.

(D) Number of alternative alleles vs. variant edges per superbubble.

(legend continued on next page)

demonstrate a practical procedure for comparing variants across independently constructed graphs using HPs as cross-graph coordinates. Detailed genome-wide numerical summaries of variant classes, repeat annotations, genomic region annotations, SNP-method comparisons, and frequency-stratified comparisons are provided in Tables S7, S8, S9, S10, and S11.

MC uses a linear reference genome, here GRCh38, to initialize graph construction. Regions that are missing from GRCh38 entirely, including centromeres and acrocentric arms, are also missing from the MC GRCh38 graph. We analyzed the HPRC MC CHM13 graph, which is initialized at the telomere-to-telomere CHM13 assembly, using CHM13 as the pantree reference genome. This graph contains 14.7% more variants than the GRCh38 graph, and the difference is mostly explained by an increased number of variants within centromeres, subtelomeres, and acrocentric arms (Figure S9A). These regions are highly enriched for off-linear-reference variants; the number of non-CHM13 variants in the CHM13 graph is 42.4% greater than the number of non-GRCh38 variants in the GRCh38 graph, almost entirely because of these regions (Figure S9B). Graph construction in these regions is difficult, and some of these variants could be due to alignment errors. In the GRCh38 graph, most non-GRCh38 variants are not contained in these regions.

Multiallelic superbubbles

All non-GRCh38 variant edges are contained within a level 0 superbubble (STAR Methods) containing at least two variant edges (Figure 4A). These multi-variant superbubbles are usually multiallelic; we sought to characterize multiallelic, multi-variant superbubbles. Both count distributions—the number of alleles per superbubble and the number of variant edges—were heavy tailed (Figure 4B). There were 23,694 superbubbles containing at least 20 alternative alleles, and these contained 11.1% of variant edges (Figure 4C). Allele count and variant count are correlated, but there is no exact correspondence (Figure 4D) except that superbubbles with more than one alternative allele always contain more than one variant edge.

We classified multiallelic superbubbles in three ways. First, we classified triallelic, two-variant superbubbles as either "properly triallelic," "overlapping," "nested," or "interlocking" (Figure 4E; STAR Methods). Most triallelic superbubbles are either interlocking (42.1%) or overlapping (42.7%) (Figure 4F). Variants in these bubbles are mostly SNPs (47.0%) or indels (48.4%) (Table S12), and they account for 3.5% of non-GRCh38 variants. The predominant interlocking and overlapping categories are difficult to decompose using existing approaches. Properly triallelic superbubbles do not need to be decomposed, as their three alleles have no partial similarity. Nested superbubbles can be decomposed using an existing hierarchical approach, which is to remove the endpoints of a superbubble and identify superbubbles within the connected components of the remaining subgraph, but this approach cannot decompose overlapping or interlocking superbubbles. vcfwave sometimes resolves overlapping or interlocking variants, but its behavior depends on which of the three alleles is the reference allele; for example, when two SNPs are interlocking and one of them is off the linear reference, *vcfwave* does find two variants, but only one of them is a SNP. It also misses a handful of GRCh38 SNPs due to a rare edge case (Figure S10).

Second, we classified multiallelic superbubbles with more than three alleles as insertions (13.2%), deletions (62.5%), or neither (24.3%) (Figure 4G). An insertion superbubble contains a reference tree edge from the beginning to the end of the superbubble; a deletion superbubble contains a beginning-to-end variant edge. The larger number of deletion superbubbles is mostly due to an ascertainment effect. If a superbubble contains several alleles, just one of which is empty, then the empty allele usually has a frequency of less than 0.5, and the linear reference usually has the non-empty allele. If the linear reference has the non-empty allele, then the superbubble is classified as a deletion (Figure S11). Most non-GRCh38 variants (74.0%) are found outside of insertion and deletion superbubbles, although insertions (which comprise non-GRCh38 sequence exclusively) are enriched, containing 12.6% of non-GRCh38 variants in total (Figure 4H). Representative triallelic and insertion superbubbles are shown in Figures 4I and 4J.

Third, in order to understand where non-GRCh38 variants are located, we focused on highly multiallelic superbubbles with at least 10 alternative alleles. There are only 95,632 such bubbles (Figure 4B), but each one may contain dozens of variants (Figure 4D). These contain 65.9% of non-GRCh38 variants. They are likely to contain long insertions without being classified strictly as an insertion superbubble; 2.1% of them contain an insertion of at least 1 kb, and these contain 37.6% of non-GRCh38 variants, an average of 646 per superbubble (Table S13). Numerical summaries of multiallelic superbubble counts, triallelic categories, and insertion/deletion/non-insertion/deletion classes are provided in Tables S14, S15, S16, and S17.

Complex regions

We investigated two specific regions of the genome that are known to harbor complex patterns of genetic variation: the region around the HLA-A gene, which contains HLA-A and several copy number-variable pseudogenes, and the RHD region, which contains an inversion overlapping the RHD gene (STAR Methods). HLA alleles have well-studied effects on autoimmunity, and RHD alleles largely determine Rh blood group.[4] In both regions, the pangenome graph is too complex to be visualized in full; to simplify it, we pruned variant edges except for

(E) Schematic of each possible triallelic superbubble.
(F) Counts of each possible triallelic superbubble.
(G) Number of more-than-triallelic superbubbles classified as insertions, deletions, or neither.
(H) Number of GRCh38 and non-GRCh38 variants in more-than-triallelic superbubbles classified as insertions, deletions, or neither.
(I) Example of a triallelic superbubble, with excerpts from three VCF files. The columns are chromosome, position, node identifiers, reference allele, and alternative allele or alleles, respectively.
(J) Example of a multiallelic superbubble classified as an insertion, with VCF excerpts.
For numerical results, see Tables S12, S13, S14, S15, S16, and S17.

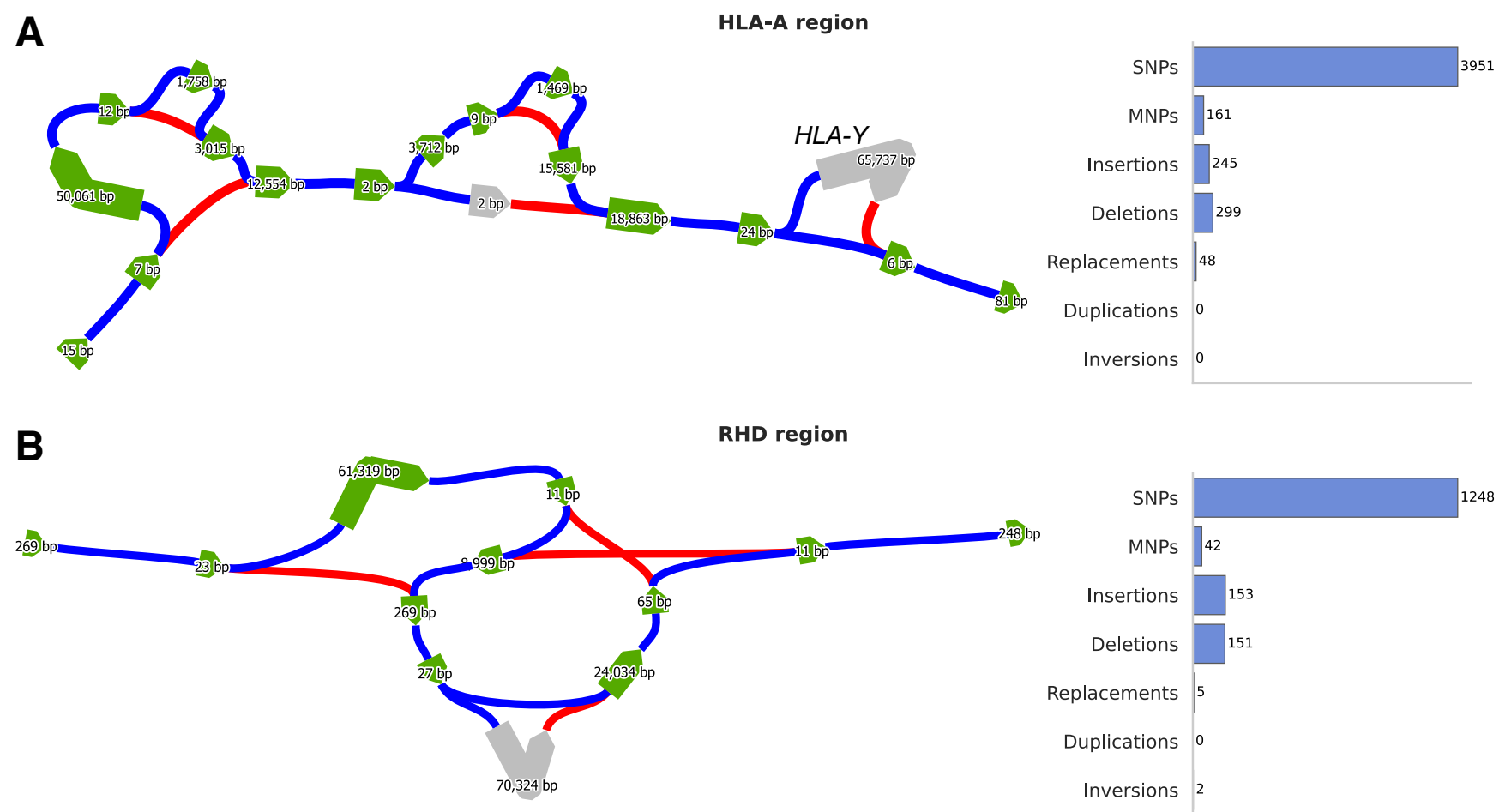

Figure 5. Analysis of two complex regions
Blue and red edges indicate reference tree paths and paths containing variant edges, respectively. Green and gray nodes indicate GRCh38 and non-GRCh38 sequences, respectively.
(A) Simplified graph of the HLA-A region and a summary of variants identified. The location of the HLA-Y pseudogene is indicated.
(B) Simplified graph of the RHD region and a summary of variants identified.

those with very long alleles (combined length >1 kb), deleted “tips” not contained in a cycle, and contracted non-branching paths (STAR Methods). The graph was visualized as a Bandage plot.[15] This simplification is enabled by our variant-edge definition; by first discarding small variant edges (combined allele length <50 bp) and then contracting edges that every haplotype is forced to traverse (obligatory linear segments), we can collapse fine-scale variation while preserving the set of pantree variants that remain in the graph.

The HLA-A region contains 4,704 total variants in just 3 superbubbles, which are visually apparent in the simplified graph (Figure 5A). The three superbubbles contain 75, 68, and 24 alleles with average allele lengths of 53, 20, and 66, respectively; however, most of these alleles are highly similar, differing only by small variants. In the simplified graph, there are only three structurally different walks through the first two superbubbles and two through the third. These correspond to large structural variants. The first bubble is a deletion of a 55-kb region containing a nested 1-kb deletion, the second is a near-deletion of a 21-kb region also containing a nested 1-kb deletion, and the third is a 66-kb insertion. The 66-kb insertion, which contains the HLA-Y pseudogene, contains 98 variants and 24 unique alleles; all of these variants are non-GRCh38 and are therefore missed by vcfwave.

The RHD region forms a single superbubble containing 86 unique alleles (average length: 8.4 kb); we identify 1601 variant edges, 78% of which are SNPs. Only one haplotype carries the inversion, which is 33 kb long. With our definition, an inversion is represented by a pair of inversion edges, both of which are carried by that haplotype (discussion). The region also harbors a common 70-kb deletion of the RHD gene (alternative allele count: 27) and a common 70-kb insertion that duplicates the RHD gene (alternative allele count: 6). These variants differ in length by 5 bp, and they are spaced 296 bp apart. The insertion is nested inside of the inverted region; the deletion is interlocking with it, spanning one of the two inversion break points (Figure 5B).

This kind of analysis can be done manually, but it is greatly streamlined by our approach, which makes it easy to enumerate structural variants and visualize their relationships. Fine-scaled variation can be removed for visualization and then mapped back to the simplified graph.

DISCUSSION

The human pangenome is intended to address the problem of reference bias, that no single reference genome is an adequate point of comparison for all other genomes. Indeed, the pangenome graph contains 129.3 megabases of non-GRCh38 genetic sequence,[4] but existing definitions of a genetic variant in the pangenome still rely heavily on the linear reference. They are unable to resolve differences, even SNPs, between different non-reference sequences (i.e., non-GRCh38 variants). We addressed this challenge by replacing the linear reference genome, which lacks non-GRCh38 sequences, with a reference tree containing all nodes and sequences in the graph. With this approach, we found 3.5 million genetic variants that are not located on the linear reference genome.

The linear reference genome, our reference tree, and the pangenome reference graph are all referred to as a “reference.” We suggest that the reference tree is logically parallel to the reference genome, whereas the pangenome itself is logically parallel to a reference panel like 1000 Genomes. A reference tree, like a reference genome, is a point of comparison; a pangenome, like a reference panel, contains both reference and alternative sequences. Any haplotype in a reference panel can be reconstructed by starting from the reference genome and replacing reference with alternative alleles; likewise, a haplotype in a pangenome graph can be reconstructed by starting from the reference tree (STAR Methods).

A natural question, having discovered variants in a pangenome graph, is how to genotype these variants in a new sample not belonging to the graph. pantree is not designed for this problem, but some existing methods provide partial solutions. vg giraffe is a pangenome read mapper[16] that could be used for variant genotyping; one possible but suboptimal approach would be to align reads to the graph and then genotype pantree variant edges from the resulting read support. PanGenie, by contrast, is a k-mer-based genotyper[17] that uses a phased variant VCF for genotyping without a graph. In principle, one could imagine modifying PanGenie to take pantree VCF as input.

We published our variant catalog in a VCF file in order to streamline downstream applications, but this choice required certain compromises or workarounds. The REF column in a VCF is required to match the reference genome at the specified position, so the reference allele of a non-GRCh38 variant must be written in a different field. Moreover, in a VCF, insertions

and deletions have a letter prepended to both their reference and alternative alleles; we follow this convention for variants on the linear reference but not for variants off of it to avoid breaking the reference allele rule. The POS column of a VCF is a single integer, whereas we define variant position as a tuple, one of which must be chosen for the POS column. POS encodes the position of the first letter of each allele, which is offset by one from the way that we would choose to define it, which is the last letter before each allele begins (this approach more gracefully handles insertions). In each of these cases, we added custom INFO fields to the VCF file so that no information is lost, but users of our VCF files should be aware of these workarounds, especially when analyzing non-GRCh38 variants. For example, users should not misinterpret non-GRCh38 variants as being insertions just because their REF column is empty.

Inversions are particularly challenging to analyze in a pangenome graph. Defining inversions requires orienting the nodes of the bidirected graph (MC assigns arbitrary orientations). A choice of reference tree implies a choice of orientations; the same variant edge could be an inversion with respect to one reference tree and not with respect to another. Whereas most variant edges likely correspond to a single mutational event, an inversion event corresponds to a pair of variant edges with opposing orientations: from the positive to the negative orientation and back. Sometimes inversion edges can be paired up in this way but not always, in particular when the inverted region overlaps with an assembly gap. Therefore, we represent inversion edges individually without assuming perfect pairing.

Although we suggest that superbubbles should no longer be used to define genetic variants, they are useful to highlight complex regions. Simply counting either variant edges or alleles per superbubble is a useful heuristic for the complexity of a genomic region, with complex regions often having many alleles and variants. Indeed, a large fraction of non-GRCh38 variants are contained in highly multiallelic superbubbles. When a variant is nominated for special scrutiny, for any reason, a reasonable starting point would be to analyze its enclosing superbubble; one could enumerate variant edges in that superbubble, visualize it (perhaps after simplification), or even inspect its alleles by eye.

Whereas the GRCh38 reference genome forms a simple path—never visiting any node twice—in the MC graph, it does not do so in the pangenome graph inferred using PGGB, and other reference genomes (e.g., CHM13) do not generally form a simple path in either graph. Our framework can, in principle, handle linear reference genomes with repeated nodes (STAR Methods), but doing so introduces two related complications. First, nodes that lie on reference genome cycles no longer have a single position, so multiple reference positions become superimposed in the graph. Second, when repeated or merged nodes represent sequence aligning to multiple distinct loci on GRCh38, as can occur in PGGB graphs, a pantree variant edge may simultaneously capture differences among orthologous haplotypes and divergence between paralogous copies of the reference. The interpretation of the variant edge changes fundamentally from an inter-genome difference to an intra-genome difference. For this reason, we restrict our analyses to MC graphs in which GRCh38 follows an acyclic path; our software can be applied to PGGB graphs, but we do not recommend doing so.

Limitations of the study

Just as the choice of reference genome always affects the way that genetic variants are represented, including their REF vs. ALT alleles and their positions - the choice of reference tree affects the variant edges that are found. The total number of variants is fixed, but not the variants themselves, and also not the number of SNPs or indels. What does remain constant is the difference between any two haplotypes and the fact that their difference is encoded uniquely as a combination of variant edges. Our reference trees are constructed using depth-first traversal while prioritizing edges used by many haplotypes. A different possible choice is to prioritize certain haplotypes when constructing the tree; this yields a haplotype-contiguous reference tree in which the highest-priority haplotypes tend to follow long, uninterrupted segments on the tree, changing which edges are treated as variants (STAR Methods). We compared the variants found using each approach; although individual variants differ, the number of variants and the distribution of variant types is highly similar (Figure S12). It is straightforward to translate between different reference trees: if a sample was genotyped against one reference tree, then its walk can simply be reconstructed, and it can then be genotyped against any other reference tree. This change-of-basis operation is implemented in our software package. A related but different question is how to compare two pangenome graphs as opposed to two reference trees for the same graph. In our chromosome 21 analysis, we show that this can be done in practice when the same haplotype is present in both graphs; the pair consisting of the GRCh38 position and the HP along a shared CHM13 walk provides a practical cross-graph coordinate. Using this approach, most variants, including most non-GRCh38 variants with available CHM13 HPs on the largest CHM13 contig, were recovered between the year 1 and year 2 HPRC graphs. This does not eliminate representation dependence, but it shows that cross-graph comparison is feasible in practice.

pantree suffers from two potential sources of error, both related to its input data. First, although the HPRC assemblies have undergone extensive validation, residual assembly errors would produce spurious variant edges. Second, MC[3] can make alignment errors, especially in tandem repeats and segmental duplications. At short tandem repeat loci, recurrent mutation makes it impossible to reconstruct a unique homology structure between repeat copies, so some aligned sequences may be only approximately homologous. In such regions, pantree variants remain a valid and useful representation of the differences between haplotypes as encoded by the graph, but they should not be interpreted as a record of individual mutation events in the genealogical history of orthologous sequences. Importantly, both error sources would affect all variant representations including vg deconstruct and vcfwave; they are not specific to pantree.

The functional consequences of the genetic variants that we found are unknown. Discovering variants is a prerequisite for characterizing their effects, and historically, these efforts have been separate stages; for example, the International HapMap

Project first built a public map of common human variation and then enabled downstream association studies using that resource. At a minimum, we do know that complex loci can be medically relevant, and pantree identifies novel variation at such loci (HLA-A and RHD). In addition, at the genome-wide level, we found that 14,562 non-GRCh38 variants with available CHM13 HPs overlap coding sequence in 1,182 genes, including HLA, immunoglobulin, KIR, and repeat-expansion disease genes, illustrating a practical use case for pantree's ability to resolve variation within non-reference sequence. Understanding the effects of such variants at these and other loci will require experimental evidence and, possibly, genetic association studies.

RESOURCE AVAILABILITY

Lead contact

Requests for further information and resources should be directed to and will be fulfilled by the lead contact, Pouria Salehi Nowbandegani (pouria_salehinowbandegani@hms.harvard.edu).

Materials availability

This study did not generate new unique reagents.

Data and code availability

- Genome-wide VCF files generated in this study are publicly available at Zenodo (https://zenodo.org/records/15374896, DOI: https://doi.org/10.5281/zenodo.15374896). These files include full-genome VCFs for autosomes and chromosomes X and Y, with per-individual genotypes, generated using our variant-edge framework on both the GRCh38-aligned HPRC MC v.1.1 pangenome graph and the CHM13-aligned HPRC MC graph.
- The pangenome graphs used in this study were constructed by the HPRC. The HPRC MC v.1.1 GRCh38 graph is available at https://s3-us-west-2.amazonaws.com/human-pangenomics/pangenomes/freeze/freeze1/minigraph-cactus/hprc-v1.1-mc-grch38/hprc-v1.1-mc-grch38.gfa.gz. The corresponding HPRC MC v.1.1 CHM13 graph is available at https://s3-us-west-2.amazonaws.com/human-pangenomics/pangenomes/freeze/freeze1/minigraph-cactus/hprc-v1.1-mc-chm13/hprc-v1.1-mc-chm13.gfa.gz. The year 2 HPRC MC v.2.1 graph used for cross-graph comparison and CHM13 haplotype position analyses is available through the HPRC Data Explorer alignments portal (https://data.humanpangenome.org/alignments). Superbubble annotations were obtained from .snarls files precomputed by the vg toolkit for the HPRC MC v.1.1 graph.
- To stratify genomic regions by complexity, we used BED files defining easy, segmental duplication, and hard regions. The GIAB v.3.5 easy-region BED file is available at https://ftp-trace.ncbi.nlm.nih.gov/giab/ftp/release/genome-stratifications/v3.5/GRCh38@all/Union/GRCh38_notinalldifficultregions.bed.gz, and the UCSC segmental duplication annotation is available at https://hgdownload.soe.ucsc.edu/goldenPath/hg38/database/genomicSuperDups.txt.gz. For the genome-wide coding region analysis, we used the NCBI RefSeq v.110 annotation for the T2T-CHM13 v.2.0 assembly, downloaded from the CHM13 project repository at https://github.com/marbl/CHM13. From this resource, we extracted gene, exon, and CDS annotations for autosomes and sex chromosomes and mapped NCBI accession identifiers to standard chromosome names before overlap analysis.
- The pantree software package, version 0.2.0, implements reference-tree construction, variant-edge definition, and VCF generation. pantree is archived at Zenodo (DOI: https://doi.org/10.5281/zenodo.21363929) and is also available at https://github.com/oclb/pantree. Analysis scripts used in this study are available at https://github.com/ShenghanZhang1123/graph_var_analysis and DOI: https://doi.org/10.5281/zenodo.21363929.
- In addition to our own code, we used several publicly available open-source tools. The vg toolkit (https://github.com/vgteam/vg) was used to extract superbubble information from .snarls files and to analyze superbubbles within the pangenome graph. gfatools (https://github.com/lh3/gfatools) was used for graph manipulation and format conversion. Bandage (https://github.com/rrwick/Bandage) was used to visualize pangenome graph structures. RTG vcfeval (https://github.com/RealTimeGenomics/rtg-tools) was used for benchmarking against the GIAB HG002 Q100 v.1.1 small-variant benchmark.

ACKNOWLEDGMENTS

L.J.O. acknowledges funding under NIH-NIGMS award R35-GM155278 and Simons Foundation SFARI award 1009802LO. H.L. acknowledges funding under NIH-NHGRI awards R01-HG010040, U01-HG013748, and U41-HG010972.

AUTHOR CONTRIBUTIONS

P.S.N., S.Z., H.H., H.L., and L.J.O. developed the methods. P.S.N., S.Z., and H.H. performed the experiments. P.S.N., S.Z., H.H., H.L., and L.J.O. wrote the paper. H.L. and L.J.O. supervised the research.

DECLARATION OF INTERESTS

The authors declare no competing interests.

STAR★METHODS

Detailed methods are provided in the online version of this paper and include the following:

- KEY RESOURCES TABLE
- EXPERIMENTAL MODEL AND STUDY PARTICIPANT DETAILS
- METHOD DETAILS
  - *pantree* pipeline
  - Mathematical definition of pangenome variants
  - Reference tree and variant edges
  - Variant types
  - Repeats
  - Missingness
  - Simplified graph
  - Handling non-path linear references
  - Comparing variants across graphs
  - *pantree* VCF specification
  - HPRC variant catalog
  - Validation and methods comparison
  - Multiallelic superbubbles
  - Complex regions
- QUANTIFICATION AND STATISTICAL ANALYSIS
- ADDITIONAL RESOURCES

SUPPLEMENTAL INFORMATION

Supplemental information can be found online at https://doi.org/10.1016/j.xgen.2026.101327.

Received: August 6, 2025
Revised: March 7, 2026
Accepted: July 14, 2026
Published: August 17, 2026

REFERENCES

1. Eizenga, J.M., Novak, A.M., Sibbesen, J.A., Heumos, S., Ghaffaari, A., Hickey, G., Chang, X., Seaman, J.D., Rounthwaite, R., Ebler, J., et al.

CellPress
OPEN ACCESS

(2020). Pangenome Graphs. Annu. Rev. Genom. Hum. Genet. *21*, 139–162. https://doi.org/10.1146/annurev-genom-120219-080406.

2. Garrison, E., Sirén, J., Novak, A.M., Hickey, G., Eizenga, J.M., Dawson, E.T., Jones, W., Garg, S., Markello, C., Lin, M.F., et al. (2018). Variation graph toolkit improves read mapping by representing genetic variation in the reference. Nat. Biotechnol. *36*, 875–879. https://doi.org/10.1038/nbt.4227.
3. Hickey, G., Monlong, J., Ebler, J., Novak, A.M., Eizenga, J.M., Gao, Y., Abel, H.J., Antonacci-Fulton, L.L., Asri, M., Baid, G., et al. (2024). Pangenome graph construction from genome alignments with Minigraph-Cactus. Nat. Biotechnol. *42*, 663–673. https://doi.org/10.1038/s41587-023-01793-w.
4. Liao, W.-W., Asri, M., Ebler, J., Doerr, D., Haukness, M., Hickey, G., Lu, S., Lucas, J.K., Monlong, J., Abel, H.J., et al. (2023). A draft human pangenome reference. Nature *617*, 312–324. https://doi.org/10.1038/s41586-023-05896-x.
5. Sedlazeck, F.J., Rescheneder, P., Smolka, M., Fang, H., Nattestad, M., Von Haeseler, A., and Schatz, M.C. (2018). Accurate detection of complex structural variations using single-molecule sequencing. Nat. Methods *15*, 461–468. https://doi.org/10.1038/s41592-018-0001-7.
6. Onodera, T., Sadakane, K., and Shibuya, T. (2013). Detecting Superbubbles in Assembly Graphs. In Algorithms in Bioinformatics Lecture Notes in Computer Science, A. Darling and J. Stoye, eds. (Springer Berlin Heidelberg), pp. 338–348. https://doi.org/10.1007/978-3-642-40453-5_26.
7. Paten, B., Eizenga, J.M., Rosen, Y.M., Novak, A.M., Garrison, E., and Hickey, G. (2018). Superbubbles, Ultrabubbles, and Cacti. J. Comput. Biol. *25*, 649–663. https://doi.org/10.1089/cmb.2017.0251.
8. Bhat, S.G., Mahajan, D., and Jain, C. (2025). Billi: Provably Accurate and Scalable Bubble Detection in Pangenome Graphs. Preprint at bioRxiv. https://doi.org/10.1101/2025.11.21.689636.
9. Li, H., Marin, M., and Farhat, M.R. (2024). Exploring gene content with pangene graphs. Bioinformatics *40*, btae456. https://doi.org/10.1093/bioinformatics/btae456.
10. Li, H., Feng, X., and Chu, C. (2020). The design and construction of reference pangenome graphs with minigraph. Genome Biol. *21*, 265. https://doi.org/10.1186/s13059-020-02168-z.
11. Dabbaghie, F., Ebler, J., and Marschall, T. (2022). BubbleGun: enumerating bubbles and superbubbles in genome graphs. Bioinformatics *38*, 4217–4219. https://doi.org/10.1093/bioinformatics/btac448.
12. Kececioglu, J.D., and Myers, E.W. (1995). Combinatorial algorithms for DNA sequence assembly. Algorithmica *13*, 7–51. https://doi.org/10.1007/BF01188580.
13. Garrison, E., Guarracino, A., Heumos, S., Villani, F., Bao, Z., Tattini, L., Hagmann, J., Vorbrugg, S., Marco-Sola, S., Kubica, C., et al. (2024). Building pangenome graphs. Nat. Methods *21*, 2008–2012. https://doi.org/10.1038/s41592-024-02430-3.
14. Cleary, J.G., Braithwaite, R., Gaastra, K., Hilbush, B.S., Inglis, S., Irvine, S.A., Jackson, A., Littin, R., Rathod, M., Ware, D., et al. (2015). Comparing Variant Call Files for Performance Benchmarking of Next-Generation Sequencing Variant Calling Pipelines. Preprint at bioRxiv. https://doi.org/10.1101/023754.
15. Wick, R.R., Schultz, M.B., Zobel, J., and Holt, K.E. (2015). Bandage: interactive visualization of *de novo* genome assemblies. Bioinformatics *31*, 3350–3352. https://doi.org/10.1093/bioinformatics/btv383.
16. Sirén, J., Monlong, J., Chang, X., Novak, A.M., Eizenga, J.M., Markello, C., Sibbesen, J.A., Hickey, G., Chang, P.-C., Carroll, A., et al. (2021). Pangenomics enables genotyping of known structural variants in 5202 diverse genomes. Science *374*, abg8871. https://doi.org/10.1126/science.abg8871.
17. Ebler, J., Ebert, P., Clarke, W.E., Rausch, T., Audano, P.A., Houwaart, T., Mao, Y., Korbel, J.O., Eichler, E.E., Zody, M.C., et al. (2022). Pangenome-based genome inference allows efficient and accurate genotyping across a wide spectrum of variant classes. Nat. Genet. *54*, 518–525. https://doi.org/10.1038/s41588-022-01043-w.
18. Hansen, N.F., Dwarshuis, N., Ji, H.J., Rhie, A., Loucks, H., Logsdon, G.A., Vollger, M.R., Storer, J.M., Kim, J., Adam, E., et al. (2025). A complete diploid human genome benchmark for personalized genomics. Preprint at bioRxiv. https://doi.org/10.1101/2025.09.21.677443.
19. Dwarshuis, N., Kalra, D., McDaniel, J., Sanio, P., Alvarez Jerez, P., Jadhav, B., Huang, W., Mondal, R., Busby, B., Olson, N.D., et al. (2024). The GIAB genomic stratifications resource for human reference genomes. Nat. Commun. *15*, 9029. https://doi.org/10.1038/s41467-024-53260-y.
20. Goldfarb, T., Kodali, V.K., Pujar, S., Brover, V., Robbertse, B., Farrell, C.M., Oh, D.-H., Astashyn, A., Ermolaeva, O., Haddad, D., et al. (2025). NCBI RefSeq: reference sequence standards through 25 years of curation and annotation. Nucleic Acids Res. *53*, D243–D257. https://doi.org/10.1093/nar/gkae1038.
21. West, D.B. (2001). Introduction to Graph Theory, Second Edition (Prentice Hall).
22. Tarjan, R.E. (1979). Applications of Path Compression on Balanced Trees. J. ACM *26*, 690–715. https://doi.org/10.1145/322154.322161.

STAR★METHODS

KEY RESOURCES TABLE

REAGENT or RESOURCE	SOURCE	IDENTIFIER
Deposited data		
pantree VCF catalogs	this manuscript	Zenodo: https://doi.org/10.5281/zenodo.15374896
HPRC Minigraph-Cactus v1.1 GRCh38 graph	HPRC; Hickey et al.[3]; Liao et al.[4]	hprc-v1.1-mc-grch38.gfa.gz
HPRC Minigraph-Cactus v1.1 CHM13 graph	HPRC; Hickey et al.[3]; Liao et al.[4]	hprc-v1.1-mc-chm13.gfa.gz
HPRC Minigraph-Cactus v2.1 graph	HPRC; Hickey et al.[3]; Liao et al.[4]	HPRC Data Explorer: alignments
GIAB HG002 Q100 v1.1 benchmark	GIAB; Hansen et al.[18]	GIAB HG002 Q100 v1.1
Genome stratification BED files	GIAB; Dwarshuis et al.[19]	GIAB v3.5 easy regions/UCSC genomicSuperDups
CHM13 RefSeq v110 annotation	NCBI RefSeq; Goldfarb et al.[20]	CHM13 project repository
Software and algorithms		
pantree v0.2.0	this manuscript	Zenodo: DOI: https://doi.org/10.5281/zenodo.21363929; GitHub: https://github.com/oclb/pantree
vg toolkit	Garrison et al.[2]	GitHub: https://github.com/vgteam/vg
gfatools	Li et al.[10]	GitHub: https://github.com/lh3/gfatools
Bandage	Wick et al.[15]	GitHub: https://github.com/rrwick/Bandage
RTG Tools/vcfeval	Cleary et al.[14]	GitHub: https://github.com/RealTimeGenomics/rtg-tools
vcfwave	Liao et al.[4]	GitHub: https://github.com/vcflib/vcflib (vcfwave)

EXPERIMENTAL MODEL AND STUDY PARTICIPANT DETAILS

This study did not involve newly recruited human participants, experimental animals, cell lines, or other experimental models. Analyses used publicly available de-identified human genome assemblies, pangenome graphs, annotations, and benchmark datasets generated by HPRC, GIAB, NCBI, UCSC, and related public resources. No new sample allocation, randomization, blinding, or sample-size estimation was performed.

METHOD DETAILS

pantree pipeline

pantree takes as input a bidirected pangenome graph in GFA format, with nodes representing DNA sequences, edges encoding adjacencies, and W-lines specifying haplotype walks for each assembly. A designated linear reference genome (here GRCh38) is represented as one or more walks in the same graph. The main outputs are (i) a set of variant edges, defined relative to a bidirected spanning tree (the reference tree), and (ii) a VCF catalog in which each variant edge is assigned a position with respect to GRCh38, a pair of alleles (reference and alternative), a variant type, and sample-level genotypes.

Conceptually, the algorithm proceeds in three stages. First, we preprocess the graph by extracting chromosome-specific subgraphs, adding artificial termini to anchor disconnected components. Second, we construct a reference tree: a spanning tree of the bidirected graph that includes the entire GRCh38 path as a branch and orients all nodes. Third, we identify variant edges as those edges that are present in the graph but not in the reference tree, classify them into variant categories (insertions, deletions, replacements, duplications, and inversions), assign them positions along the linear reference, and compute reference/alternative allele counts and genotypes across haplotypes (see also "VCF specification").

Weighted depth-first reference tree

Our reference tree is built by a depth-first traversal that prioritizes high frequency edges. We start the traversal on the GRCh38 path for the chromosome of interest and require that the GRCh38 walk is fully embedded as a simple path in the tree. At each step, when

the traversal is at a node with multiple outgoing edges that would not create a cycle, we rank candidate edges by the number of haplotype walks that use that edge. The traversal first follows the unused edge with the largest haplotype count. This greedy heuristic tends to place high-frequency alleles on the reference tree and low-frequency alleles as variant edges.

Variant positions, alleles, and types

The linear reference genome enters the construction in two ways. First, the GRCh38 path is forced to be a branch of the reference tree, which ensures that every position on GRCh38 can be reached by a unique path in the tree. Second, we use this path to define positions for all nodes and variant edges (see results, "definition of a variant"). The position of a non-GRCh38 node is the highest GRCh38 position from which the reference tree contains a directed path to that node, and a variant edge inherits its position from its endpoints; this definition lets us work locally in GRCh38 coordinates while still allowing alleles to traverse non-reference sequence. For each variant edge, we then define a reference and an alternative allele as the two paths on the reference tree from the branchpoint to the edge endpoints, and classify the edge as an insertion, deletion, replacement, duplication, or inversion based on its orientation and its relationship to the tree (see results, "definition of a variant," **and** STAR Methods, "reference and alternative alleles" **and** "variant types").

Exclusion of terminal variant edges

To construct the reference tree, we introduced four terminus nodes (two binodes) to anchor disconnected regions (see STAR Methods, "constructing the reference tree"). Variant edges involving these termini are called terminal edges, and they are a convenient mechanism to track the start and end of each walk. Terminal edges mostly do not represent biological variation, but rather the presence of alignment gaps, and they were excluded from both our published VCFs and our variant count statistics. More precisely, for a variant edge (u,v), if either the branchpoint of u and v or the node v is one of the terminus nodes, then (u,v) is discarded.

Reference and alternative allele count

Each sample in the GFA file is represented as a collection of walks. The reference allele count for a variant is defined as the number of times a walk visits the last edge of corresponding reference path (see STAR Methods, "reference and alternative alleles") in the reference tree. The alternative allele count is defined as the number of times a walk traverses the variant edge.

Degenerate alleles

To maintain clarity in variant representation, we excluded exactly degenerate alleles (i.e., variants with identical REF and ALT alleles) from the VCF and downstream analyses.

Mathematical definition of pangenome variants

This subsection presents the mathematical and algorithmic foundations of our framework for defining genetic variants in pangenome graphs. The input is a bidirected graph constructed from a collection of walks, each representing a sample haplotype. Bidirected graphs naturally capture the double-stranded nature of DNA, allowing traversal from either orientation at each node. The central mathematical question is: given such a graph, can we define a minimal set of features that uniquely encodes all pairwise differences between walks? We formalize this in terms of symmetric differences and show that a set of variant edges, non-tree edges with respect to a reference tree, serves as a basis for all such differences. This subsection details our definitions, algorithms, and theoretical guarantees, including walk reconstruction, variant positioning, and variant edge classifications.

Pangenomes as bidirected graphs

Pangenome graphs are naturally modeled using bidirected graphs due to the strandedness of DNA, which requires representing both directions of sequence traversal. Throughout this work, we define a *bidirected graph* $B = (V,E,C)$ via its *directed representation*:

- V is the set of nodes,
- $E \subseteqq V \times V$ is the set of directed edges,
- $C: V \rightarrow V$ is a fixed-point-free involution called the complement function, satisfying $C(v) = v^c$, $(v^c)^c = v$, and $v \neq v^c$ for all $v \in V$,
- For every edge $e = (u,v) \in E$, its complement is defined as $e^c := (v^c,u^c) \in E$, so every edge appears in a pair with its directed complement.

Using these criteria, a bidirected graph can be represented as a directed graph.

The *bidirected representation* of B is the quotient structure $(V/C, E/C)$:

- A *binode* is an unordered pair $\{u,u^c\} \in V/C$,
- A *biedge* is an unordered pair $\{e,e^c\} \in E/C$.

This abstraction collapses each node with its complement and each edge with its reverse complement. For visualization purposes, we depict a binode as a double-headed arrow, where each side represents a direction of traversal. Figure S13A illustrates the bidirected representation: each binode is shown as a double-headed arrow, and biedges connect the heads or tails depending on the direction of entry and exit. In this figure, traversing from the left side to the right side of a binode corresponds to reading the forward strand sequence, while traversing from right to left corresponds to the reverse complement. Figures S13B and S13C show the directed representation of the same bidirected graph, where each binode is "unfolded" into a pair of nodes v^+ and v^-, and each biedge becomes a pair of directed edges. In this representation, forward and reverse traversals correspond to complementary nodes.

These two representations are mathematically equivalent and are used interchangeably depending on context.

A *walk* between binodes $\{v_1, v_1^c\}$ and $\{v_n, v_n^c\}$ is defined as a pair of node sequences:

$$(v_1, \ldots, v_n)\text{and}(v_n^c, \ldots, v_1^c),$$

such that each pair $(v_i,v_{i+1})\in E$ for $1 \leq i<n$. A walk is called a *path* if it does not repeat any binode: that is, $\{v_i, v_i^c\} = \{v_j, v_j^c\}$ implies $i = j$.

A bidirected graph is a *rooted tree* with root $\{r_1, r_1^c\}$ if for either r_1 or r_1^c, say r, and for all binodes $\{v_1, v_1^c\}$, there exists a unique directed path from r to either v_1 or v_1^c. Such a bidirected graph is *oriented*: its nodes V can be partitioned into two components with no edges between them, V^+ and V^-, such that if $v\in V^+$ then $v^c\in V^-$ (see "orienting nodes within each binode").

A bidirected graph $S = (V_s,E_s,C)$ is a *subgraph* of a bidirected graph $B = (V,E,C)$ if S is a bidirected graph and $V_s\subseteq V$ and $E_s\subseteq E$ i.e., V_s and E_s are closed under complement i.e., if $v\in V_s$ then $v^c\in V_s$ and if $e\in E_s$ then $e^c\in E_s$. A subgraph S is called a *spanning subgraph* if $V_s = V$. A *rooted spanning tree* of a bidirected graph is a spanning subgraph that is a rooted tree.

Reference tree and variant edges

Given the pangenome graph B and a rooted spanning tree T, called a *reference tree*, we define *variant edges* as the biedges of B that are not in T. Let T be a reference tree of B, then T induces two directed trees in the directed representation of B called *half-trees* such that one of them has edges only from V^+ to V^+, denoted by T^+, and the other one only has edges from V^- to V^-, denoted by T^-.

The representative of a variant biedge $\{e,e^c\}$ is either e or e^c. For consistency, if $\{e,e^c\} = \{(u^+,v^+),(u^-,v^-)\}$ we pick (u^+,v^+) as the representative. If a variant edge is between V^+ and V^-, it is an *inversion* edge. Note that if an inversion edge e goes from V^+ to V^-, then e^c also goes from V^+ to V^-, and similarly for an inversion edge from V^- to V^+, its complement also goes from V^- to V^+. For inversion edges, we randomly choose the representative.

The *genotype* of an individual, represented as a collection of disjoint walks through the graph, is the number of times that the walks visits each variant edge. We use the notation $x_e(W)$ for the number of times that walk W visits biedge e.

Given the sequence of variant edges visited by walk W, as well as its starting and ending nodes, W can be reconstructed uniquely because there is at most one path between any two nodes in the reference tree, in particular from the ending point of one variant edge to the starting point of the next one. Moreover, given the genotype of a walk – which tracks variant edge visit counts, but not their order – it is possible to reconstruct the number of times that it visits every biedge.

Theorem 1. Given a bidirected pangenome graph B, its reference tree T, and the starting and ending binodes of a walk W, the genotype $x(W) = \{x_e(W)|\text{variant edges } e\}$ determines W up to the order of cycles.

Before proving Theorem 1, we state and prove the following lemma.

Lemma 2. *Let B be a bidirected graph with a reference tree T, which induces the partition $V = V^+\cup V^-$. Let W,W^c be a bidirected walk in B. Let v_0,u_0 be the starting and ending nodes of W, respectively, and let the variant edges visited by W be (u_1,v_1), …,(u_n,v_n). For $i = 0$, …,n, call v_i a source if $v_i\in V^+$, and otherwise call v_i^c a sink; if $u_i\in V^+$ call it a sink, otherwise call u_i^c a source. Let* sources(W) *and* sinks(W) *denote the sequences of sources an sinks. For a node $v\in V^+$, let $D(v)\subset V^+$ be its reach set in T. Then the number of times that W visits the binode $\{v,v^c\}$ is*: $|D(v)\cap\text{sinks}(W)|-|D(v)\cap\text{sources}(W)|$.

Proof. We decompose W into a sequence S_0, …,S_n of directed paths on T, where S_i proceeds from the ith source node to the ith sink node. Each path in this sequence starts at a source node, ends at a sink node, and remains within V^+. Each path is a subsequence either of W or of W^c.

If S_i does not visit v then either:

- It starts and ends outside $D(v)$ (non-descendant to non-descendant), or
- It starts and ends within $D(v)$ (descendant to descendant),

but it cannot go from a descendant to a non-descendant, and if it goes from a non-descendant to a descendant then it would visit v. Therefore, the number of paths in S that visit v equals the number of transitions into $D(v)$:

$$|D(v)\cap\text{sinks}(W)| - |D(v)\cap\text{sources}(W)|.$$

Moreover, the number of paths that visit v is equal to the number of times that W (or W^c) visits either v or v^c.

Proof of Theorem 1. The visit count formula in Lemma 2 is invariant when the order of sources and sinks is shuffled; any two walks with the same multiplicity of sources and sinks visit a binode b the same number of times. The multiplicity of sources and sinks can be computed from the genotype of a walk, so the genotype determines the visit count of b. It also determines the number of times each tree edge is traversed. The only ambiguity is within cycles, i.e., in the relative order of subpaths that begin and end at the same binode.

Thus, the genotype $x(W)$ determines the walk W up to the order of cycles. □

To demonstrate the practical application of Theorem 1, we now present Algorithm 3, which efficiently constructs the bidirected walk W from the genotype $x(W)$.

Algorithm 3. The algorithm reconstructs the walk using the endpoints of the walk and the genotype information:

1. **Determine Sources and Sinks:** Sources and sinks are determined based on the variant edges. For each visit to a variant edge (u,v), if u is a positive node, it is added to the sinks; if it is negative, its complement is added to the sources. Similarly, if v is positive, it is added to the sources; if it is negative, its complement is added to the sinks.
2. ***Adjust for Inversions*****:** *The start and end nodes of the walk are added to the sources or sinks depending on the number of inversions. If the number of inversions is even, the start node is added to the sources and the end node to the sinks. If the number of inversions is odd, additional adjustments are made: if there is one more + to − switch, both endpoints are added to the sinks; if there is one more − to + switch, both endpoints are added to the sources. After doing this, there are an equal number of sources and sinks (otherwise, the genotype does not correspond to a valid walk).*
3. **Reconstruction of the Walk:** The reconstruction proceeds by traversing the positive-orientation reference tree in the opposite direction of the tree edges. Starting from each sink, the algorithm walks upward along the tree until reaching a source, recording each edge traversed along the way. This process avoids searching multiple branches as every node has at most one predecessor, but any number of descendants. The number of times each edge is visited is stored. When a source is reached, its count is decremented (a source is used up when its count reaches 0). This process is repeated for all sinks, ensuring all sources are matched, thus reconstructing the walk up to the order of cycles.
4. **Validation of the Genotype:** This algorithm also detects whether the genotype is valid: if it ever reaches the root, and the root no longer belongs to the set of sources, then the genotype corresponds to no single walk.

Constructing the reference tree

Suppose the bidirected graph $B(V,E,C)$ is given. Before finding the reference tree, we pre-specify the direction of each node in positive and negative classes, denoted V^+ and V^- respectively (see "orienting nodes within each binode") meaning that if $v \in V^+$, then $v^c \in V^-$, and vice versa. To ensure that B has a rooted spanning tree, we add two special binodes to B, called the start-terminus and the end-terminus, denoted by ST and ET. We also add the biedge $((ST^+,v^+),(v^-,ST^-))$ if $v^+ \in V^+$ either has in-degree zero in B or is the starting node of a walk. Recall that B is constructed from a collection of walks, where each sample haplotype consists of a collection of walks. Similarly, for a binode (v^+,v^-), if $v^- \in V^-$ either has in-degree zero or is the end of a walk, we add the biedge $((ET^-,v^-),(v^+,ET^+))$. After adding these nodes, there exists a directed spanning tree of the positive subgraph with root ST^+. This tree along with its complement on the negative subgraph forms a spanning rooted tree for the bidirected graph B. To construct this graph, we use a DFS traversal on the positive subgraph initiated at ST^+. This tree and its complement are our reference tree.

If the pangenome graph includes the linear reference as a path, with no repeated binodes, then we can ensure that nodes connected via the linear reference have the same direction. We can initialize our DFS traversal at this path. This is the case for the Minigraph-Cactus graph, but not the PGGB graph. It is desirable to include the linear reference as the primary branch of the reference tree because it allows us to define genomic position (see "the position of variants along the linear reference").

There exist many possible choices of reference tree, none of which is the best; Theorem 1 holds for any choice of reference tree. In practice, we use DFS reference trees obtained by choosing an order in which outgoing edges are explored during the traversal of the positive subgraph. We describe two constructions: a weighted DFS tree, which greedily favors edges used by many haplotype walks, and a haplotype-contiguous DFS tree, which instead prioritizes staying on selected haplotypes for as long as possible.

Definition 4 (Weighted DFS Tree). Let $B = (V,E,C)$ be an oriented bidirected graph whose positive nodes form the directed subgraph $B^+ = (V^+,E^+)$. Let $w : E^+ \to \mathbb{Z}_{\geq 0}$ assign to each directed edge its edge frequency, i.e., the number of haplotype walks that traverse that edge. A weighted DFS tree is a rooted spanning tree T^+ of B^+ obtained by a depth-first search starting at ST^+ such that whenever the search is at a node with multiple unvisited outgoing neighbors, it next follows an outgoing edge of maximum weight $w(e)$ (with ties broken by any fixed deterministic rule). The corresponding reference tree is the bidirected tree $T = T^+ \cup T^-$, where T^- is the complement of T^+.

Definition 5 (Haplotype-Contiguous DFS Tree). Let $B = (V,E,C)$ be an oriented bidirected graph, and let $H_1,H_2, \ldots,H_m$ be a fixed priority ordering of distinguished haplotype walks (for example GRCh38, CHM13, HG002, …). A haplotype-contiguous DFS tree is a rooted spanning tree T^+ of B^+ obtained by a depth-first search starting at ST^+ such that, whenever the search is at a node with multiple unvisited outgoing neighbors, it first chooses an outgoing edge that continues the highest-priority haplotype in the list whose contiguous extension is still available from the current node. If no such prioritized haplotype determines the next step, the search falls back to the weighted DFS rule and chooses an outgoing edge of maximum frequency. The corresponding reference tree is again $T = T^+ \cup T^-$, where T^- is the complement of T^+.

The weighted DFS tree favors high-frequency alleles globally, whereas the haplotype-contiguous DFS tree favors keeping selected haplotypes on long uninterrupted branches of the reference tree.

The position of variants along the linear reference

In practice, it is important that the variants have a well-defined position with respect to the linear reference genome. We assign positions to variants by making an appropriate choice of reference tree. In particular, we choose a depth-first search (DFS) tree whose first branch is the linear reference genome. If a node u is not on the linear reference, it is assigned the largest position of any node on the linear reference that can reach u in T. Equivalently, this is the largest position of any node on the linear reference that can reach u in B without visiting a back-edge with respect to T.

The position of a binode $\{u,u^c\}$ is defined as the linear reference position of u (or u^c) if either one lies on L. Otherwise, it is the position of the node x on L that serves as the ancestor of u (or descendant of u^c) in T. The position of a variant edge $e = (u,v)$ in B, denoted by

pos(e), is defined as the interval [a,b], where a is the position of u and b is the position of v. This position assignment is illustrated in Figure S14.

Theorem 6. *For an inversion-free bidirected graph*, *if the position of e is* [a,b], *then* $x_e(w)$ *is determined by the variant edges whose position overlaps* [a,b]: $\{x_\eta(w) | \text{variant edges}\, \eta\, \text{s.t. pos}(\eta) \cap [a,b] \neq \emptyset\}$

Proof. By the definition of position, each node not on the linear reference inherits its position from the highest ancestor on the linear reference reachable via T. Since there is a path on T from u to v, by the definition of position $pos(u) \leq pos(v)$.

Therefore, if a path from a to b exists in T and pos(a)$\neq$pos(b), then the path must pass through the ancestor of b that lies on the linear reference and has position pos(b).

Suppose B is an inversion-free bidirected graph with a reference tree T. Consider a variant edge (u,v) and a binode b in B. Utilizing Lemma 2, we show that if pos(b)$\notin$[pos(u),pos(v)], then for any walk W,

$$|D(b) \cap \text{sinks}(w)| - |D(b) \cap \text{sources}(w)|$$

remains invariant.

Without loss of generality, we may assume that pos(u)$\leq$pos(v) since we only work within T for the remainder of the proof. There are two cases:

Case 1: pos(b)<pos(u).

- **Subcase 1.1:** $u \in D(b)$. In this case, there is a path within T from b to u. This path must pass through the ancestor of u within the linear reference whose position is equal to pos(u). Since pos(u)$\leq$pos(v) and (u,v) is not an inversion, there is a path from b to the ancestor of v within the linear reference whose position is equal to pos(v), and therefore there is a path within T from b to v. Hence, $v \in D(b)$ and $|D(b) \cap \text{sinks}(w)| - |D(b) \cap \text{sources}(w)|$ remains unchanged.
- **Subcase 1.2:** $u \notin D(b)$. In this case, there is no path within T from b to the ancestor of u within the linear reference whose position is equal to pos(u). Therefore, since pos(u)$\leq$pos(v) and (u,v) is not an inversion, there is no path from b to the ancestor of v within the linear reference whose position is equal to pos(v), and therefore there is no path within T from b to v. Hence, $v \notin D(b)$ and $|D(b) \cap \text{sinks}(w)| - |D(b) \cap \text{sources}(w)|$ remains unchanged.

Case 2: pos(v)$\leq$pos(b). In this case, both u and v are not in $D(b)$, and therefore $|D(b) \cap \text{sinks}(w)| - |D(b) \cap \text{sources}(w)|$ remains unchanged. □

Theorem 6 shows that our definition of position not only has the linear nature of the genome, but also allows us to work locally: in order to reconstruct any walk through a locus, it suffices to consider those variants whose positions intersect with that locus.

The inversion-free assumption in Theorem 6 is essential. The following counterexample illustrates this point: Let $e=(u^+,v^+)$ be an edge with position [a,b]. Consider two inversion edges, $e_1 = (u_1^-, v_1^+)$ and $e_2 = (u_2^+, v_2^-)$, with positions [a_1,b_1] and [a_2,b_2], respectively. Suppose that $b_1<a$ and $b<a_2$, so the positions of e_1 and e_2 do not intersect the position interval of e. Let W_1 be a walk that visits e once, without visiting e_1 or e_2. Now let W_2 be a walk with the same genotype as W_1 except it visits both e_1 and e_2 exactly once. In this case, W_2 visits e twice, despite having the same variant edge counts as W_1 within the interval [a,b].

Orienting nodes within each binode

The reference is constructed via depth-first search on the positive-direction subgraph of the bidirected pangenome graph. Before doing so, we assign directions to each node of the graph.

A subgraph P of a bidirected graph B is a partition subgraph if it is a spanning subgraph and there is no connected component of the underlying undirected graph of P containing both v and v^c for any $v \in V$. Such a graph induces a non-unique partition of V into two components, labeled V^+ and V^-, with no directed edges between them, such that if $v \in V^+$, then $v^c \in V^-$.

A partition subgraph P of a bidirected graph B is called maximally connected if it has the fewest connected components out of all partition subgraphs.

The *undirected underlying graph* of a bidirected graph $B(V,E,C)$, denoted by $U(B)$, is defined as the undirected multigraph U with vertex set $V(U) = V/C$ (i.e., $\{u,u^c\}$ becomes a single node), and edge set $(u,v) \in E(U)$ if either u or u^c is adjacent to either v or v^c in B.

Proposition 7. For any bidirected graph B, a spanning forest of $U(B)$ that contains the minimum number of trees induces a maximally connected partition subgraph of B.

Proof. Let F be a spanning forest of $U(B)$ with the minimum number of trees. Define $\mathcal{F}$ to be the subgraph of B whose edges correspond to those in F (i.e., $\mathcal{F}$ is the restriction of B to complementary biedges which correspond to the edge set of F). Then $\mathcal{F}$ is a spanning subgraph of B whose connected components correspond to the trees of F.

Suppose a connected component $\mathcal{C}$ of $\mathcal{F}$ contains an edge v with directed paths to (or from) both u and u^c. Let $\mathcal{P}_1$ and $\mathcal{P}_2$ be these directed paths. By the definition of the underlying undirected graph, $C = U(\mathcal{C})$ is connected, and $U(\mathcal{P}_1) \cup U(\mathcal{P}_2)$ forms a cycle in C, which is a contradiction since $\mathcal{C}$ should not contain cycles. Therefore, $\mathcal{F}$ must be a partition subgraph of B.

Now, suppose there exists another partition subgraph of B, denoted $\mathcal{F}'$, with fewer connected components than $\mathcal{F}$. Then, $U(\mathcal{F}')$ would have fewer connected components than the number of trees in F, contradicting the assumption that F is a spanning forest of $U(B)$ with the minimum number of trees. Therefore, $\mathcal{F}$ is a maximally connected partition of B.

Using Proposition 7, we perform a DFS traversal and partition V into V^+ and V^- such that the number of inversion edges is minimized, meaning that during DFS traversal, any switch in orientation increases the number of inversion edges.

Cycle spaces and variant edges

A choice of reference tree for a pangenome graph is akin to a choice of basis for a vector space, specifically the cycle space of the underlying undirected graph. To fully understand this, it is necessary to introduce Eulerian subgraphs, which are subgraphs where each vertex has an even degree. The cycle space of a graph is a vector space formed by cycles over the field of two elements, using symmetric difference as the operation. The symmetric difference of two spanning subgraphs is the subgraph with edges that appear in exactly one of the original subgraphs. The fundamental basis of these cycle spaces consists of cycles formed by adding edges to a spanning tree, providing a minimal set of cycles that can generate any Eulerian subgraph through symmetric differences.

Considering a bidirected graph B, the underlying undirected representation, denoted $U(B)$, is a multigraph where each node corresponds to a binode in B, and nodes u and v are adjacent if $\{u,u^c\}$ and $\{v,v^c\}$ are adjacent in B.

From this foundational setup, the following sequence of propositions proves that any genetic variant (i.e., symmetric differences of given walks) can be captured using variant edges:

Proposition 8. The reference tree T of a bidirected graph B induces a spanning tree of $U(B)$, denoted by $U(T)$. Any variant edge in B induces an edge in $U(B)$ that is not an edge of $U(T)$.

Proof. By the definition of the underlying undirected graph, $U(T)$ is connected. Suppose $U(T)$ contains a cycle called C. Since T has no edge from any node u to u^c, C is not a loop (cycle with one node). Therefore, we may assume that C contains at least two nodes called u,v and hence there are two internally disjoint paths, between u and v. By the definition of the underlying undirected graph these two internally disjoint paths induce two paths from (u,u^c) to (v,v^c) in T that contradicts the definition of the reference tree (see "reference tree and variant edges").

Proposition 9. The symmetric difference of any two walks in B that start and end at terminus nodes induces an Eulerian subgraph in $U(B)$.

Proof. The degrees on the internal nodes are even in the symmetric difference since for each entry of a walk to a node it must exit once.

For the start terminus nodes each walk exits once and for the end terminus nodes each walk enters once. Therefore, in the symmetric difference the degree of the terminus nodes are either 0 or 2, depending on whether the two walks follow the same edge to exit or enter the terminus node or not.

Proposition 10. Every variant edge in B induces a cycle in $U(B)$. The set of these cycles forms the fundamental basis of the cycle space of $U(B)$.

Proof. Suppose T is a reference tree of B. Using Proposition 8, $U(T)$ is a spanning tree of $U(B)$ and any variant edge is not an edge of $U(T)$. Therefore, as established in Theorem 6.3.1 of West's textbook,[21] the fundamental cycles obtained by adding edges to a spanning tree form a basis for the cycle space of a connected graph.

Reference and alternative alleles

Recall that the half-trees of a bidirected tree $T=(V,E,C)$ are the directed subgraphs $T^+=(V^+,E^+)$ and $T^-=(V^-,E^-)$. T^+ is rooted. Let $e=(u,v)$ be a non-tree edge; in particular, it is allowed to be an inversion.

Given a variant edge (u,v) in the bidirected pangenome graph B and reference tree T, the *branchpoint* is defined as the lowest common ancestor of u^+ and v^+ in the half-tree T^+. The complement of the branchpoint is highest common descendant of u^-,v^- in T^-. Intuitively, the branchpoint marks the divergence between the reference path and the alternative path corresponding to the variant. It is the deepest node in T from which both u and v are reachable via directed paths. The branchpoint is used to compute reference and alternative alleles and is central to variant annotation, repeat detection (see "repeats") and graph simplification (see "simplified graph").

Let T' be the half-tree containing u, and let $v' = v$ or v^c, whichever one is contained in T'. Let b be the lowest common ancestor or highest common descendant of u,v' in T' (i.e., the branchpoint or its complement). Add (u,v) to T'. If $v \neq v'$, also add v, and also add the undirected edge (v',v).

In this graph, there exists one pair of parallel paths: one of the endpoints is b, and the other is either u or v. Call the latter endpoint a. One of those two paths contains the member of u,v which is not a; this is the "alternative path", and the other is the "reference path". Figure S15 shows reference and alternative paths for different scenarios.

The allele corresponding to a directed path $u_1, \ldots, u_n$ is the concatenation of the sequences of the nodes, except that when the path visits u and u^c consecutively, these sequences are skipped. Visually, this corresponds to a path that enters and exits from the same 'side' of a binode (Figure S16).

Proposition 11. For a variant edge $e=(u,v)$:

- *If u and v are both positive or negative*, *then* $\mathrm{Ref}(e^c)=(\mathrm{Ref}(e))^c$ *and* $\mathrm{Alt}(e^c)=(\mathrm{Alt}(e))^c$.
- *If u and v have different orientations*, *then* $\mathrm{Ref}(e) = \mathrm{Alt}(e^c)$ *and* $\mathrm{Alt}(e) = \mathrm{Ref}(e^c)$.

Proof. Let $e=(u,v)$ be a variant edge and let $e^c=(v^c,u^c)$ denote its complement. Let b denote the branchpoint of e, and let the reference and alternative paths be defined as in the caption of Figure S15.

Case 1: *u* **and** *v* **have the same orientation.** Then e and e^c lie entirely within the same half-tree (either T^+ or T^-), and the reference and alternative paths of e^c are exactly the reverse complements of those of e. Therefore, $\mathrm{Ref}(e^c)=(\mathrm{Ref}(e))^c$ and $\mathrm{Alt}(e^c)=(\mathrm{Alt}(e))^c$.

Case 2: *u* **and** *v* **have different orientations.** In this case, *e* is an inversion edge, and its complement e^c traverses the same binodes in reverse order but with reversed strand orientation. From Figure S15, we observe that the alternative path of *e* corresponds to the reference path of e^c, and vice versa, since switching the direction reverses the role of the tree-consistent and variant-containing paths. Therefore, Ref(e) = Alt(e^c) and Alt(e) = Ref(e^c).

Efficient computation of reference and alternative alleles

To efficiently compute reference and alternative alleles, we need to efficiently compute branch points for all variant edges. We use *Tarjan's offline lowest common ancestor algorithm*.[22] This classic algorithm performs a single linear-time traversal of the tree and answers each LCA query in nearly constant time, making it well-suited for large pangenome graphs with many variants.

Variant types

Variant edges in the pangenome graph are classified into four reference-dependent categories based on their structural characteristics relative to the reference tree. These categories are illustrated in Figure S13D:

- **Forward Edges:** These correspond to deletions, where the walk skips over a segment that is present in the reference tree. Such edges imply that a segment found in the reference genome is absent in the alternate genome.
- **Backward Edges:** These represent duplications, where the walk traverses a segment of the genome multiple times. This implies that a section of the genome is duplicated in the alternate path compared to the reference.
- **Crossing Edges:** These signify replacements, where one segment of a walk is replaced by another segment. Single Nucleotide Polymorphisms (SNPs) and Multiple Nucleotide Polymorphisms (MNPs) are replacements whose reference and alternative alleles are both one basepair long and both *n* basepairs long, respectively.
- **Inversions:** These edges connect nodes with opposite orientations (positive and negative directions) and result in a segment of the genome being reversed in orientation relative to the reference sequence. An inversion event corresponds to a *pair* of such inversion edges: the first edge switches the walk from forward to reverse orientation, and the second brings it back to the forward direction (or vice versa). Only when these inversion edges are properly paired can the walk rejoin the reference strand at the correct orientation; otherwise, the walk remains stranded in the reverse direction and cannot complete a valid traversal from one end of the genome to the other.

Repeats

The pangenome graph utilized in our study is based on the Minigraph-Cactus framework. Due to the construction methodology of minigraph, the resulting graph structure is almost a Directed Acyclic Graph (DAG), with very few back edges with respect to the DFS reference tree. This characteristic significantly reduces the occurrence of repeat variants within the graph. However, the node sequences themselves often contain repeated motifs. To address this, we developed and implemented an algorithm specifically designed to identify such repeat sequences within the nodes of the pangenome graph.

Algorithm 12. The algorithm identifies repeat motifs for a given variant edge:

1. Ensure the variant edge is not an inversion.
2. **Select Allele:** Choose the reference or alternate allele, depending on which is non-empty.
3. **Detect Motif:** For each divisor length *r* of the allele's length, check if the allele can be written as a perfect repeat of a substring of length *r*.
4. **Verify in Graph Context:** Check for the motif upstream of the branchpoint or downstream of the variant target node. If found, annotate it.

Missingness

In practice, haplotype assemblies are not fully contiguous; they contain gaps, due to the difficulty of assembling repetitive regions, and are represented as collections of walks. Between the end of one walk and the beginning of the next, there may exist many possible paths through the graph. We define a variant edge as being missing for a haplotype if that edge could be visited in between the end of one walk and the beginning of the next one. Directly identifying missing edges is computationally challenging, requiring a graph traversal for every walk. However, variant positions can be used to identify most missing edges.

Edges that violate the topological order inherited from the reference tree, when restricted to positive nodes, are called back edges. Removing the back edges from the graph restricted to positive nodes results in a directed acyclic graph (DAG). The right position of a positive node u^+ is defined as the smallest position of any node on the linear reference that is reachable from *u* in the DAG. Similarly, the right position of a binode (u^+,u^-) is defined as the right position of u^+ and denoted by rpos(u).

For a walk $W = \left(\left(u_1^+, u_1^-\right), \ldots, \left(u_k^+, u_k^-\right)\right)$, the linear coverage of *W* is the interval [*a*,*b*] where $a := min_{u \in W}\{\text{pos}(u)\}$ and $b := max_{u \in W}\{\text{rpos}(u)\}$. We identify as missing those variants of a haplotype to be the variant edges whose positions do not intersect the union of linear coverages of walks belonging to that haplotype. This approach identifies as missing all variant edges which can be traversed by a path that begins at the endpoint of one walk, terminates at the start point of the next walk, and visits some node on the linear reference (which could be one of those endpoints). However, it should be noted that this method may fail to detect a missing variant edge (u,v) that could be visited between the end of one walk, W_1, and the beginning of the next walk,

W_2, if the right position of v is greater than $\min_{u \in W_2}\{pos(u)\}$. This situation arises if the beginning of W_2 is reachable from v via a back edge (a,b) with $pos(a) > \min_{u \in W_2}\{pos(u)\}$ and $pos(b) \leq \min_{u \in W_2}\{pos(u)\}$.

Algorithm identifies missing variants for each haplotype.

Algorithm 13. The algorithm identifies missing variants for each haplotype using the positions and coverage of its walks:

1. **Compute Walk Coverage:** Compute the linear coverage interval $[a_W, b_W]$ where: $a_W := \min_{u \in W}\{pos(u)\}, b_W := \max_{u \in W}\{rpos(u)\}$
2. **Aggregate Haplotype Coverage:** Sum edge counts across walks and merge intervals to get total coverage for the haplotype.
3. **Detect Missing Edges:** Mark any unvisited variant edge as missing if its position does not intersect the haplotype's coverage.

Simplified graph

In complex regions of the pangenome graph, such as bubbles with multiple variant edges, numerous small variants often lie within larger variant structures, such as long indels with several SNPs on them. To simplify the analysis and visualization of these complex regions, we designed and implemented an algorithm that reduces the graph to a minor that retains only the long variants. This simplification helps in distilling the essential topological features of complex genomic regions.

Algorithm 14. The algorithm simplifies the subgraph of a complex genomic region by removing small variants:

1. **Filter by Allele Length:** Remove variant edges whose total allele length is below a given threshold.
2. **Identify Endpoints:** If endpoints are not provided, identify all nodes on the reference path with either in-degree or out-degree equal to zero.
3. **Delete Tips:** Iteratively remove tip-like structures that do not lie on paths between identified endpoints.
4. **Contract Simple Paths:** Merge linear paths—that is, paths in which all internal nodes have both in-degree and out-degree equal to one—between endpoints.

Handling non-path linear references

In some pangenome graphs, such as those produced by PGGB, the linear reference genome (e.g., GRCh38 or CHM13) is not embedded as a simple path. Rather, it may be represented as a walk that visits certain nodes or edges multiple times. The linear reference has a special significance in our approach because we use it to assign positions; however, we can still construct a valid reference tree and assign positions to all nodes and variant edges in a principled way.

Reference tree construction

Let L denote the linear reference walk in the bidirected graph B. Suppose the binodes of B have already been oriented as described in "orienting nodes within each binode".

Since we assume the bidirected graph has already been oriented, if the reference walk L contains any inversion edges (edges between nodes of opposite orientation), we discard those edges from L before constructing the reference tree. The remaining edges of L are then used to construct a spanning DFS tree $T_L = T_L^+ \cup T_L^-$. This tree includes all nodes visited in the forward and reverse orientations along the walk and consists of two disjoint half-trees: one rooted DFS tree on $V(L)^+$ (denoted T_L^+) and one directed tree on $V(L)^-$ (denoted T_L^-).

Next, we extend this DFS tree into a full spanning tree $T = T^+ \cup T^-$ of the entire graph B using a depth-first traversal starting from T_L^+. This process generalizes our construction for path-based linear references, where the linear reference path formed the first branch of the traversal. Here, the traversal begins from a rooted DFS tree, T_L. The resulting spanning tree T remains a valid reference tree for B, and all results established for path-based references, such as Theorem 1 (that genotypes determine walks up to cycles), and propositions 8–10 continue to hold.

Position assignment

When the linear reference L is not a path but a walk, certain nodes may be visited multiple times. This introduces ambiguity in defining a unique position for such nodes. To address this, we define positions as follows.

Let $u \in V$ be a node not on the linear reference walk L. Define pos(u) to be the position of its lowest ancestor in T_L. If u lies on L and is visited multiple times, we associate it with a set of positions (one for each occurrence in the walk) then assign *pos*(u) to be the set of all such positions.

For a variant edge $e=(u,v)$, we define its position *pos*(e) to be the smallest interval that contains all positions of u and v.

This position definition retains key theoretical guarantees. In particular, an analog of Theorem 6 holds: the genotype count $x_e(w)$ for any variant edge e in a walk w is determined by the counts of those variant edges whose positions overlap pos(e). Similarly, variant edge positions remain consistent under complementation, and variant reconstruction remains valid.

We still prefer to define positions with respect to a linear reference path. When the reference walk contains long cycles, it causes faraway positions to be superimposed, effectively making it useless to define any genomic region smaller than the length of the cycle. This is particularly suboptimal for recent SNPs that occurred after the emergence of a duplication event: such SNPs naturally belong to one copy or the other, not equally to both of them.

Comparing variants across graphs

Suppose two pangenome graphs are built from the same haplotypes. To check whether a variant in the first graph is also present in the second graph, one may use the following procedure:

1. pick a variant in the first graph;
2. choose a haplotype carrying its reference allele and record the position of that allele on that haplotype;
3. locate the same haplotype and position in the second graph;
4. check whether, in the second graph, that haplotype carries the reference allele at a variant edge with the same alternative allele.

This procedure detects cases in which the same variant is present in both graphs with the same representation. Its main limitation is that it will miss cases in which the same biological difference is represented differently in the two graphs, for example because of different node boundaries, different branch points, or a different choice of reference tree.

pantree VCF specification

Our variant call format (VCF) extends the standard specification, maintaining compatibility with conventional tools while also including pangenome-specific information.

Variant identifiers and canonical fields

Each variant is identified directly by its corresponding edge in the pangenome graph, replacing the traditional bubble ID convention. The CHROM, POS, REF, and ALT are preserved, but with critical reinterpretations:

- CHROM: The chromosome number.
- POS: The VCF position, which is a single integer, differs from our variant-edge position, which is a pair of integers (see 'PU' and 'PV' below). For indels such that we prepend a letter to both alleles, the VCF position of the variant edge (u,v) is the position of node u. Otherwise, it is one plus the position of u. The VCF is sorted based on POS.
- ID: The variant edge ID is a string concatenating the edge's node IDs of the pangenome graph from the original GFA file. Each node ID contains either '>' or '<' symbols that indicate the orientation of the node in the Pangenome graph ('>' means positive orientation and '<' means negative orientation). It should be noted that these orientations are inherited from the pangenome's GFA file, therefore, they might be different from the orientation of the reference tree as we orient the nodes (see STAR Methods, "orienting nodes within each binode").
- REF/ALT: Each variant edge corresponds to a pair of alleles: the reference allele derived from the reference tree, and the alternate allele from the non-reference path. The alternate allele is reported in the ALT field. If the reference allele lies entirely on the GRCh38 linear reference path, it is recorded in the standard REF field in the VCF. If the reference path does not align with the GRCh38 reference, the REF field is set to '.' because REF is required to match the linear reference genome. Non-GRCh38 reference alleles are recorded in the NR (non-reference reference allele) field (see below).

For indels on the linear reference, we follow the VCF convention that one letter - the letter preceding the indel on the linear reference - is prepended to both REF and ALT. We do not do so for non-GRCh38 indels, whose reference allele is recorded in the NR field instead of REF.

- QUAL: Set to a default value of 60.
- FILTER: Set to 'PASS'.
- INFO (**Customized VCF Fields).** The INFO field includes the following items:
  - **NR** (Non-Reference allele): The allele obtained by traversing the reference tree from the branchpoint to the end of the variant edge. If this path is off the GRCh38 linear reference, REF is set to '.' and NR holds the reference-tree-based allele (see STAR Methods, "reference and alternative alleles"). Otherwise, NR is '.'.
  - **VT** (Variant Type): Annotates the variant as one of the following: SNP, MNP, insertion, deletion, replacement, duplication, or inversion.
  - **DR** (Distance from Reference): A tuple capturing the distance of each node in the variant edge from the linear reference path (GRCh38).
  - **RC** (Reference allele Count): Total number of times all haplotype walks traverse the corresponding reference tree edge.
  - **AC** (Alternative allele Count): Total number of times all haplotype walks traverse the variant edge.
  - **AN:** Total number of times all haplotype walks traverse either the variant edge or the corresponding reference tree edge.
  - **PV** (Positions of variant nodes): A tuple storing the positions of nodes u and v in the variant edge (u,v).
  - **TR_MOTIF:** Contains the repeated motif if the variant edge corresponds to a local tandem repeat, otherwise '.'.
  - **NIA (Nearly Identical Alleles):** Nearly identical alleles are defined as allele pairs $\geq$10 bp differing by only one base (through substitution or a 1-bp indel), were flagged but retained. NIA is either 'yes' or 'no' flags nearly identical alleles.
- **Genotype data.** Each individual's phased genotype is represented (sample ID) using a structured format in the sample-level fields: GT: CR: CA.

CellPress
OPEN ACCESS

- **CR/CA**: Respectively count the number of times a haplotype visits the reference tree edge and the variant edge. The reference edge is defined as the last edge of the reference path. Because haplotype assemblies consist of multiple contigs represented as disconnected walks, some variant edges may not be visited simply due to gaps in assembly. In such cases, if the position interval of a variant edge falls entirely outside the haplotype's linear coverage, both CR and CA are marked as missing (.) in the VCF (see STAR Methods, "missingness").
- **GT** (Genotype): Indicates the presence or absence of the variant edge and the reference edge. GT is 1 if CA is positive, 0 if CA is 0 and CR is positive, and '.' if both are missing.
- **Missingness:** A variant is considered missing in a haplotype if all three fields (GT, CR, and CA) are '.'. This position-based strategy efficiently approximates true missingness without requiring exhaustive graph traversal (see STAR Methods, "missingness").

HPRC variant catalog

Chromosome-specific graph extraction

We extracted subgraphs for chromosomes 1–22, X, and Y from the full HPRC.gfa file (see resource availability). For each chromosome, we identified the node corresponding to its first occurrence in the graph and used gfatools to extract the chromosome-specific subgraph. This per-chromosome segmentation enabled parallelization and reduced memory requirements.

Non-GRCh38 variants

The variant edge (u,v) was classified as a non-GRCh38 variant if no GRCh38 walk visits v.

Variant length stratification

Variants were categorized as either large or small using their total allele length, defined as the sum of the reference and alternate allele lengths, and a threshold of at least 50 basepairs.

Repeat annotation

To annotate local tandem repeats and homopolymeric motifs, we identified indels such that (1) their non-empty allele is $(s)^n$ for some sequence motif s, and either (2a) the upstream sequence in the reference tree equals s, or (2b) some downstream sequence in the reference tree equals s. This approach recovers tandem repeat variants in cases where the *Minigraph-Cactus* graph topology lacks repeat structure (see STAR Methods, "repeats"). It can potentially miss copy number changes of $s \rightarrow (s)^2$ or $(s)^2 \rightarrow s$ when the matching motif is split between upstream and downstream sequences.

Region annotation

We used BED files from GIAB genomic stratifications.[19] Each variant was labeled as belonging to one of three categories: Easy, Segmental Duplication (Segdup), or Hard regions. Regions not explicitly labeled as Easy or Segdup were designated Hard. We annotated variants based on their VCF positions, excluding variants in segmental duplications from the hard category, such that the three categories formed a partition.

Intersection with CHM13 coding regions

To evaluate a practical use case of pantree's ability to call variation inside non-reference sequence, we intersected CHM13 haplotype positions of pantree variants with gene annotations on the T2T-CHM13 v2.0 assembly. We downloaded the NCBI RefSeq v110 annotation for CHM13 and extracted gene, exon, and CDS intervals for all autosomes and chromosomes X and Y, mapping NCBI accession identifiers to standard chromosome names before overlap analysis.

We used the VCF generated from the haplotype-contiguous DFS version of pantree on the Year 2 HPRC Minigraph-Cactus graph (233 samples), with GRCh38 and CHM13 as priority samples. Non-GRCh38 variants were defined as those with REF = '.'. We then restricted to the subset of non-GRCh38 variants with available CHM13 haplotype positions (HP) in this VCF and asked whether their HP values fell within annotated gene, exon, or CDS intervals on CHM13. The same procedure was applied to on-GRCh38 variants with available CHM13 haplotype positions for comparison. The genome-wide analysis was restricted to autosomes and chromosome X, because chromosome Y had no variants with CHM13 haplotype positions in the Year 2 graph. Genes with CDS-overlapping variants were counted after collapsing across transcripts to the gene level.

Insertion and deletion superbubbles

A superbubble is defined as an insertion if the first node and the last node of the superbubble are adjacent via a reference-tree edge. Similarly, a superbubble is defined as a deletion if the first node and the last node of the superbubble are adjacent via a variant edge.

Performance and resource usage

Our pipeline was parallelized by chromosome. On chromosome 1, pantree was run using 1 CPU core and 128 GB requested memory on an Intel Xeon Gold 5320 CPU (2.20 GHz; 257 GB RAM); it completed in 6 h 48 min and reached a peak memory usage of 34.48 GB. For comparison, vg deconstruct was run on chromosome 1 using 1 CPU core on an Intel Xeon Platinum 8468 CPU (1 TB RAM); it completed in 27 min and reached a peak memory usage of 13.74 GB. Across all chromosomes, pantree required 447 GB of peak memory and 55 h of wall time in total (see Table S18).

Validation and methods comparison

SNP benchmarking against HPRC VCFs

We compared SNPs identified in our output VCF with those in the original HPRC VCF and *vcfwave*. SNPs were matched based on position, reference allele, and alternative allele. Variants found in both datasets were considered shared, while those present in only

one were labeled as dataset-specific. This comparison was limited to SNPs because for non-SNPs, matching can be ambiguous; for example, a deletion in a homopolymer can be coded in several different ways, with different positions but equivalent sequence effects.

non-SNP benchmarking against the raw VCF

To benchmark *pantree*'s handling of non-SNP variants, we compared biallelic superbubbles from the HPRC raw chr20 VCF with variants in the *pantree* VCF. From the raw VCF, we selected variants where GRCh38 and CHM13 carry different alleles and removed sites with ambiguous bases in any allele. Using the superbubble annotation file, we classified each variant as biallelic (single ALT) or multiallelic (multiple ALTs) and labeled it as an insertion (there is a reference tree edge from beginning to end of the bubble), deletion (there is a variant tree edge from beginning to end of the bubble), or ''other'' event. For the non-SNP biallelic subset, we then asked whether the *pantree* VCF contained a variant with the same chromosome, position (POS), REF, and ALT, and counted such variants as matches.

Assembly-based validation

To evaluate small-variant accuracy against an external truth set, we used the small variant calls of HG002 from the GIAB (Genome in a Bottle) Q100 v1.1 dataset as ground truth.[18] We then benchmarked graph-based callsets against this truth set using *RTG vcfeval*, which reports SNP and INDEL errors by comparing each callset to the truth variants within the benchmark regions.

SNP benchmarking on a CHM13 contig

To benchmark a subset of non-GRCh38 variants against the *vg deconstruct* raw VCF, we compared *pantree* SNPs to *vg deconstruct* when a CHM13 contig was used as the reference path. On the Year 1 chr20 *Minigraph-Cactus* graph (constructed with GRCh38 as the guiding reference), we selected the CHM13#0#chr20 contig ($\sim$26.3 Mb, spanning $\sim$40% of the chromosome) and ran *vg deconstruct* with this contig as the reference path to obtain a raw SNP VCF on that path. As in the GRCh38-based comparison, this raw VCF includes SNPs arising from the full hierarchy of snarl sites, including nested snarls. We then restricted *pantree* SNPs to those where CHM13 has an ALT allele and matched them to SNPs from the *vg deconstruct* raw VCF by comparing the end node of each bubble with swapped REF/ALT alleles (since the raw VCF uses CHM13 as reference while *pantree* uses GRCh38).

Cross-graph comparison of variants

To assess whether pantree variants are reproducible across different pangenome graphs, we compared variant catalogs generated from the Year 1 (v1, 46 samples) and Year 2 (V2.1 233 samples) HPRC Minigraph-Cactus graphs for chromosome 21. Both VCFs were generated using pantree with the haplotype-contiguous DFS method and GRCh38 and CHM13 as priority samples, producing haplotype positions (HP) along both walks.

The comparison uses a two-coordinate key to identify variants across graphs: the GRCh38 position (POS), which is the position of the branchpoint's ancestor on the linear reference, and the CHM13 haplotype position (HP), which is the position of the branchpoint along CHM13's walk. Since CHM13 uses the same T2T assembly in both graphs, its HP provides a stable physical coordinate. However, the two GFA files encode CHM13 contig starts differently (Year 1 uses '*', parsed as 0; Year 2 uses actual genomic coordinates), introducing a contig-dependent offset. We computed this offset by matching on-GRCh38 SNPs between the two VCFs: for each SNP present in both, we recorded the difference in CHM13 HP. The dominant offset (11,310,098, shared by 91.7% of matched SNPs) identifies the largest CHM13 contig.

We restricted the cross-graph comparison to variants belonging to this dominant CHM13 contig. For on-GRCh38 variants, a Year 1 variant was assigned to the dominant contig if a Year 2 variant at the same GRCh38 POS had a CHM13 position matching the Year 1 value after offset correction. We then matched Year 1 variants to Year 2 by requiring both GRCh38 POS and CHM13 HP to match exactly, with no position window. For non-GRCh38 variants, we restricted to those with an available CHM13 haplotype position belonging to the dominant CHM13 contig. Because non-GRCh38 variants lack a standard linear-reference coordinate for the reference allele, the CHM13 haplotype position is particularly important for cross-graph identification of these variants.

Alleles were compared using three criteria of decreasing stringency: (1) exact allele matching, requiring the unordered pair of allele sequences {allele1, allele2} to be identical; (2) variant-type matching, requiring the same variant classification (SNP, DEL, INS, MNP, or REP) at the same position; and (3) subsequence matching, requiring one variant's allele to contain the other's as a substring, or vice versa, to account for cases where a graph with more samples decomposes a variant more finely.

Multiallelic superbubbles

Level 0 superbubbles/snarls

We used published .snarls files (see resource availability) to assign variant edges to level-0 sites. The .snarls file encodes a hierarchy of snarl sites with levels 0 to 28 and defines each site by listing its constituent nodes and an associated ID. In this manuscript, when analyzing multiallelic superbubbles, we use the term superbubble to refer to a level-0 snarl, since at level 0 these sites correspond to the same basic single-entry, single-exit structure in the bidirected graph that superbubbles represent in the directed setting.

Assignment of variant edges to superbubbles

A variant edge was assigned to a level-0 superbubble if both of its nodes were present within the same superbubble; we confirmed that every variant edge is assigned to exactly one superbubble. This approach was applied genome-wide across all 22 autosomes, as well as chromosomes X and Y. We found that every variant edge was contained within a superbubble, except those involving artificial terminus nodes used for reference tree construction.

Classification of triallelic structures

We defined a superbubble as triallelic if it contained two variant edges. Triallelic superbubbles were categorized into four types: properly triallelic, overlapping, nested, and interlocking. The classification was based on the two branch points of the variant edges within the bubble and the two end nodes of the bubble:

Properly triallelic: Bubbles where both start and end nodes have degree three.
Overlapping: Bubbles where exactly one of the start or end node has degree three.
Nested: Bubbles where one of the variant edges has the same branchpoint as the starting node of the bubble and end node as the last node of the superbubble.
Interlocking: Bubbles where one of the variant edges has the same branchpoint as the starting node of the bubble and the other variant edge has the same end node as the last node of the superbubble.

Complex regions

Region preprocessing and positioning

We extracted subgraphs corresponding to specified genomic intervals of interest. These intervals were selected based on their known structural complexity or biological relevance, and their positions are reported in Table S19.

The genomic coordinates for the HLA and RHD regions were obtained from the NCBI Genome database (hg38). Using these coordinates, we manually identified potential superbubbles corresponding to the target genes by locating the relevant node IDs in the generated VCF file. We then visualized these regions using *Bandage* and compared the resulting graph structures to those presented in the first draft of the human pangenome ref.[4] We used this comparison to refine the exact node IDs and positions associated with the HLA and RHD loci.

Simplification of complex variant structures

To enable effective interpretation of complex genomic regions, particularly those containing bubbles with dense clusters of small variants, we applied a three-step graph simplification procedure. First, we removed "small" variant edges whose combined reference and alternative allele length was <50 bp. Second, we pruned tip-like structures that were not required to maintain connectivity between the remaining nodes. Third, we contracted linear paths comprising only degree-two internal nodes that are traversed by all remaining haplotypes (obligatory edges). This pipeline discards only edges that cannot serve as reference or alternative alleles for any *pantree* variant and therefore does not change the set of variant edges in the region (see STAR Methods, "simplified graph"). The resulting graphs retain long, structurally informative variants, which we visualized as *Bandage* plots (see resource availability).

QUANTIFICATION AND STATISTICAL ANALYSIS

No null-hypothesis statistical tests were used unless explicitly stated. Analyses consisted of deterministic graph algorithms, variant counting, overlap analyses, and benchmarking against existing callsets or truth sets. Sample sizes are reported as exact counts of variants, variant edges, haplotypes, superbubbles, genes, or genomic intervals, depending on the analysis, and are specified in the corresponding Results text, figure legends, and supplemental tables. Percentages were calculated from the exact counts shown in the figures or supplemental tables. No randomization, blinding, or sample-size estimation was performed. Benchmarking against HG002 used RTG vcfeval as described in method details, with false-positive and false-negative counts reported separately for SNPs and indels.

ADDITIONAL RESOURCES

No additional resources are reported.

Cell Genomics, Volume *6*

Supplemental information

Defining and cataloging variants

in pangenome graphs

Pouria Salehi Nowbandegani, Shenghan Zhang, Haoyang Hu, Heng Li, and Luke J. O'Connor

Supplementary Figures

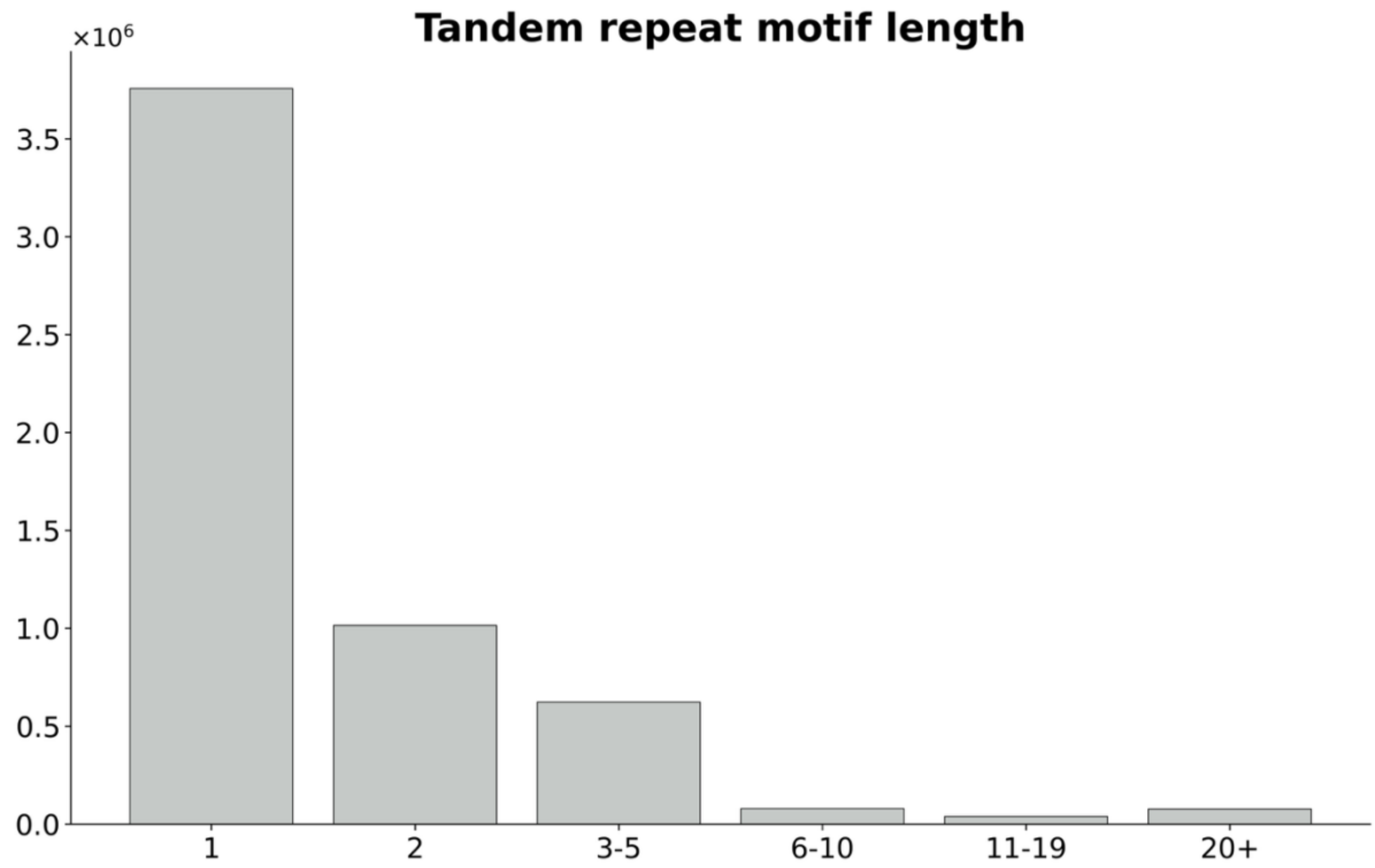

Figure S1. Distribution of tandem repeat motif lengths, related to Figure 2. Tandem repeat motif length was defined as the length of the 'TR_motif' field in the pantree VCF.

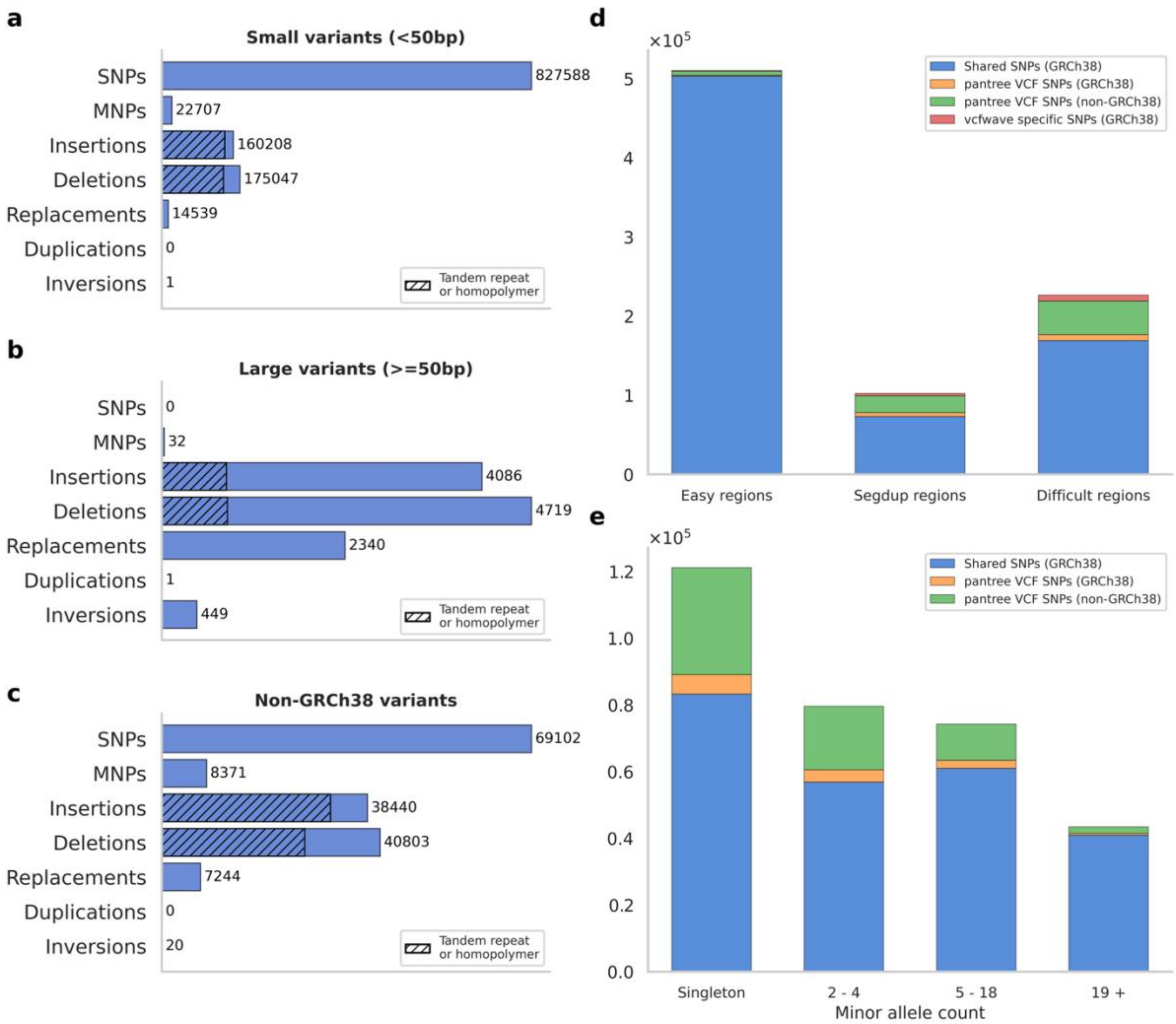

Figure S2. Variants on chromosome X, related to Figure 2 and Figure 3. (a) Number of small variants (<50 bp combined allele length) across seven non-overlapping variant types. Hatched area indicates the number of variants that cause a change in the copy number of a reference-tree basepair sequence. (b) Number of large variants. (c) Number of non-GRCh38 variants, defined as variant edges connecting two non-GRCh38 nodes. (d) Overlap of SNPs with those reported by *vcfwave*, across three non-overlapping genome annotations. (e) Minor allele count (the minimum of the reference allele count and alternative allele counts) out of 90, stratified by overlap with *vcfwave*.

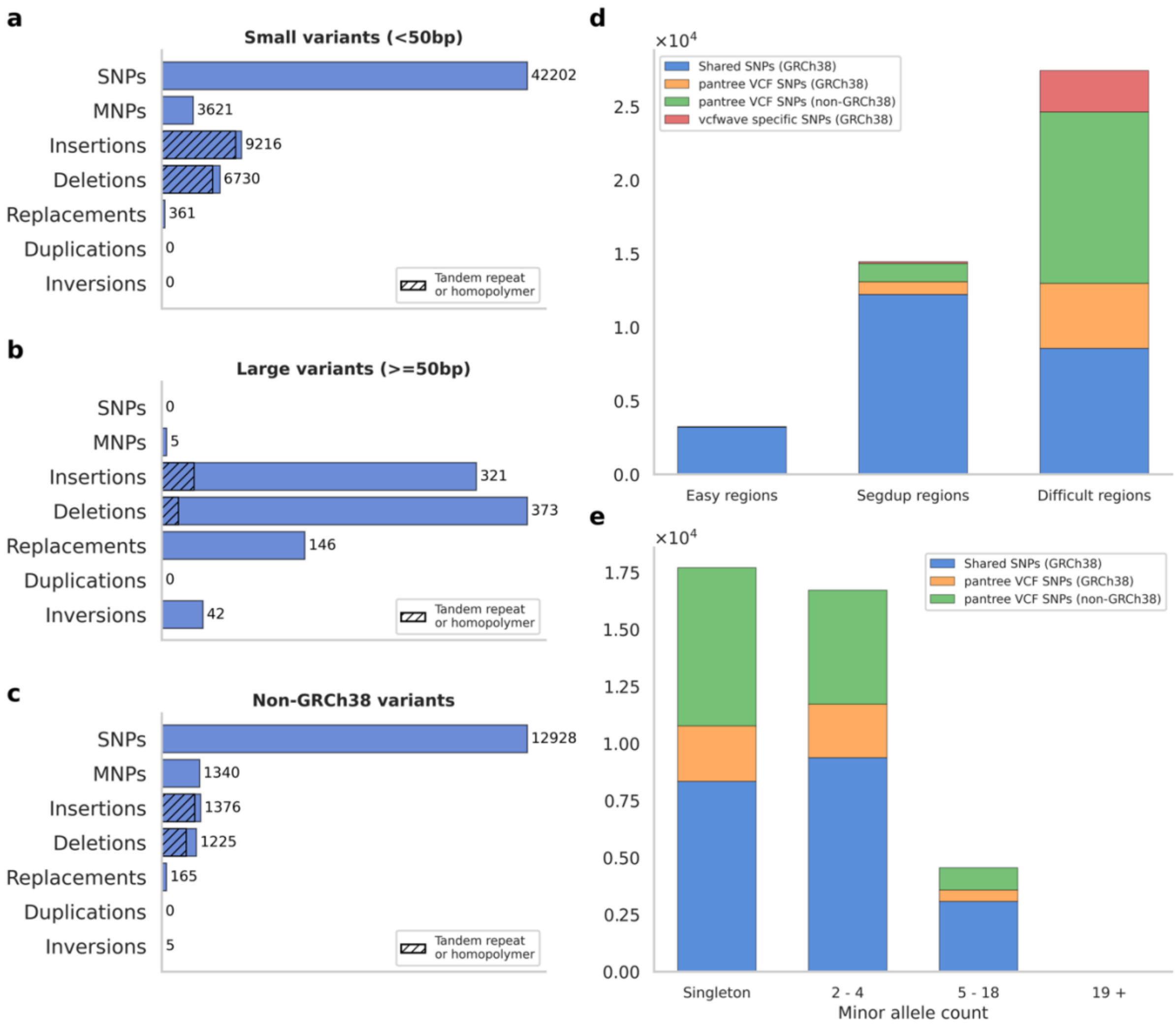

Figure S3. Variants on chromosome Y, related to Figure 2 and Figure 3. (a) Number of small variants (<50 bp combined allele length) across seven non-overlapping variant types. Hatched area indicates the number of variants that cause a change in the copy number of a reference-tree basepair sequence. (b) Number of large variants. (c) Number of non-GRCh38 variants, defined as variant edges connecting two non-GRCh38 nodes. (d) Overlap of SNPs with those reported by *vcfwave*, across three non-overlapping genome annotations. (e) Minor allele count (the minimum of the reference allele count and alternative allele counts) out of 21, stratified by overlap with *vcfwave*.

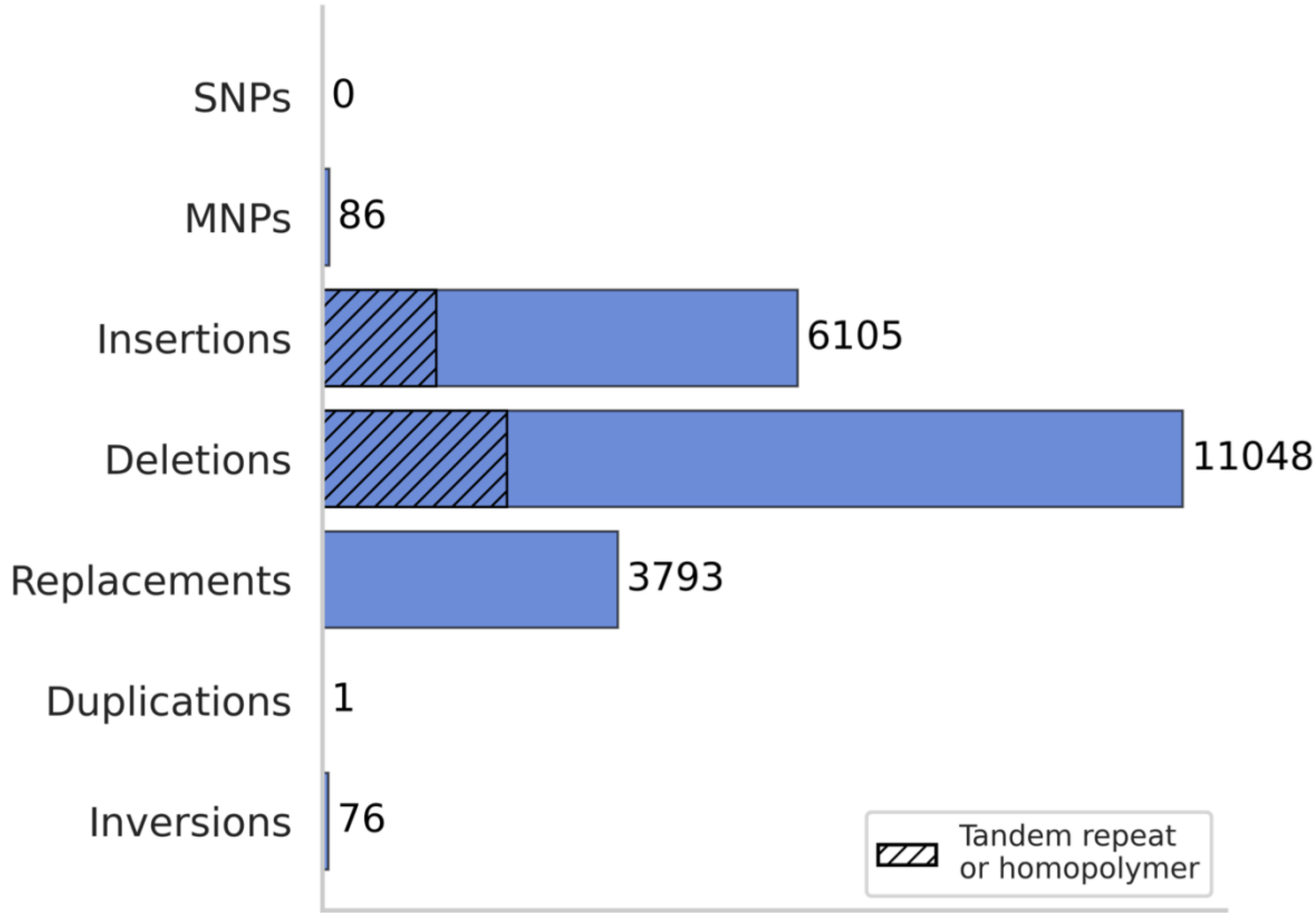

Figure S4. Large, common non-GRCh38 variants, related to Figure 2. Total counts per variant class (SNP, MNP, insertion, deletion, replacement, duplication, inversion) for non-GRCh38 variants with a minor allele count of at least 5 and a combined allele length of at least 50 bp. Hatched bars denote variants that cause a repeat expansion or contraction, including all duplications.

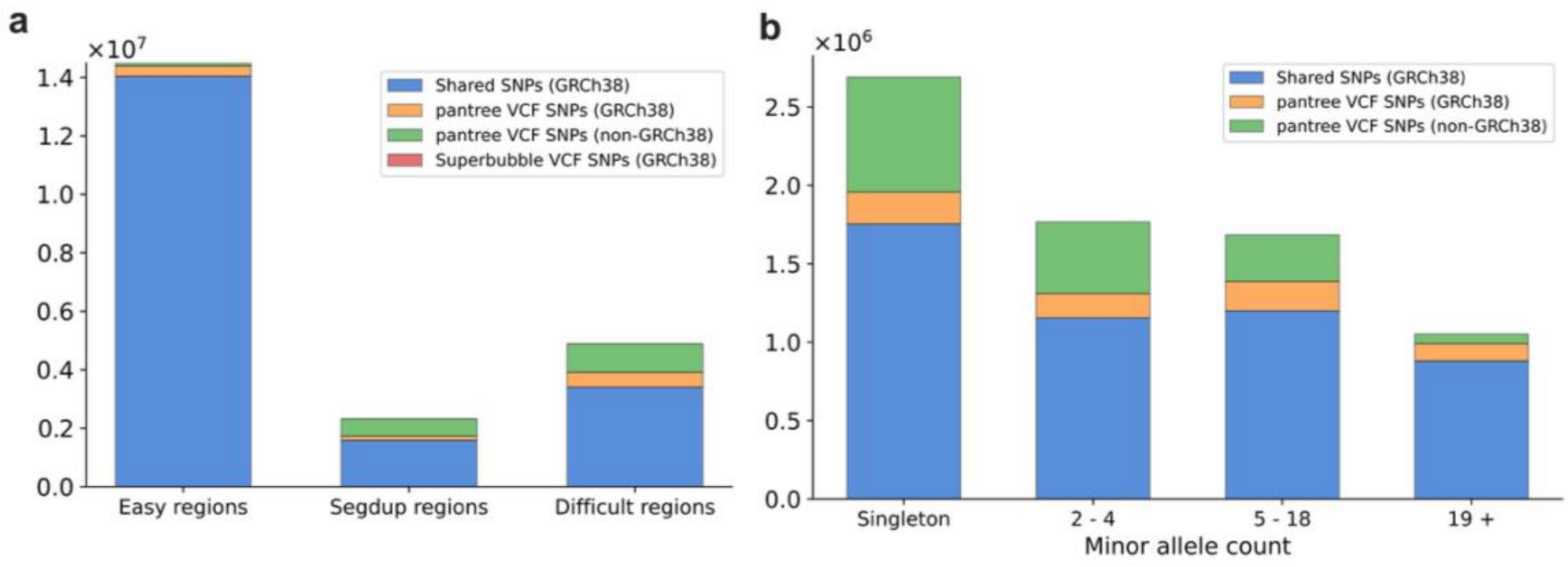

Figure S5. SNPs detected using pantree versus superbubbles, related to Figure 3. SNPs detected using pantree vs. superbubbles. (a) SNPs were partitioned by their VCF position into easy regions, segmental duplications, and other difficult regions. Bars indicate the number of SNPs detected using both methods, the number of GRCh38 and non-GRCh38 SNPs found by pantree only, and the number of SNPs found using superbubbles only (there were exactly 2 of these). (b) SNPs separated by their minor allele count, defined using pantree (the minimum value between reference allele counts and alternative allele count). Superbubble VCF specific SNPs were not included because they did not have allele counts reported.

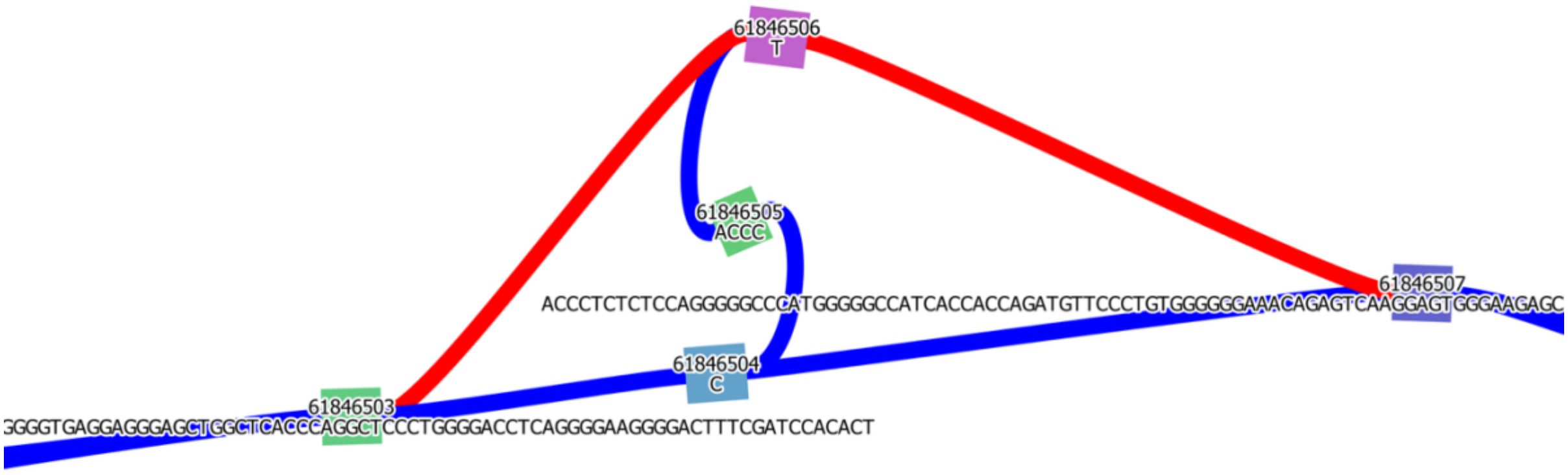

Figure S6. Superbubble-specific SNP on chromosome 6, related to Figure 3. Exactly two SNPs were detected as superbubbles without being called as SNPs by pantree. This superbubble, on chromosome 6, coincides with an assembly gap; there exists a terminal edge from the negative starting terminus to the node 61846506, which means that one walk starts in the middle of the superbubble from the node 61846506, then goes through the nodes 6184605 and 6184604, then to the pinch-point 6184603. Because superbubble alleles are required to pass through both ends of the superbubble, this walk, whose allele would be 'CACCCT', is not counted as an alternative allele, and the superbubble is classified as a SNP. pantree identifies an insertion ('ACCCT') and a deletion ('ACCC').

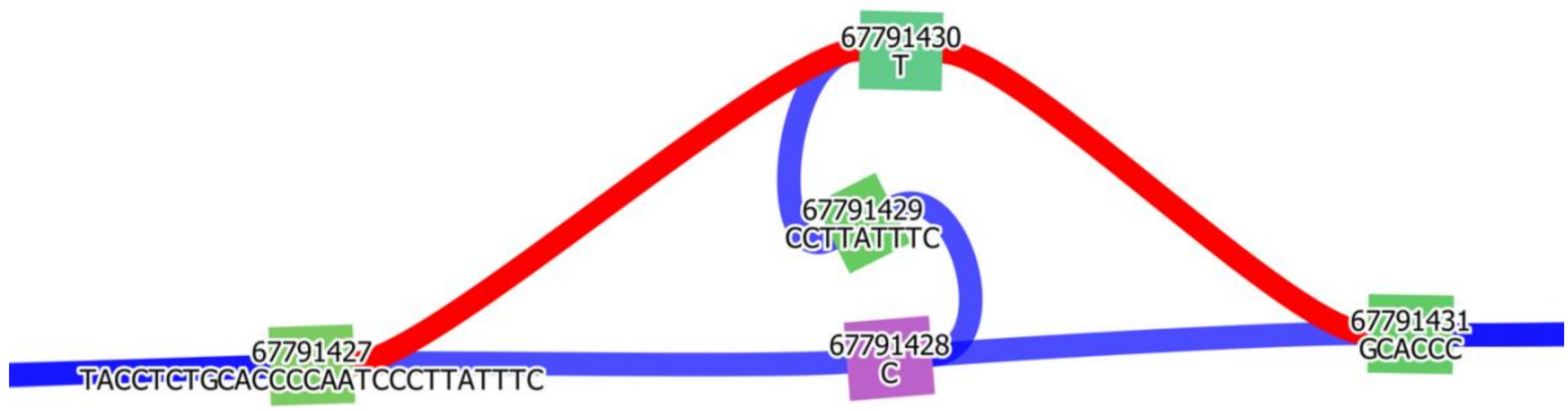

Figure S7. Superbubble-specific SNP on chromosome 7, related to Figure 3. Exactly two SNPs were detected as superbubbles without being called as SNPs by pantree. This superbubble, on chromosome 7, coincides with an assembly gap (similar to the SNP in Figure S6). There is a terminal edge from the node 67791428 to the negative ending terminus, and a walk which begins within node 67791431 following the negative orientation, then goes through the nodes 67791430 and 67791429, then ends at 67791428. Thus, the ’CCCTTATTTCT’ allele did not show up as an alternative allele in this superbubble.

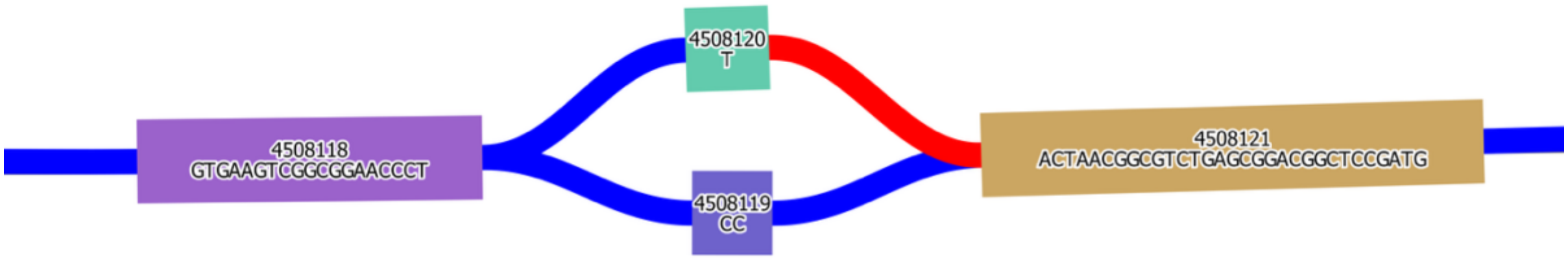

pantree VCF
```
CHROM POS       ID                  REF  ALT  INFO
chr1  248826452 <4508120>4508121    CC   T    NR=.
```
superbubble VCF
```
chr1  248826451 >4508118>4508121    TCC  TT
```
vcfwave
```
chr1  248826451 >4508118>4508121_1  TC   T
chr1  248826453 >4508118>4508121_2  C    T
```

Figure S8. vcfwave-specific SNP inconsistent with the graph, related to Figure 3. pantree identifies a replacement whose reference allele is CC and alternative allele is T, and the ‘raw’ superbubble VCF has a similar variant (except prepending the last letter of the branch point to both alleles). *vcfwave* produced a different alignment and identified two variants, including a C-to-T SNP which is inconsistent with the graph.

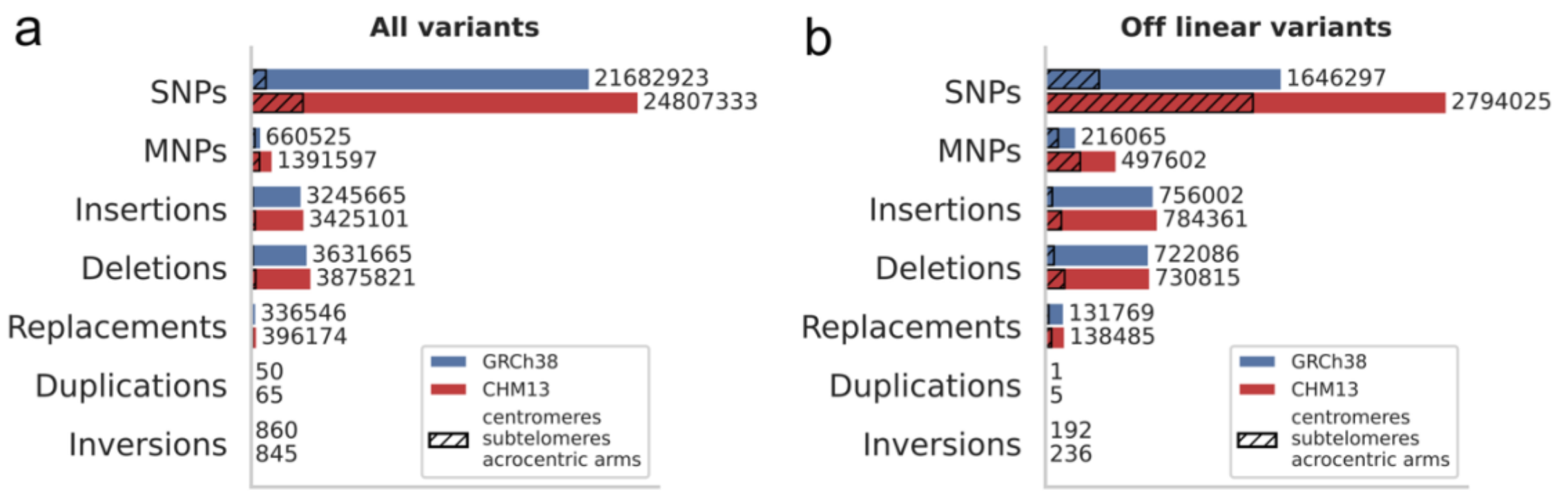

Figure S9. Variant comparison between the CHM13 and GRCh38 graphs, related to Figure 3. These graphs are based on the same data and the same algorithm, Minigraph-Cactus, but the algorithm is initialized at one or the other linear reference genome. The graphs differ in combined sequence length because regions missing from GRCh38 are excluded from the GRCh38 graph. These non-GRCh38 regions mostly include centromeres, subtelomeres, and acrocentric chromosome arms. (a) The number of variants detected by pantree in each graph. (b) The number of non-GRCh38 variants in the GRCh38 graph and the number of non-CHM13 variants in the CHM13 graph.

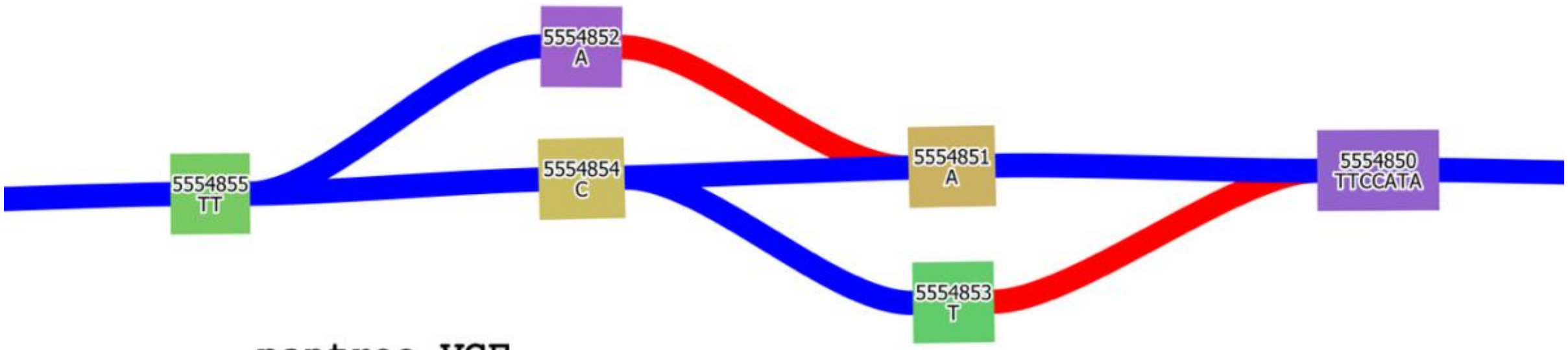

pantree VCF

```
CHROM POS       ID                  REF  ALT  INFO
chr1  125169613 >5554852>5554851    C    A    NR=.
chr1  125169614 >5554853>5554850    A    T    NR=.
```

superbubble VCF

```
chr1  125169613 >5554855>5554850    CA   CT
```

vcfwave

```
chr1  125169614 >5554855>5554850_1  A    T
```

Figure S10. SNP missed by vcfwave due to an assembly gap, related to Figure 4. pantree identifies two SNPs. One walk terminates at the node 5554851, and as a result, the ‘AA’ allele is not listed in the superbubble ‘raw’ VCF. As a result, the *vcfwave* VCF misses the ‘C’ to ‘A’ SNP.

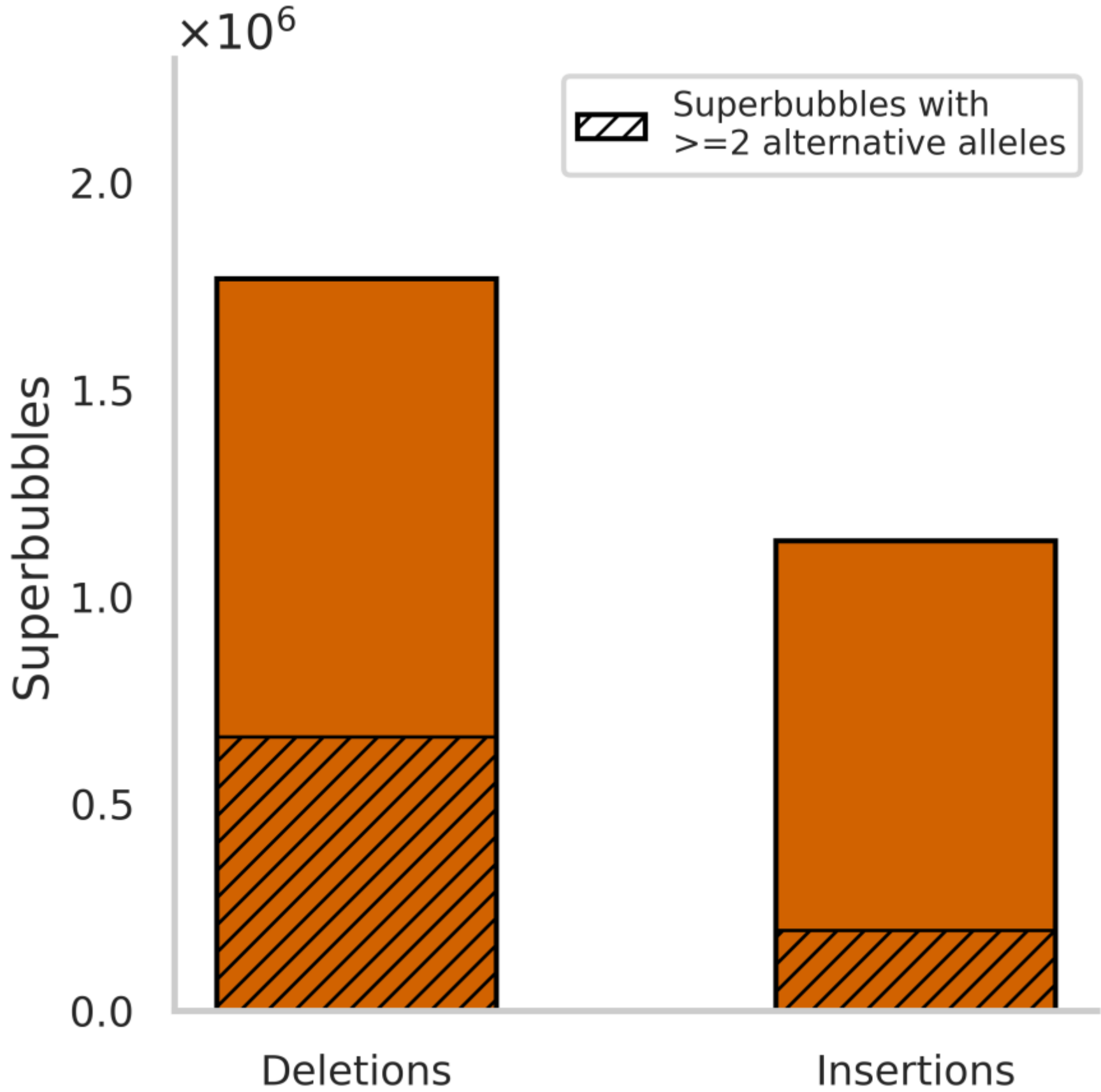

Figure S11. Proportion of insertion and deletion superbubbles, related to Figure 4. Among all superbubbles, there are 55.7% more deletion superbubbles (1,767,792) than insertion superbubbles (1,135,061). Among all multiallelic superbubbles, there are 241.6% more deletion superbubbles (661,196) than insertion superbubbles (193,553). Overall, 37.4% of deletion superbubbles are multiallelic, versus 17.1% of insertion superbubbles. This asymmetry results from the following ascertainment effect. Suppose that among all insertion or deletion superbubbles (biallelic or multiallelic), the average frequency of the empty deletion allele equals one half, such that GRCh38 has a 50% probability of having that allele. When restricting to multiallelic superbubbles, the average frequency should not equal one half, because a rare insertion is much less likely to be polymorphic compared with a common insertion (for example, a singleton insertion cannot be polymorphic). As a result, multiallelic superbubbles have a greater frequency of insertion alleles, and if GRCh38 carries a randomly sampled allele, then multiallelic indel superbubbles will usually be classified as deletions relative to GRCh38.

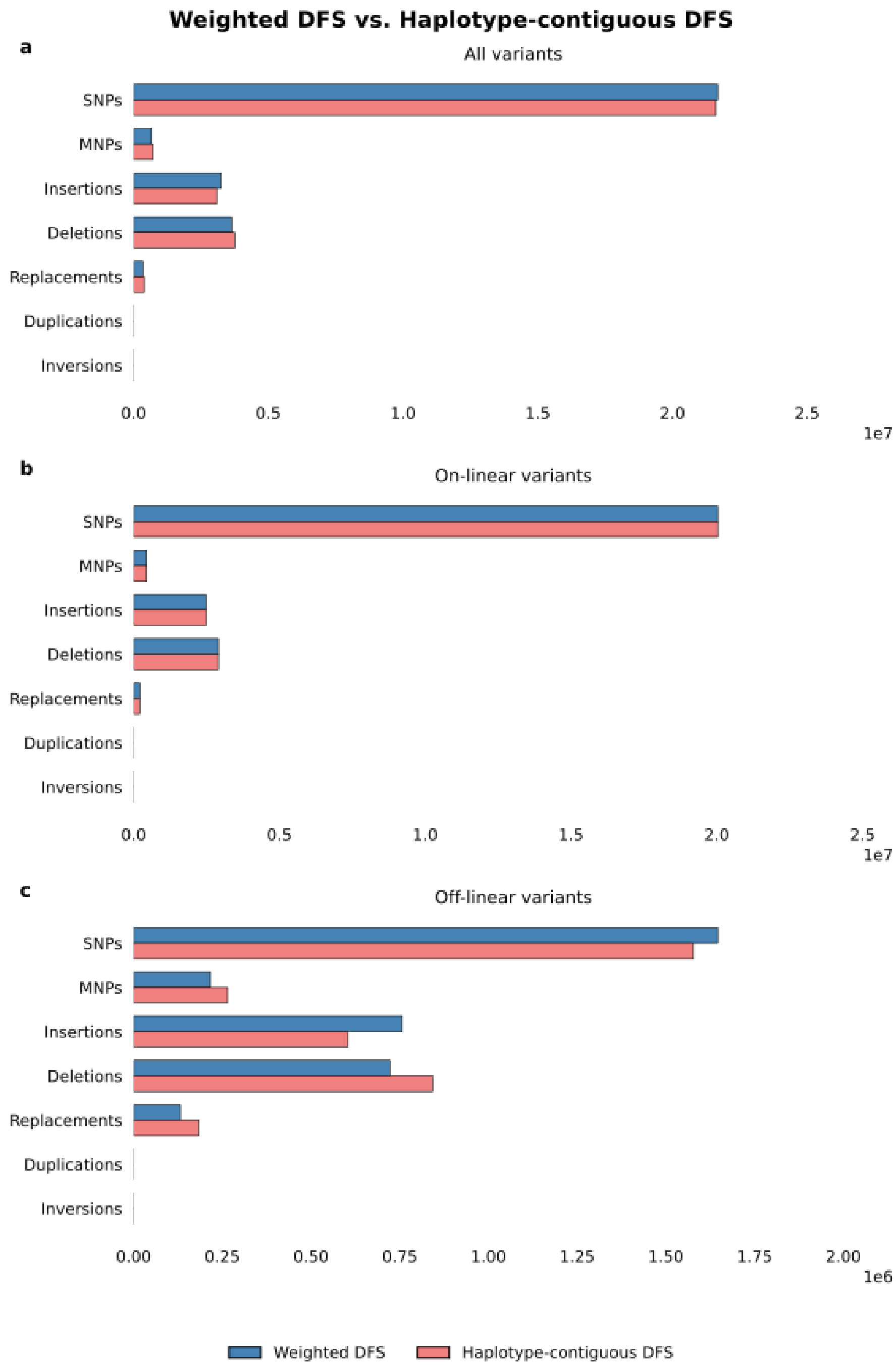

Figure S12. Comparison of variants defined by weighted DFS and haplotype-contiguous reference trees, related to Discussions. Variants were called on the same pangenome graph using two different reference-tree constructions: a weighted depth-first search (DFS), which prioritizes edges traversed by many haplotypes, and a haplotype-contiguous DFS, which prioritizes continuity along selected haplotypes. Panels a-c show counts for all variants, on-linear variants, and off-linear variants, respectively. Within each panel, bars for the two reference-tree choices are displayed side by side. Although individual variant edges differ between the two reference trees, the overall numbers of variants and the distribution of variant types are highly similar.

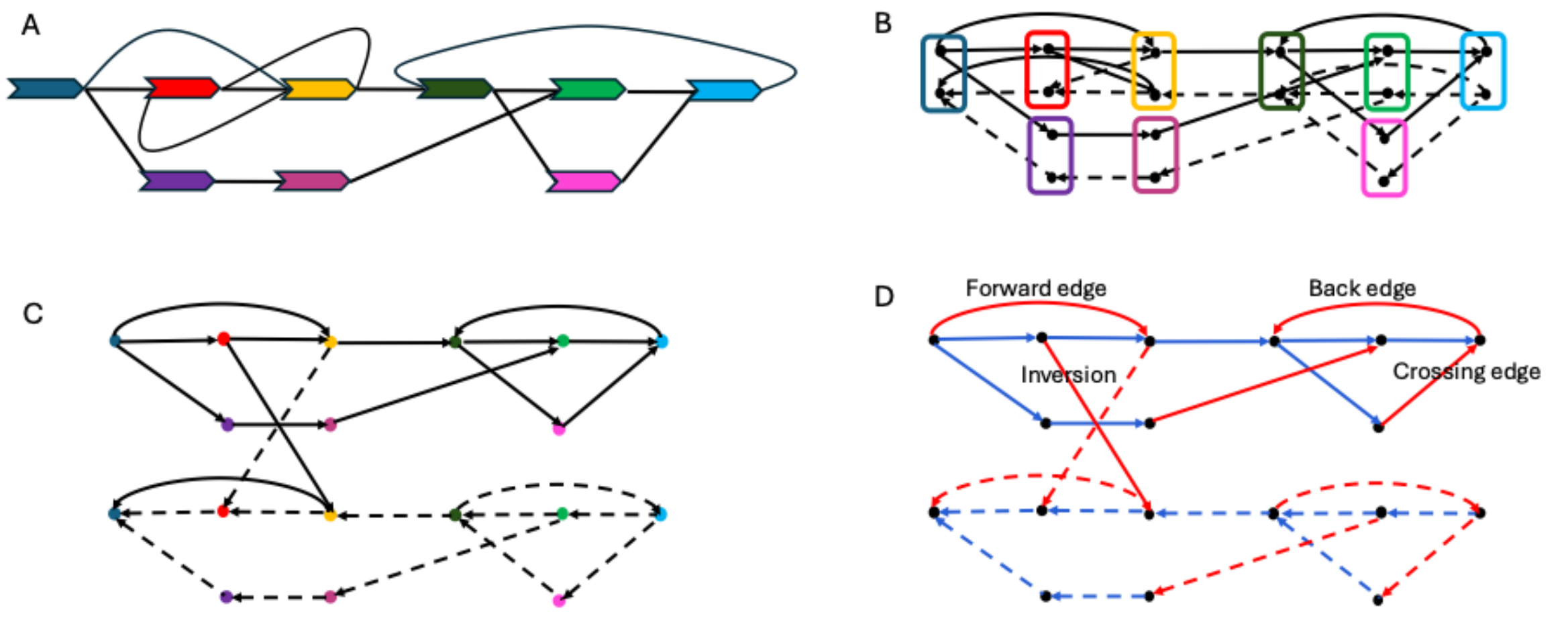

Figure S13. Bidirected and directed representations of a pangenome graph, related to STAR Methods. (A) Bidirected representation of a bidirected graph. Each binode is shown as a double-headed arrow. Biedges connect either heads or tails depending on traversal orientation. For example, the biedge between the purple and dark pink binodes connects the forward orientation of the purple binode to the forward orientation of the dark pink binode, and the reverse orientation of the dark pink binode to the reverse orientation of the purple binode. (B) Directed representation of the same graph, where each binode is unfolded into two complementary nodes, one representing forward orientation and one representing reverse orientation. The color scheme follows panel (A). Each biedge corresponds to a pair of directed edges—one shown solid, one dashed. (C) An expanded version of (B), where complementary nodes are spatially separated for clarity. (D) The reference tree highlighted in blue. The tree over forward oriented nodes forms a DFS tree. Variant edges (in red) are classified relative to this tree as forward, back, or crossing edges. Inversion edges connect a forward oriented node to a backward oriented node.

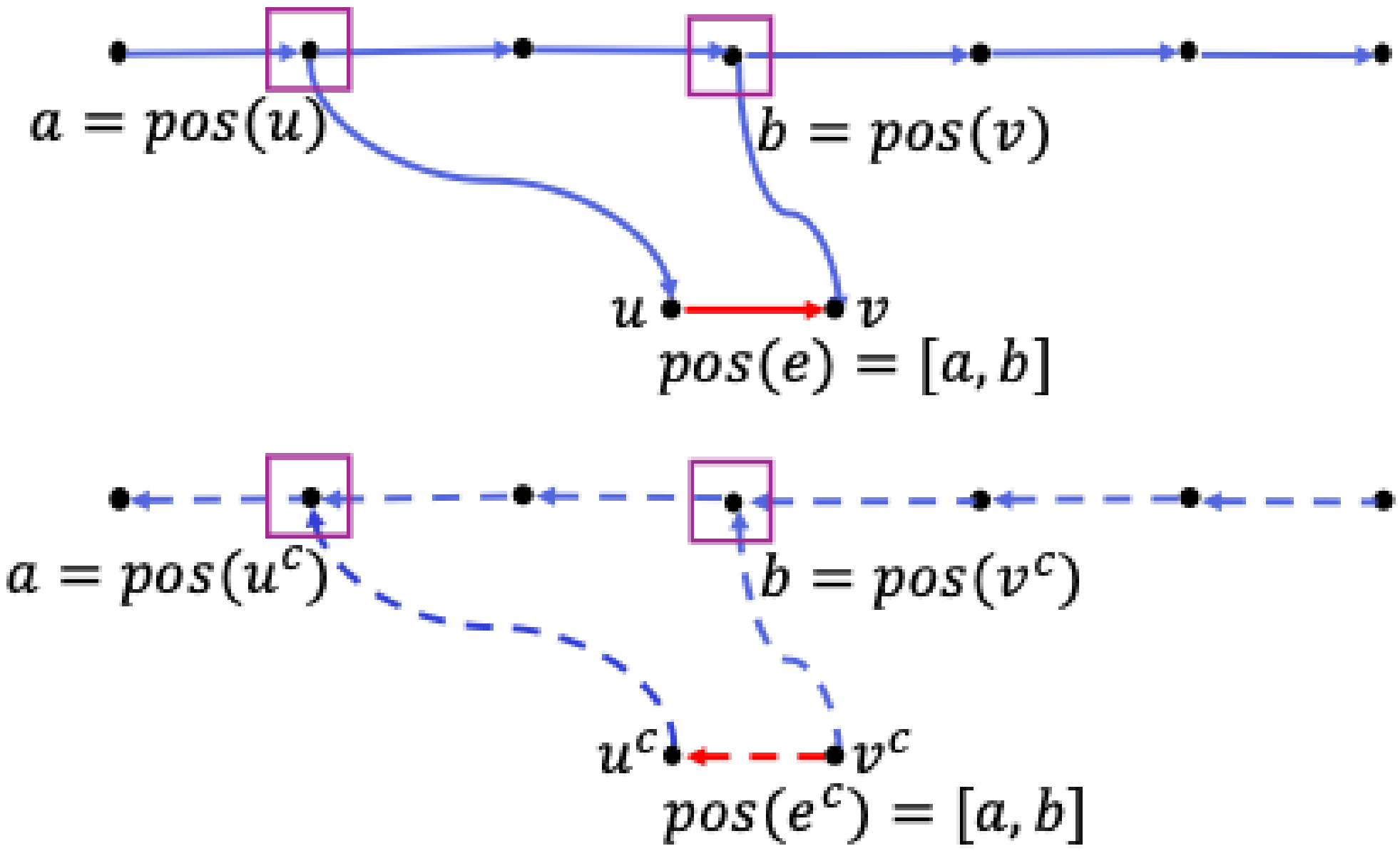

Figure S14. Position of a variant edge and its complement, related to STAR Methods. The blue paths represent the linear reference in forward (solid) and reverse (dashed) orientations. The position of a node v is defined as the position of its lowest ancestor in the forward-oriented linear reference (or its first descendant in the backward-oriented reference). For a variant edge $e = (u, v)$, the position is defined as the interval pos(e) = [pos(u),pos(v)]. The position of a node is the same as that of its complement, and similarly, the position of a variant edge is equal to the position of its complementary edge.

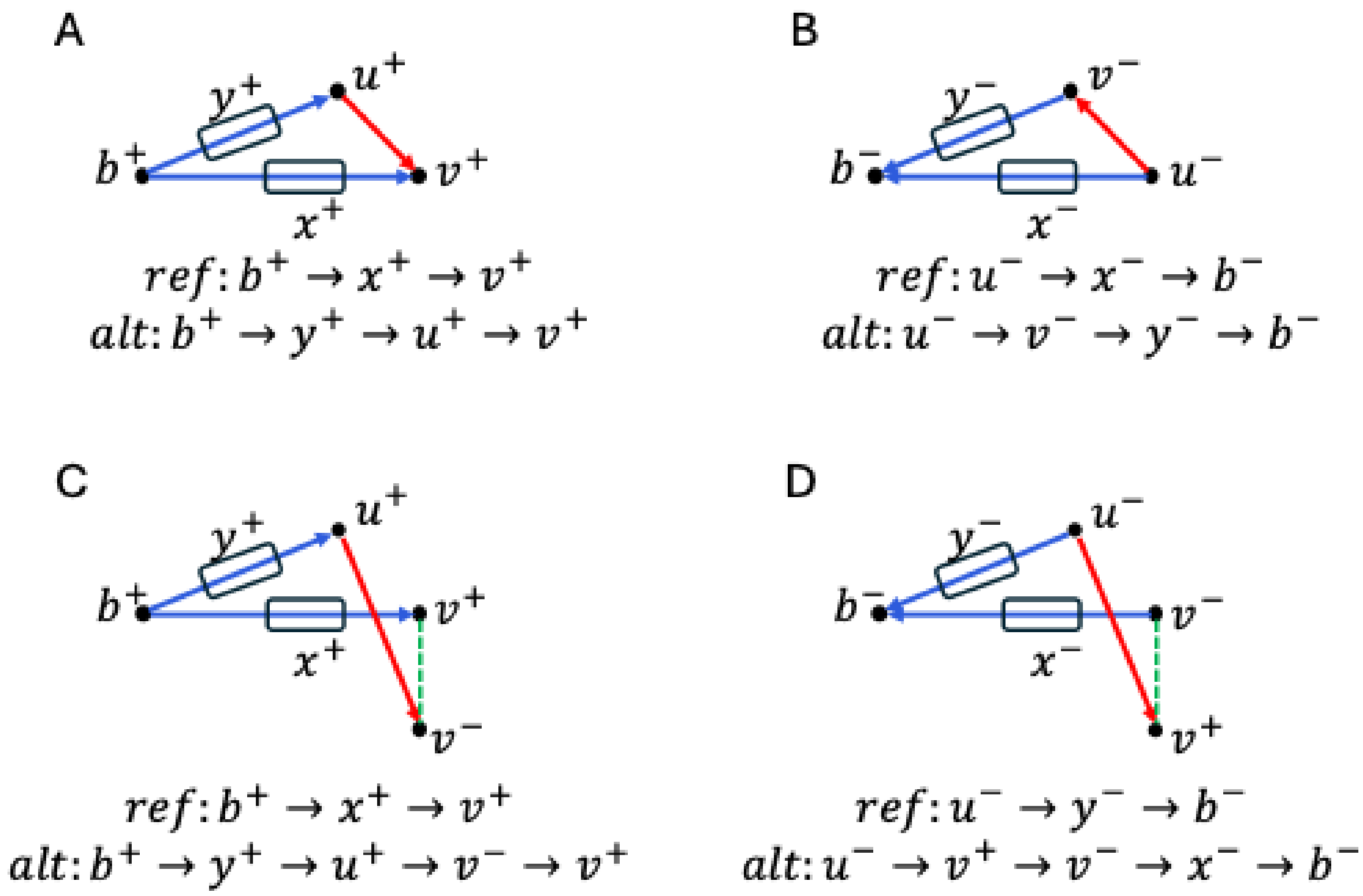

Figure S15. Reference and alternative paths for all orientations of a variant edge, related to STAR Methods. Each panel shows the reference path and the alternative path for a variant edge (red). Edges in the reference tree are shown in blue. Panels (A) and (B) illustrate cases where both endpoints of the variant edge lie in the same orientation: (A) forward ($u^+ \rightarrow v^+$) and (B) reverse ($u^- \rightarrow v^-$). In these cases, the branch point and both endpoints of the variant edge lie within the same half-tree. The two internally disjoint paths from the branch point to the variant endpoint form the reference and alternative paths, distinguished by whether or not they include the variant edge. Panels (C) and (D) illustrate inversion edges: (C) forward to reverse ($u^+ \rightarrow v^-$), and (D) reverse to forward ($u^- \rightarrow v^+$). In these cases, the second node of the variant edge lies in the opposite half-tree from the branch point and the first node. The green dashed undirected edge represents a switch within the binode that allows traversal between orientations. Reference and alternative paths remain a pair of internally disjoint parallel paths; the alternative path traverses both the variant edge and the green switch edge.

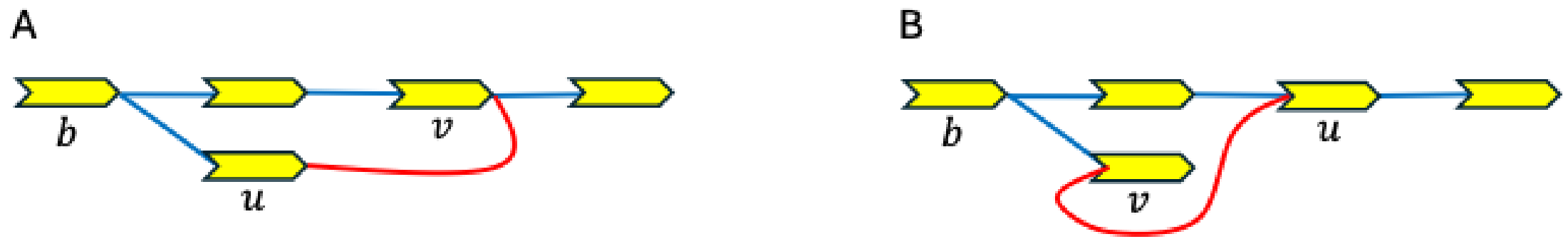

Figure S16. Illustration of allele simplification when a path visits both orientations of the same binode, related to STAR Methods. In both panels, the reference path (blue) follows the linear reference sequence. (A) The alternative path traverses nodes v and u^c but does not include both u and u^c or both v and v^c consecutively, so no cancellation occurs, and the alternative allele includes the sequence of u^c. This is visually clear from the figure since the alternative path enters and exits from different sides of binodes u and v when traversing the variant edge. (B) The alternative path contains both u, u^c or both v, v^c, and this results in a simplified allele with the corresponding segment omitted. This is visually clear since the alternative path enters and exits the same binode from the same side (either $u \rightarrow u^c$ or $v \rightarrow v^c$ depending on the orientation of the traversal).
